# Kinetically Divergent Dimanganese–Tyrosyl Radical Cofactor Assembly in *Listeria monocytogenes* Class I Ribonucleotide Reductase

**DOI:** 10.1021/acs.biochem.6c00369

**Published:** 2026-07-13

**Authors:** Paoning G. Chong, Saborni Biswas, Chihjung Chen, Joseph A. Cotruvo

**Affiliations:** Department of Chemistry, 8082 The Pennsylvania State University, University Park, Pennsylvania 16802, United States

## Abstract

Ribonucleotide reductases (RNRs) catalyze the reduction of nucleotides to deoxynucleotides in all organisms. Class I RNRs comprise two subunits, α2 (containing the active site) and β2 (containing the radical cofactor essential for initiating catalysis). The association of α2 and β2 is well known to regulate RNR activity, but a role for α2 in kinetically controlling cofactor assembly in β2 has not been reported. The class I RNR from the important human intracellular pathogen, *Listeria monocytogenes* (*Lmo*) is a member of the largest cluster of β subunit sequences (the “2RCC cluster”) remaining to be biochemically characterized. This protein was previously suggested to be a class Ia enzyme, active with a diferric-tyrosyl radical (Y•) cofactor. Here, we demonstrate that the *Lmo* class I RNR, in fact, is most active with a dimanganese-Y• cofactor, assembled with the help of the flavoprotein, NrdI. While superficially similar to previously characterized manganese-dependent class Ib RNRs, the *Lmo* RNR features a number of unusual properties. Most strikingly, the Mn^III^Mn^IV^ intermediate formed from reaction of the dimanganese­(II) cluster, NrdI, and O_2_ converts extremely slowly (*t*_1/2_ = 11 min) to an active dimanganese­(III)-Y• cofactor. However, in the presence of α2, cofactor assembly is accelerated by >10-fold (*t*_1/2_ = 0.6 min). Therefore, α2-driven conformational changes in *Lmo* β2 are likely required physiologically for not only catalysis but also activation. The properties of *Lmo*’s distinctive class Ib RNR expand the known mechanisms of RNR activity regulation and may have implications for the observed sensitivity of *L. monocytogenes* to iron excess during infection.

## Graphical abstract

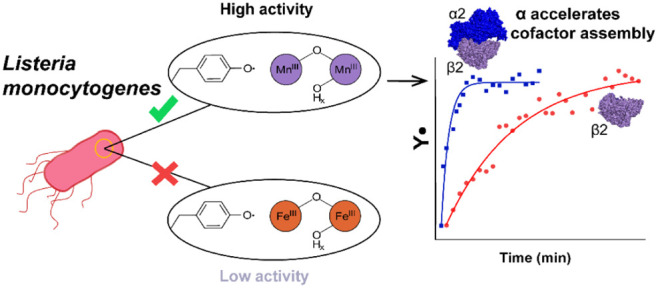

## Introduction

Ribonucleotide reductases (RNRs) catalyze the reduction of nucleotides to 2′-deoxynucleotides, serving as the only de novo source of deoxynucleotides for DNA replication and repair.^1,2^ Despite the central and essential role of these enzymes in life and their conserved active-site radical chemistry,^3,4^ organisms have evolved an array of radical-containing cofactors as repositories of the oxidizing equivalent that is used to generate, transiently and catalytically, the active-site thiyl radical that initiates nucleotide reduction. The class I RNRs, restricted to aerobes, are active as heterotetramers comprising two homodimeric subunits, α2 and β2,^5^ which interact transiently to mediate catalysis. Recent cryo-EM structures of the *Escherichia coli* class Ia RNR have revealed that the metallocofactor that stores the required oxidizing equivalent in the β subunit sits ∼32 Å from the active site in the α subunit (Figure 1A),^5,6^ connected by a conformationally gated, long-range proton-coupled electron transfer pathway that shuttles the oxidizing equivalent between subunits.^2^ In addition to being required for catalysis, interactions between α2 and β2 have also been shown to inhibit activity via the formation of a distinct α4β4 oligomeric state.^7^ By contrast, the potential contributions of α in regulating RNR activity via modulation of cofactor assembly in β have been largely unexplored.^8^ Here, we both reassign the identity of the likely physiological metallocofactor of the unusual class I RNR from *Listeria monocytogenes* and show that its assembly kinetics depend on α, suggesting a new regulatory role of α-β interactions in RNRs, which may have implications for pathogenesis.

**Figure 1:**
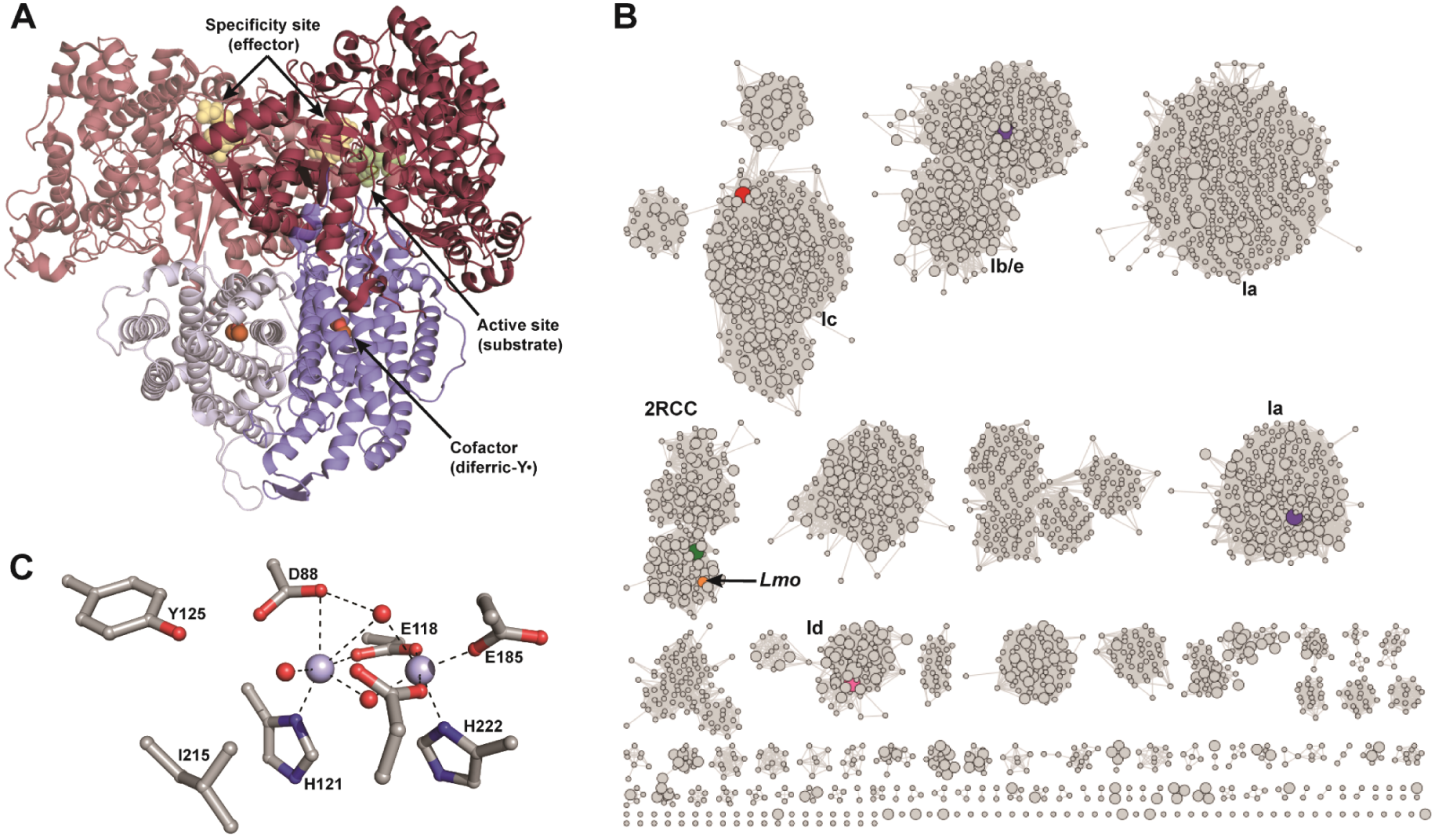
(A) The α2β2 holocomplex of the *E. coli*class Ia RNR, trapped with the mechanism-based inhibitor, 2′-azido-2′-deoxycytidine-5′-diphosphate (PDB code: 9DB2). The two chains of the α2 subunit are shown in light and dark maroon, with the effector ATP (tan) at the specificity site at the α dimer interface and the inhibitor (or inhibitor product complex) bound at each active site (green). The two chains of the β2 subunit are shown in gray and purple, with the diferric cluster of each β subunit shown in brown spheres. The darker α-β pair is engaged for turnover, with the active site ∼35 Å from the tyrosyl radical in β, while the lighter α-β pair is unengaged (and has already turned over). Adapted with permission from ref 6. (B) Sequence similarity network of class I RNR β subunit sequences. The network was developed using EFI-EST^34^ and constructed using Cytoscape^35^, with an alignment score of 80 and a node identity of 50%. Highlighted nodes include β subunits of *E. coli* (class Ia and Ib, purple), *B. halodurans* (green), *L. monocytogenes* (orange), *C. trachomatis* (class Ic, red), and *F. johnsoniae* (class Id, pink). Node sizes are scaled relative to number of organisms per node. (C) Detail of the dinuclear metal-binding site from the X-ray crystal structure of *B. halodurans* β (PDB 2RCC, chain B), modeled with Zn^II^ ions. Metal-binding residues and the presumptive Y•-harboring residue, Y125, are shown in sticks. Coordinated solvent and Zn^II^ ions are shown as red and purple spheres, respectively.

Class I RNRs are classified into five groups based on the identity of their radical-generating cofactors, which all require O_2_ for activation; cofactor utilization appears to reflect metal ion availability to an organism. Class Ia RNRs, found in “iron-centric”^9^ organisms from *E. coli* to humans, are activated by the reaction of the diferrous form of β with O_2_ to oxidize a neighboring tyrosine residue to a stable tyrosyl radical (Y•), forming a diferric-Y• cofactor.^10,11^ Many bacterial pathogens (and “manganese-centric” nonpathogens) have been shown in recent years to utilize Mn-containing RNRs (subclasses Ib-Id), which may reflect, in the case of pathogens, a mechanism to avoid iron restriction induced by the host during infection.^12–15^ Class Ib RNRs can be activated similarly using a diferric-Y• cofactor, which was originally thought to be the active form inside the cell.^16^ However, these systems also encode a flavodoxin-like protein, NrdI,^17^ which generates superoxide when in complex with the dimanganese­(II) form of β to assemble a dimanganese­(III)-Y• cofactor via a Mn^III^Mn^IV^ intermediate.^18–20^ In all cases to date, it is this dimanganese-Y• cofactor that is active in vivo,^21–26^ with no evidence of the diferric-Y• cofactor formed, perhaps due to low cytosolic iron levels when those systems are natively expressed. The class Ic enzyme from the intracellular pathogen, *Chlamydia trachomatis*, does not fully dispense with iron, but it lacks the analogous tyrosine and instead assembles a stable Mn^IV^Fe^III^ cofactor from Mn^II^Fe^II^ and O_2_ via a Mn^IV^Fe^IV^ intermediate.^200^ The class Ic RNR is the founding example of a growing class of heterodinuclear manganese–iron enzymes.^27–30^ Class Id RNRs use a Mn^III^Mn^IV^ cofactor, similar to the class Ib Tyr-oxidizing intermediate, but it is assembled using free superoxide without the involvement of a NrdI protein.^31,32^ Finally, the class Ie RNR cofactor does not contain a metal ion, and NrdI reacts with O_2_ to help generate a dihydroxyphenylalanine radical.^8,33^

The class Ia-Ie subclasses correlate with the major clusters in a sequence similarity network (SSN) of over 30,000 class I β subunit sequences (Figure 1B).^14,36^ The largest cluster of RNR sequences in the SSN yet to be assigned to a subclass is represented by the “2RCC cluster,” named after the PDB code for the β subunit of *Bacillus halodurans* (*Bha*), the only structurally characterized sequence in the cluster (Figure 1C). The 2RCC cluster comprises bacteria, including human and plant pathogens in the Listeriaceae (including *Listeria monocytogenes*) and Xanthomonadaceae (including *Xylella fastidiosa* and *Stenotrophomonas maltophilia*) families. At the time that the present work was initiated, only one class I RNR had been characterized biochemically from the 2RCC cluster, albeit cursorily: the single aerobic RNR from the *Listeria monocytogenes* (*Lmo*) reference strain EGD-e.^37^ *Lmo* is a Gram-positive, facultative anaerobic pathogen and the causative agent of the foodborne illness, listeriosis.^38–40^ The class I RNR gene cluster in this organism (Figure S1) encodes the α and β subunits, along with a NrdI-like protein and a thioredoxin-like protein, TrxL, which reduces α to allow for multiple turnovers. Aharonowitz and coworkers reported heterologous expression and purification of the *Lmo* class I RNR proteins from *E. coli* and assayed their activity. The activity of β was very low, <1% that of previously characterized RNRs.^41^ The enzyme was suggested to be a class Ia RNR with a diferric-Y• cofactor, although spectroscopic characterization was not reported, and despite NrdI being encoded in the gene cluster and its role in dimanganese-Y• cofactor assembly already having been demonstrated in the class Ib RNRs.^18^ These factors suggest that the active metallocofactor and subclass designation of the *Lmo* and other 2RCC cluster RNRs deserve reassessment.

The metal requirements of the *Lmo* RNR are of particular interest in the context of its pathogenesis and unusual life cycle. *Lmo* enters the host by ingestion of contaminated food and can then cross the intestinal epithelium, eventually invading the mesenteric lymph nodes and disseminating through the bloodstream, where it invades monocytes and macrophages.^42^ Iron starvation conditions, such as the vacuole formed in the invasion of a host cell, induce expression of listeriolysin O and the actin polymerase protein ActA, allowing for *Lmo* to escape the vacuole and enter the host cytosol, where it replicates^43^ and then can spread cell to cell.^38^ This life cycle juxtaposes two starkly different environments in terms of iron availability: an iron-limited endosome,^44^ and the cytosol where iron should be freely available. In fact, an Fe^II^-effluxing ATPase is critical to *Lmo* virulence in infection models,^45,46^ suggesting that *Lmo* experiences iron excess and toxicity at some point during pathogenesis.^47^ Unlike some organisms like *E. coli*, which possesses a class Ia RNR for iron-replete conditions and a class Ib RNR for iron scarcity, however, *Lmo* only has a single aerobic RNR. Given the central role of RNR in replication and therefore virulence, these considerations together indicate that determining whether the *Lmo* class I RNR is in fact iron-dependent, or perhaps manganese-dependent, is likely critical for an understanding of the organism’s pathogenesis.

Herein, we describe biochemical characterization of the *Lmo* class I RNR. We show that, although *Lmo* β can assemble a diferric-Y• cluster, the dimanganese­(III)-Y• cofactor, assembled with the help of NrdI, has seven times higher activity. Whereas these metal ion and NrdI requirements therefore appear to be similar to canonical class Ib RNRs, the *Lmo* enzyme exhibits a number of unusual features. Most importantly, its activation kinetics are so slow as to be unphysiological unless α is added, suggesting that α may play an indispensable role in accelerating cofactor assembly in vivo. These properties may reflect the need to achieve nucleotide reduction using a single aerobic RNR in a wide range of cellular iron and manganese concentrations.

## Materials and Methods

The complete Experimental Section, including detailed descriptions of all materials and experimental procedures associated with this work, can be found in the Supporting Information.

## Results

### *Lmo* RNR Forms Tyrosyl Radical from Diiron and Dimanganese Clusters

We initiated our studies by expressing and purifying the four components of the *Lmo* gene cluster, NrdA (named by Ofer et al.^37^ according to convention for a class Ia RNR, denoted herein as α for the monomer and α2 for the dimer), NrdB (named according to the class Ia RNR convention, denoted herein as β for the monomer and β2 for the dimer), NrdI (named according to the class Ib/Ie RNR convention), and TrxL (Figure S1). Purifications of these proteins had been mentioned previously^37^ but without any characterization beyond an activity assay of α and β. We found that TrxL could be purified easily, but the other three proteins required significant optimization beyond the prior report (Figure S2). Hexahistidine-tagged α underwent proteolysis during purification unless an N-terminal SUMO tag was added, and the yield was still very low (Figure S3). Although the SUMO tag could be cleaved with ULP1 protease, we found that the tag did not significantly impact activity, so it was retained for the sake of maximizing yield (see SI Experimental Section). As purified, over half of the NrdI protein lacked its flavin mononucleotide (FMN) cofactor,^17^ as evidenced by the ratio of absorbances at 280 nm (protein and FMN) and 454 nm (FMN), which was 10–11, compared to a theoretical ratio of 4.6 for fully FMN-bound NrdI. We found serendipitously during initial cluster assembly reactions that apo-NrdI, but not holo-NrdI (FMN-bound), precipitated at temperatures above 30 °C. Therefore, a mild heat treatment step was added to the purification protocol, which yielded A_280 nm/454 nm_ ratios of 5.5–7 (Figure S4), minimized instability, and improved Y• yield in cofactor assembly experiments. Reductive titrations of NrdI from the oxidized form to the hydroquinone form using dithionite occurred via a neutral semiquinone, as observed in previously characterized NrdIs (Figure S5).^18,19,25,48^ β was expressed without a purification tag (Figure S6) and its metalation state was sensitive to the expression conditions, as described below.

Previous characterization of the *Lmo* β, expressed heterologously in *E. coli* grown in Luria–Bertani (LB) media, revealed extremely low activity—2 U/mg with 2 mM dithiothreitol (DTT) as reductant, increasing to 7 U/mg with TrxL (1 U/mg = 1 nmol dCDP produced per min per mg β).^37^ By comparison, typical activities of other class I RNR β subunits with their endogenous reducing systems are 6000–8000 U/mg (*E. coli* class Ia diferric-Y•, 1.2 Y•/β2), 1000 U/mg (*B. subtilis* class Ib Mn^III^Mn^III^–Y•, 1.0 Y•/β2), and 600 U/mg (*C. trachomatis* class Ic RNR, 1.1 Mn^IV^Fe^III^/β2).^22,41,49^ Therefore, *Lmo* β did not form a significant amount of active cofactor during standard heterologous expression conditions. In our hands, expression of *Lmo* β in *E. coli* grown in LB media resulted in copurification of 1.03 ± 0.18 Fe/β2, 0.15 ± 0.04 Mn/β2, and 0.13 ± 0.04 Zn/β2 (Table S1). β purified in this manner contained Y• as detected by UV–visible spectrophotometry and continuous wave (CW) X-band EPR spectroscopy (Figure S7A, B). The UV–visible spectrum exhibited a sharp signal at 409 nm indicative of Y•; the *S* = 1/2 EPR signal at 77 K was centered at *g* = 2.005, closely reminiscent of the Y• in the *E. coli* class Ia RNR, but quantifying to just 0.04 Y•/β2 (Figure S7B).^10,50^ At lower temperatures, features similar to the one-electron-reduced Mn^III^Fe^III^ form of the *C. trachomatis* class Ic RNR became apparent, quantifying to 0.03 Mn^III^Fe^III^/β2 (Figure S7C, D). This quantification only accounts for ∼20% of copurified Mn; the remaining 80% therefore may be present in an EPR-silent form (e.g., Mn^IV^Fe^III^ or Mn^III^Fe^III^–Y•), but we did not pursue these possibilities as this β had very low activity. We also calculated that 0.61 Fe/β2 copurifies with the protein as *g* = 4.3 “junk iron,” supporting the idea that iron loading in *E. coli* is inefficient under these growth conditions (Figure S7E). The activity of *Lmo* β expressed in LB was negligible using our LC/MS method, whether or not TrxL was present in the activity assay (Table 1, Figure S8A).

**Table 1:** Activity Assays of *Lmo* β Reconstituted with Various Cofactors^h^

Sample	Specific activity, no TrxL	Specific activity, with TrxL	*n* ^a^	Y•/β2^b^	Activity with TrxL per Y•/β2^c^
Purified from LB^d^	N/A^e^	2 ± 7	2	0.04	ND
LB + 1,10-phen + Mn^II^, activated with NrdI	77 ± 57	194 ± 113	3	ND	ND
Apo + Fe^II^ + O_2_	31 ± 33	47 ± 62	2	0.25^f^	ND
Apo + Fe^II^ + O_2_ + S200	59 ± 1	162 ± 4	3	0.36 ± 0.06	455 ± 67
Apo + Mn^II^ + NrdI/O_2_	111 ± 11	941 ± 105	4	0.19 ± 0.04	5060 ± 560
Apo + Mn^II^ + NrdI/O_2_ + S200	208 ± 7	1226 ± 130	4	0.36 ± 0.05	3390 ± 360
Apo + Mn^II^ + Fe^II^ + NrdI/O_2_	101 ± 6	310 ± 21	3	0.11± 0.06	2790 ± 530
“Mn^III^Mn^IV^”^g^	ND	1440 ± 290	2	ND	ND

a *n =*number of biological replicates for activity assays.

b Y• content determined by EPR and UV–visible spectroscopy (if no uncertainty given, *n* = 1). ND, not determined; high-temperature X-band EPR was not conducted to quantify Y•.

c ND, not determined. Activity per Y• was not calculated either because Y• quantification was not carried out or because specific activity showed a large standard deviation.

d *Lmo* β purified from LB was not activated further by incubation with NrdI and O_2_.

e N/A, not applicable; no time-dependent dCDP production (see Figure S8A).

f Y• content immediately after cofactor assembly; decays over time (Figure S9B).

g Normal dimanganese assembly reaction but assayed immediately after O_2_ addition.

h Specific activities are calculated from the slope in U/mg β. Values reported as mean ± standard deviation.

Although the protein copurified with only 0.15 Mn/β2, this amount of Mn was surprisingly large, given that *E. coli* is an “iron-centric” bacterium with very low concentrations of total and labile manganese when grown in a rich medium like LB;^51–54^ this observation suggests that the protein has moderately high affinity and selectivity for Mn^II^ at (at least) one metal-binding site. Addition of 100 or 500 μM Mn^II^ to the growth media resulted in higher amounts of Mn and lower amounts of Fe copurifying with the protein (Table S1). Addition of 100 μM 1,10-phenanthroline (to restrict iron availability) and 200 μM Mn^II^ to the growth media resulted in almost fully Mn-bound protein and only 0.07 Fe/β2. When activated in vitro in the manner of class Ib RNRs with reduced NrdI (NrdI_hq_) and O_2_, this protein showed modest activity, 77 ± 57 U/mg β2 without TrxL but was stimulated to 194 ± 113 U/mg β2 with TrxL (Table 1). However, the heterogeneous metalation state of β necessitated preparation of apo-β for further studies to characterize the active cofactor(s). Apo-β was expressed in the presence of 100 μM 1,10-phenanthroline and 200 μM Mn^II^ and purified with 1 mM EDTA added to purification buffers, yielding protein copurifying with <0.05 equiv. each of Mn, Fe, and Zn per β2 (Table S1).

By analogy to in vitro reconstitutions of the class Ia and Ib RNRs, we incubated apo-β with 4 equiv. Fe^II^/β2 (2 Fe^II^ per β monomer) under anaerobic conditions at 4 °C and then added excess O_2_ in the form of O_2_-saturated buffer. UV–visible spectrophotometry revealed the characteristic features of a μ-oxo bridged diferric cluster and a sharp feature at 409 nm, indicative of Y• (Figure 2A, Figure S9A), and the EPR spectrum displayed the same class Ia-like Y• as observed in the protein as purified from LB (Figure 2B). The EPR signal quantified to 0.25 Y•/β2 immediately after assembly but, unlike most bacterial class Ia and Ib RNRs,^55^ in which the Y• is stable for hours to days, the Y• signal decayed to 68% of its original concentration after 30 min and 26% after 2 h at 21 °C (Figure S9B, C). Furthermore, when this protein was assayed with α immediately (within 1 min) after reconstitution, <50 U/mg activity was observed, regardless of the presence of TrxL. To investigate whether the instability of Y• and poor activity reflected inaccessibility of an optimal protein conformation for cofactor assembly at low temperature, the reconstitution was repeated with incubations at a more physiologically relevant temperature (37 °C), yielding similar results (Figure S9D). By contrast, when diferrous-β (prepared anaerobically) was instead directly loaded onto an aerobic gel filtration column and run at 4 °C, a peak with absorbance indicating a diferric-Y• cluster was isolated (Figure S10), with stable Y• (∼0.35 Y•/β2, Figure 2A) and activity of 59 ± 1 U/mg β2, enhanced to 162 ± 4 U/mg β2 with 10 μM TrxL (Figure S8B, Table 1). The unusual observation that bolus addition of excess O_2_ yields unstable Y• and protein with very low activity, whereas low O_2_ (or another oxidation mechanism that could occur on-column) yields stable, active cofactor, suggests that the *Lmo* β2 metal site may not be well equipped to react with O_2_. Using O_2_ as an oxidant would require provision of an electron to avoid an extra oxidizing equivalent at the metal site because metal cofactor and tyrosine oxidation require only 3 oxidizing equivalents.^50^ Using superoxide as an oxidant could obviate this problem (as it does in class Ib and Id RNRs),^14,19,31^ so we also investigated the reaction of the reduced, hydroquinone form of NrdI, NrdI_hq_, with O_2_ in the presence of diferrous-β. No Y• was observed by UV–visible or EPR spectroscopy, despite iron oxidation occurring within 1 min of O_2_ addition (Figure S11). The presence of *nrdI* in the *Lmo* RNR operon, yet the protein's inability to activate a diiron cofactor, further suggests that this cofactor is unlikely to be physiologically relevant for *Lmo* β.

**Figure 2:**
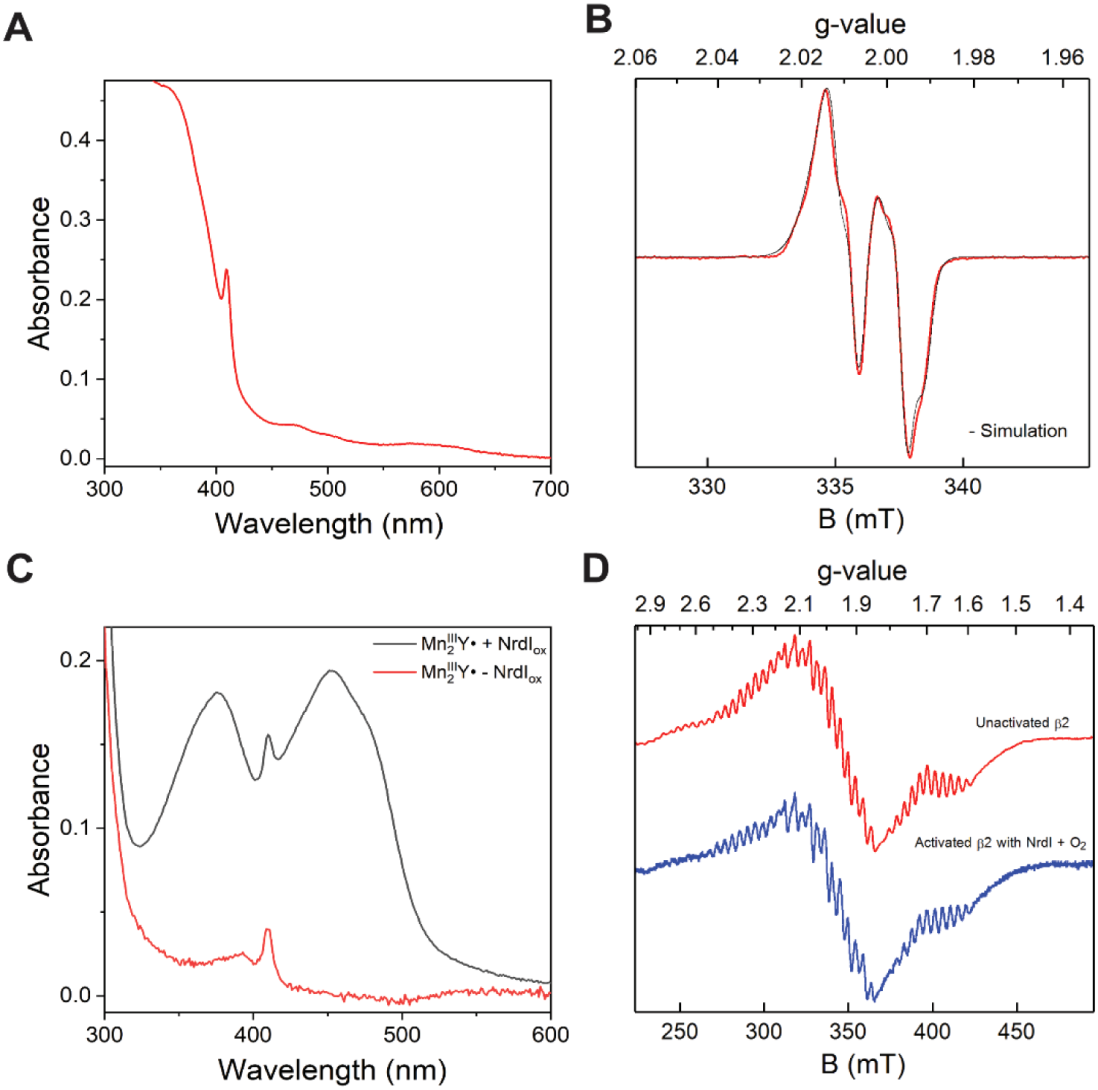
Characterization of *Lmo* RNR cofactors. (A) UV–visible absorption spectrum of diferric-Y• cofactor prepared by incubation of β with 4 Fe^II^/β2 for 10 min and activation by loading onto an aerobic size-exclusion column, from which fractions containing an absorbance peak at 409 nm were pooled (56 μM β2, 0.42 Y•/β2 for this particular sample). The 375 and 409 nm features are indicative of a μ-oxo-bridged diferric cofactor and Y•, respectively. (B) X-band EPR spectrum of diferric-Y• cofactor (red) and simulation (black dashed line, Figure S7, Table S2). The spectrum was collected at 77 K with 1 mW power and 0.35 mT modulation amplitude. (C) UV–visible spectrum of dimanganese-Y• cofactor (black) prepared by incubation of β with 3.4 Mn^II^/β2, mixing with 1.5 NrdI_hq_/β2, and activation with O_2_, followed by size-exclusion chromatography to partially remove NrdI and unreacted β (12 μM β2, 0.44 Y•/β2 for this particular sample). The red spectrum is after subtraction of NrdI_ox_ spectrum, showing the sharp Y• feature at 410 nm. The presumptive dimanganese­(III) cluster has a low extinction coefficient.^19^ (D) CW X-band EPR of 102 μM Mn^II^_2_-β2 (3.4 Mn^II^/β2) before activation (blue) and 95 min after reaction (red). Spectra were collected at 20 K with 1 mW power and 0.35 mT modulation amplitude.

Therefore, we investigated whether *Lmo* β could assemble a class Ib-like dimanganese-Y• cofactor. Apo-β was incubated with 4 equiv. Mn^II^/β2 and then activated with NrdI_hq_ (2 equiv. per β2) and excess O_2_ to yield a Y• which forms fully in 30 min as determined by UV–visible spectrophotometry, with a spectrum consistent with a dimanganese­(III)-Y• cofactor^18,19^ (Figure 2C). Y• was estimated by the dropline method, yielding ∼0.2 Y•/β2. We subsequently found that 1.5 NrdI_hq_ per β2 instead of 2 NrdI_hq_, followed by SEC after completion of activation to remove a portion of the unreacted β (Figure S12), yielded Y•/β2 content improved to 0.35–0.40 Y•/β2. The activity of the dimanganese-Y• cofactor was 208 ± 7 U/mg β2 without TrxL; assays with 10 μM TrxL yielded activities of 1226 ± 130 U/mg β2, 7-fold higher than the best observed with diferric-Y• assembly (Table 1, Figure S8C). Furthermore, TrxL also enhanced activity of the dimanganese-Y• cofactor to a greater extent than for the diferric-Y• cofactor. A manganese-containing cofactor accounts for the copurification of the protein with significant Mn even in rich media in *E. coli*and, more importantly, provides a rationale for the presence of *n* *rdI* in the gene cluster, presumably to form superoxide that is needed to react with a dimanganese­(II) cluster during assembly.

### Unusual Electronic Relaxation Behavior of the Dimanganese-Y• Cofactor

Despite the presence of the sharp absorbance feature associated with Y• at 410 nm (Figure 2C), CW X-band EPR spectroscopy of activated, dimanganese-Y• β2 did not yield any obvious signal characteristic of an organic radical distinguishable from the background at 20 or 77 K (Figure 2D). This observation is in contrast to all other RNRs activated with a dimanganese-Y• cluster that have been characterized by EPR spectroscopy: *E. coli*,^18^ *C. ammoniagenes*,^21^ *B. subtilis*,^19,24^ and *S. sanguinis*;^25^ of which the first three were shown to exhibit distinctive five-line patterns attributed to the interaction of the Y• with the dimanganese­(III) cluster. Instead, only a 40-line dimanganese­(II) signal and a broad underlying Mn-derived signal were observed, presumably from unreacted dimanganese­(II) cluster. In *E. coli* and *B. subtilis* class Ib RNRs, unreacted Mn^II^ ions can be removed by treatment with Chelex-100 or EDTA,^19^ in order to facilitate characterization of the active cofactor by EPR. When a similar procedure (10 min incubation with Chelex-100) was carried out with *Lmo* β2 that had been incubated first with 4 Mn^II^/β2, the EPR signal of the dimanganese­(II) cluster persisted but signals associated with mononuclear (likely unbound) Mn^II^ ions were removed, leaving 3.40 ± 0.17 Mn/β2, without diminishing the coupled dimanganese­(II) EPR signal (Figure S13). Extended incubation of the unactivated Mn^II^_2_-β with Chelex-100 only slowly removes Mn^II^ ions, with 2.2 ± 0.1 Mn/β2 remaining after 2 h (Figure S13B). This unexpected result indicates exceptionally tight Mn^II^ binding or, perhaps more likely, a kinetic limitation to complete metal removal (*vide infra*). A similar cluster without a mononuclear Mn^II^ EPR signal could also be prepared by adding 3.4 equiv of Mn^II^ to apo-β without Chelex-100 treatment (Figure 2D, Figure S14).

The ∼40-line dimanganese­(II) cluster signal (Figure 2D) exhibits similarities to those of the class Ib β subunits of *E. coli* and *S. sanguinis*.^18,25^ This spectrum shows several resonances across the field range of 200–500 mT, with features at around 250–300 mT and 370–420 mT containing hyperfine splitting with an approximate value of 4.6 mT. These hyperfine interactions are consistent with hyperfine interactions from two nearly equivalent Mn^II^ (*I* = 5/2) ions with a typical A_Mn_ ∼ 250 MHz.^56,57^ As the temperature increases, the total integrated intensity of the dimanganese­(II) cluster signal increases (Figure S15), which is similar to the observation in the *B. subtilis* RNR.^19^ The trend in the *Lmo* protein is indicative of an antiferromagnetically coupled ground state, with *J* < 0 (using the Heisenberg–Dirac-van Vleck Hamiltonian for exchange, Ĥ = −2JS_1_S_2_). As the temperature increases, the signal intensity increases, suggesting an EPR-active, higher spin manifold (S_T_ ≠ 0) with S_T_ = 0 as the ground state.^57^

The Mn^II^-derived signals could not be removed from the protein with the assembled dimanganese-Y• cofactor by longer Chelex-100 treatment without concomitant loss of Y• signal, precluding analysis of the Y• in this temperature regime. The Y• was not clearly observed until studies in the range of 70–200 K revealed an organic radical centered at *g* = 2.008 (Figure 3), which did not saturate even at 200 K with a microwave power of 1 mW. This temperature-dependent behavior is distinct from previously characterized class Ib dimanganese-Y• cofactors, which have been observed at 0.1 mW at 10 K (*B. subtilis*) and at 20 K (*E. coli*),^18,19^ and at 2 mW between 5 and 80 K (*C. ammoniagenes*).^21^ While we cannot rule out alternative explanations based on the low-temperature spectra alone, the temperature dependence is suggestive of stronger effective magnetic coupling between the dimanganese­(III) cluster and the Y• in *Lmo* β than in the canonical class Ib systems. Under this interpretation, the radical’s relaxation would be sufficiently enhanced by coupling to the metal-based spin system such that the signal is broadened beyond detection at low temperature; as the temperature is raised, fast electronic relaxation of the dimanganese cluster would progressively weaken the coupling, and the contributions of the Mn^II^-derived signals diminish, allowing the Y• signal to emerge. Efforts to isolate the dimanganese-Y• from the underlying multiline dimanganese­(II) signal and to characterize the fast-relaxing Y• in detail are presented in the Supplementary Results and Figure S16.

**Figure 3:**
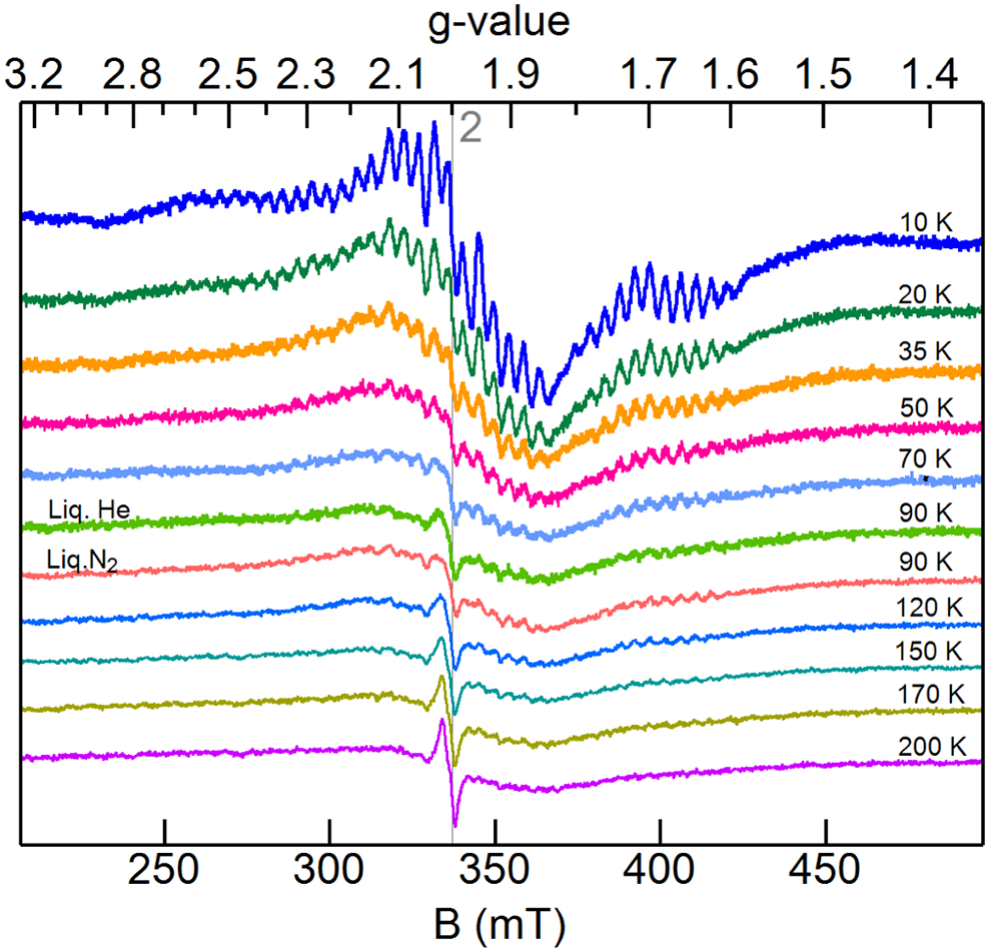
Unusual temperature dependence of the EPR spectra of Mn^II^Mn^II^ and dimanganese-Y• clusters in *Lmo* RNR. Temperature-dependent X-band EPR spectra of the activated dimanganese-Y• cofactor prepared by addition of 3.4 Mn^II^/β2 and 2 NrdI_hq_/β2 and excess O_2_, frozen 90 min after addition of O_2_ following SEC. Temperatures from 10 to 90 K were run with liquid He, and temperatures from 90 to 200 K were run with liquid N_2_. All spectra were collected with 1 mW power and 0.35 mT modulation amplitude.

The Y• spectrum at 150 K could be qualitatively simulated using *S* = 1/2 and *g* = [2.026, 2.003, 1.988] (Figure S17), consistent with a tyrosyl-like organic radical. However, persistent overlap with the underlying dimanganese­(II) signal at these elevated temperatures precludes reliable deconvolution of the radical line shape; quantitative hyperfine coupling constants and β-proton dihedral angles were therefore not extracted. The temperature-dependent behavior nonetheless establishes the presence of a tyrosyl radical magnetically coupled to the dimanganese cluster.

### Slow Tyrosine Oxidation via a Mn^III^Mn^IV^ Intermediate

Based on the characterization of the dimanganese­(III)-Y• cofactors of class Ib RNRs, formed via oxidation of their dimanganese­(II) clusters by NrdI-produced superoxide via a Mn^III^Mn^IV^ intermediate,^19^ we suspected that the manganese-loaded *Lmo* RNR formed its cofactor by the same mechanism. Furthermore, we aimed to verify that the manganese ions were in the +III oxidation state in the active cofactor, but this assignment could not be made definitively for two reasons. First, the broad EPR features associated with the dimanganese­(II) cluster did not disappear appreciably after activation (Figure 2D). Second, UV–visible absorption features of the expected dimanganese­(III) cluster would be weak^19^ and are not clearly observable above the NrdI spectrum (Figure 2C); chromatographic separation of NrdI from β2 following activation was incomplete (Figure S12).

To address both matters, we carried out assembly reactions, hand-quenched in liquid nitrogen between 44 s and 30 min after O_2_ addition. The series of spectra shown in Figure 4A and Figure 4B were measured by X-band EPR at 20 and 150 K, respectively. For reactions trapped between 44 s and 10 min, a 16-line spectrum was observed at 20 K (Figure 4A), similar to the strongly coupled Mn^III^Mn^IV^ intermediate in *B. subtilis* class Ib RNR,^19^ along with a residual amount of Mn^II^_2_-β2 signal. The presence of the background signal shows that a large portion of the dimanganese­(II) cluster is unreactive (as has been observed in class Ib RNRs unless multiple rounds of activation with NrdI are carried out).^41^ The maximum intensity of the 16-line feature is seen in the first time point, at 44 s. Simulations that best fit the 44 s spectrum were obtained after subtracting a spectrum of the starting unreacted dimanganese­(II)-β after normalization to the sample concentration. This subtraction largely removes the background features, except in the low-field range of 266–296 mT, resulting in misalignment of the simulation in this region (Figure S18). The simulation required two^55^ Mn ions with the hyperfine coupling constants A of [−454, −454, −339] MHz and [229, 299, 263] MHz for the Mn^III^ and Mn^IV^ centers, respectively. These values are those determined for the *B. subtilis* Mn^III^Mn^IV^ intermediate and the superoxidized form of manganese catalase.^19,58^

**Figure 4:**
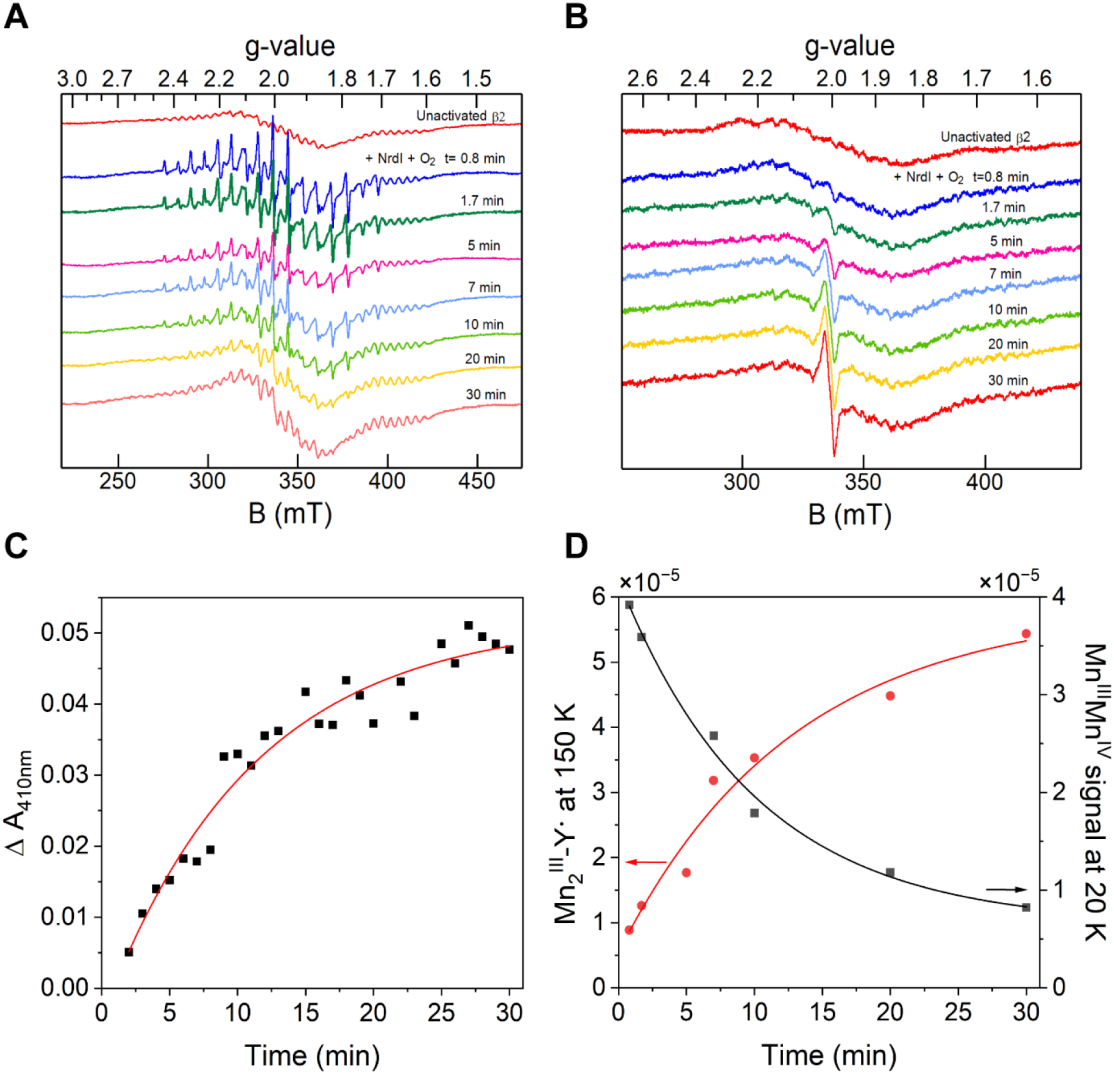
EPR studies of dimanganese-Y• cofactor assembly kinetics. CW X-band EPR spectra of the dimanganese-Y• cofactor assembly frozen at various time points. Mn^II^_2_-β2 (70 μM β2) was activated with NrdI_hq_ and O_2_-saturated buffer at 25 °C and frozen with liquid N_2_ at 44 s, 95 s, 3, 5, 7, 10, 20, and 30 min. Spectra were collected with 1 mW power and 0.35 mT modulation amplitude at (A) 20 K and (B) 150 K. (C) UV–visible absorbance at 410 nm of dimanganese-Y• cofactor assembly run in parallel with the samples quenched for X-band EPR. (D) Kinetic traces of the formation of Y• and the decay of Mn^III^Mn^IV^ as quantified by EPR, each fitted to a single exponential. See Figure S20 for results from a replicate experiment.

Tyrosyl radical formation was followed at 150 K (Figure 4B). The Y• signal at *g* ∼2.01 grows in over 30 min; while a small signal is present at the first time point, it cannot be easily differentiated from the broad Mn^II^Mn^II^ background and quantified. The intensities of the Mn^III^Mn^IV^ signal decay at 20 K and Y• formation at 150 K can both be fitted to first-order exponential kinetics with similar rate constants, ∼0.0015 s^–1^ (Figure 4D, Table 2, Table S3). These rate constants agree with kinetic monitoring of the increase in A_410 nm_ associated with Y• formation by UV–visible spectrophotometry (Figure 4C). These plots indicate that the Mn^III^Mn^IV^ intermediate is kinetically competent to serve as the direct oxidant for Y• formation, such that the Y• is associated with a Mn^III^Mn^III^ cluster.

**Table 2:** Rate Constants for Intermediate Decay and Active Cofactor Formation in *Lmo* RNR and Other Kinetically Characterized Class I RNRs^d^

RNR	Reaction	*k* (s^–1^)	*t*_1/2_ (min)	Reference
*Lmo* class I	Mn^III^Mn^IV^ decay	0.0016 ± 0.0005^a^	7.8 ± 2.2	This work
	Mn^III^_2_-Y• formation	0.0013 ± 0.0003^b^ (UV-vis)	9.4 ± 1.8	
		0.0008 ± 0.0005^a^ (EPR)	15.4 ± 7.1	
	Mn^III^_2_-Y• formation in presence of SUMO-α and ATP^b^	0.0090 ± 0.0008 (UV-vis)	1.3 ± 0.1	
	Mn^III^_2_-Y• formation in presence of SUMO-α, CDP, and ATP^b^	0.0245 ± 0.0051 (UV-vis)	0.6 ± 0.1	
	Mn^III^_2_-Y• formation (37 °C)^c^	0.0036 ± 0.0004	3.2 ± 0.6	
*B. subtilis* class Ib	Mn^III^Mn^IV^ decay	0.12 ± 0.02	0.10 ± 0.02	Cotruvo et al.^19^
	Mn^III^_2_-Y• formation	0.08–0.15	0.08–0.15	
*E. coli* class Ia	Fe^III^_2_-Y• formation	0.7–1.0	0.012 – 0.02	Bollinger et al.^11^
*M. musculus* class Ia	Fe^III^_2_-Y• formation	5 ± 2	0.0023 ± 0.0012	Yun et al.^59^
*C. trachomatis* class Ic	Mn^IV^Fe^IV^ decay	0.7 ± 0.1	0.017 ± 0.003	Jiang et al.^60^

a Mean ± standard deviation of three replicates; individual data sets are provided in Table S3.

b Mean ± standard deviation of two replicates; individual data sets are provided in Table S3.

c Single experiment; uncertainty reported is the uncertainty in the fit.

d Class Ia and Ic rate constants were determined at 5 °C, whereas class Ib and *Lmo* RNR rate constants were determined at 25 °C (unless otherwise noted).

Although cofactor assembly seems to follow a class Ib-like pathway via an Mn^III^Mn^IV^ intermediate, the kinetics are vastly different for *Lmo* class I RNR. The rate constants for the decay of the Mn^III^Mn^IV^ signal (*t*_1/2_ ∼ 7.8 min) and for the formation of the Mn^III^_2_-Y• signal (*t*_1/2_ ∼ 11 min) are almost 100-fold slower in *Lmo* than the *B. subtilis* class Ib RNR (*t*_1/2_ ∼ 0.08–0.15 min),^19^ the only class Ib RNR for which assembly kinetics have been studied in detail previously. Dimanganese-Y• cofactor assembly in *B. subtilis* is, in turn, >10 times slower than the rate constants for activation of class Ia and Ic RNRs (Table 2). Incubating the assembly reaction at 37 °C (Figure S19) rather than at 25 °C increases the rate constant for Y• formation by approximately 2-fold (0.0036 s^–1^, *t*_1/2_ = 3.2 min), but this is still much slower than previously characterized RNRs. Unless we are missing an accessory factor in addition to NrdI, activation of the *Lmo* RNR with a dimanganese cluster is curiously on the order of the bacterial doubling time.^61^

### Attempts at Heterodinuclear Cluster Assembly in *Lmo* β

The extremely slow Y• formation observed in dimanganese-Y• cofactor assembly led us to investigate other possibilities for activation. Because early observations had indicated the ability of *Lmo* β to form small amounts of heterodinuclear Mn^III^Fe^III^ clusters inside the cell (Figure S7) and to copurify with significant amounts of Zn (Table S1, Figure 1), we tested three types of heterodinuclear cofactor assembly in vitro (Supplementary Results, Figure S21).

We first considered the possibility of a mononuclear Mn site allowing for Y• formation, with the other site filled by a redox-inert Zn^II^ ion. By the addition of 3.4 equiv. Mn^II^ followed by Zn^II^, we were able to form a Mn^II^Zn^II^ cluster (Table S4, Figure S21A), suggesting that the Mn^II^ ion in one site is kinetically inert, but the other can be more readily displaced; however, this cluster could not be activated by NrdI_hq_ and O_2_ (Figure S21B). These results likely eliminated the possibility of an active mononuclear manganese cofactor and established the strong preference of one metal site for Mn^II^. Using this information, we also attempted to form a Mn/Fe cluster by first incubating the protein with Mn^II^, followed by Fe^II^, then NrdI_hq_ and O_2_. The low-temperature EPR spectrum revealed features characteristic of a Mn^III^Fe^III^ cluster,^21^ and some formation of Y• visible only at higher temperatures and therefore suggestive of dimanganese-Y• but not diferric-Y• cofactor formation (Figure S21C, D). TrxL-stimulated activity per Y• was somewhat lower than with the dimanganese-Y• assembly (Table 1, Figure S22), making it unlikely that some of the observed activity arose from a form of the MnFe cluster. Finally, for the sake of completeness, we also tested whether an Fe/Zn cluster (1.7 equiv. Fe^II^ followed by 1.7 equiv. Zn^II^) could support Y• formation in the presence of NrdI and O_2_; no Y• was observed. These experiments confirmed the relevance of the dimanganese cofactor, although its assembly kinetics were still perplexing.

### The α Subunit Accelerates Dimanganese Cofactor Assembly

We next considered whether the long-lived Mn^III^Mn^IV^ species, which is kinetically competent for tyrosine oxidation, could also support activity, as in the class Id RNRs.^31^ To assess this hypothesis, we added α to initiate activity assays as soon as possible (∼15 s) after the introduction of O_2_ to assemble the cofactor. At this time point in the assembly reaction, negligible Y• was expected to have been formed (Figure 4). Unexpectedly, we observed that the specific activity of the protein was similar to that observed when activity assays are initiated 60 min after O_2_ introduction to ensure full formation of the dimanganese (III)-Y• cofactor (Table 1).

We proposed two potential explanations for this surprising result. First, the Mn^III^Mn^IV^ form of the cluster may be stabilized in the presence of α and indeed enable RNR activity. Second, the presence of α could conceivably accelerate the conversion of the Mn^III^Mn^IV^ intermediate to the active dimanganese­(III)-Y• form. To distinguish between these possibilities, we carried out an assembly reaction in which α, CDP (substrate), and ATP (allosteric effector) were added as soon as possible (∼12 s) after O_2_ addition to the incubation mixture of Mn^II^_2_-β2 and NrdI_hq_ to initiate cluster assembly. Formation of Y• was followed by absorbance at 410 nm. Y• was already visible at the fastest time point (12 s after α addition) and was complete within <5 min (Figure 5), yielding a rate constant for Y• formation more than 10-fold faster than in the absence of α (0.0245 s^–1^, Table 2). To exclude the possibility that this acceleration could be explained by radical translocation from Mn^III^Mn^IV^ leading to turnover in the presence of α, substrate, and effector, with the radical returning to tyrosine rather than to the dimanganese site, we repeated the assembly with only α and ATP added (omitting CDP). Y• formation proceeded with a rate constant of 0.0090 s^–1^ (*t*_1/2_ = 1.3 min, Table 2), slightly slower than in the presence of substrate but still 10-fold faster than without α. Therefore, we conclude that 1) Mn^III^Mn^IV^ is unlikely to be involved in turnover, and 2) α (in the presence of effector) accelerates cofactor assembly in *Lmo* RNR, to a rate that is within 1 order of magnitude of *B. subtilis* class Ib RNR and more likely in a physiologically relevant kinetic regime. The large effect of α on assembly kinetics is unusual among RNRs and suggests a novel mechanism of regulation of cofactor assembly and thus of RNR activity.

**Figure 5:**
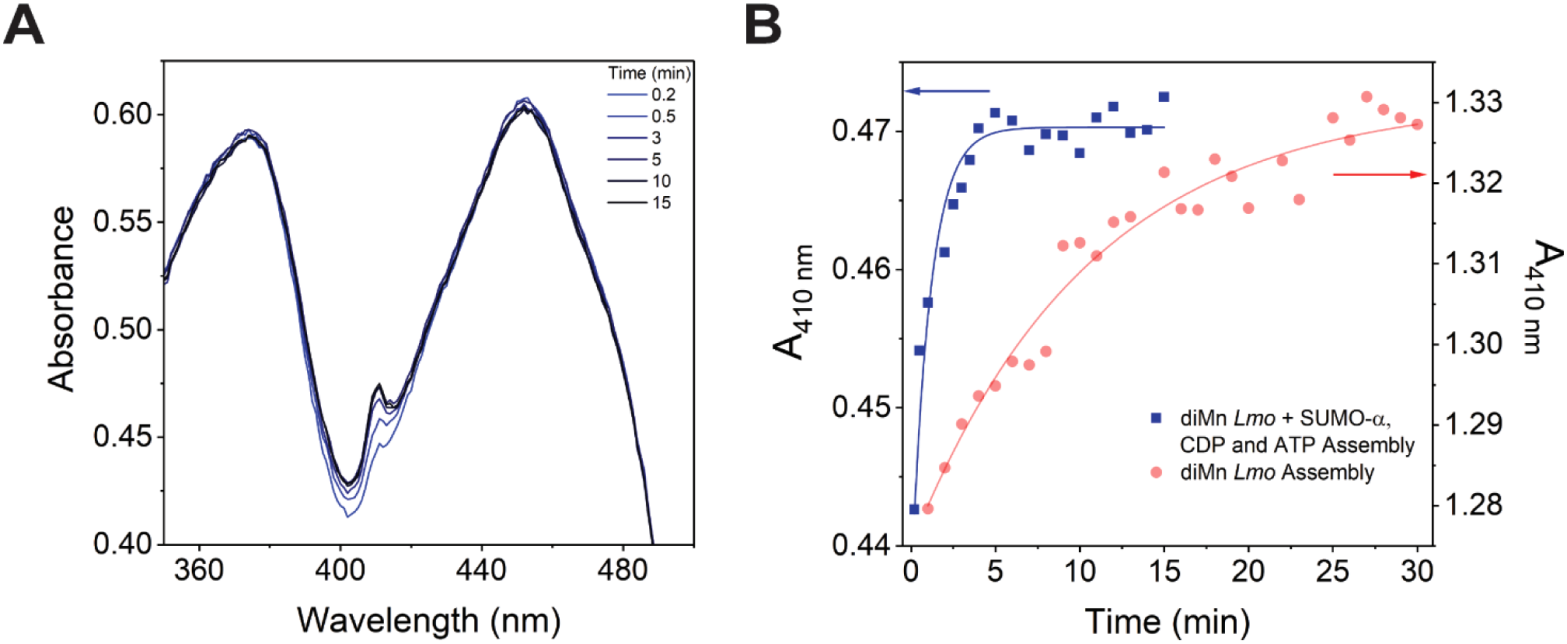
Acceleration of dimanganese-Y• assembly in the presence of α. (A) UV–visible spectra showing growth of dimanganese-Y• in the presence of SUMO-α, CDP, and ATP over time. The reaction consisted of 22 μM Mn^II^_2_-β2, and 44 μM NrdI_hq_, mixed with O_2_, followed by the addition of SUMO-α (44 μM), CDP (2 mM), and ATP (2 mM). (B) Absorbance at 410 nm of the dimanganese-Y• cofactor assembly reaction in the absence (red) and presence (blue) of SUMO-α, CDP, and ATP. *Y*-axes are scaled to reflect the different protein concentrations in the two experiments (22 μM β2 and 44 μM NrdI in the presence of α, versus 70 μM β2 and 140 μM NrdI in the absence of α).

## Discussion

Our work strongly supports the relevance of a dimanganese-Y• cofactor in *Lmo* class I RNR. NrdI facilitates the assembly of an active dimanganese­(III)-Y• cofactor that yields activity (when normalized per Y•) roughly comparable to the canonical *E. coli* class Ia enzyme (Table 1). TrxL has a large stimulatory effect on activity, whereas it does not for iron. The relatively low Y• content is typical of NrdI-based activations of dimanganese-Y• cofactors^18,19,24,25,41^ even when the physiological relevance of that cofactor is well established. Conversely, a number of our observations suggest that the previously proposed diferric-Y• cofactor^37^ is of little relevance physiologically. In iron-replete *E. coli*, the diferric-Y• cofactor is poorly assembled, and the protein purifies with substantial Zn bound, suggestive of low levels of the physiologically relevant metal ion (*E. coli* imports little Mn under these conditions) and poor Fe^II^ binding. In vitro, Y• associated with the diferric cluster, formed during standard class I RNR assembly conditions, is unstable and has low specific activity. We speculate that this Y• instability may reflect the protein not being equipped to provide an extra reducing equivalent during cofactor assembly, as occurs in the class Ia and Ic RNRs. In class Ib RNRs, the requisite 3 oxidizing equivalents are provided by superoxide generated by NrdI, and the presence of the *nrdI* gene in the *Lmo* RNR operon also argues against a diferric-Y• cofactor. When diferric-Y• cofactor assembly was attempted in the presence of NrdI, we observed full inhibition of Y• formation, even greater than the inhibitory effect observed in the *E. coli* class Ib RNR.^18^ Together, all of these observations lead us to reject the original class Ia designation in favor of reassignment of the *Lmo* enzyme as a class Ib RNR. Accordingly, we suggest that the *Lmo nrdA* and *nrdB* genes be renamed *nrdE* and *nrdF*, respectively, according to the class Ib convention.

*Lmo* β2 is also atypical, however, in three important regards: its slow Mn^II^ dissociation kinetics, the sluggish kinetics of tyrosine oxidation in dimanganese-Y• assembly (Table 2), and the unusual relaxation properties of the dimanganese­(III)-coupled tyrosyl radical. While the latter observation requires structural characterization for further conclusions to be drawn, we discuss here how the former two observations may be functionally and physiologically related.

Previously characterized class Ib RNR β subunits (NrdF) have exhibited a range of affinities for Mn^II^ but are unified in the Mn^II^ sites being kinetically labile. The dissociation constant (*K*_d_) of Mn^II^-bound *S. sanguinis* NrdF has been reported to be 0.4–5 μM.^62^ *E. coli* NrdF can be stoichiometrically loaded with Mn^II^ even at low protein concentrations,^19^ suggesting a similar (or tighter) *K*_d_, although a value has not been reported. *B. subtilis* NrdF binds Mn^II^ significantly more weakly.^19^ Regardless of these proteins’ Mn^II^ affinity, however, the addition of Chelex-100 or EDTA rapidly removes Mn^II^ that is unreacted after cluster assembly, such that the EPR spectra of the dimanganese-Y• cofactor can be cleanly prepared.^18,25^ These proteins do not show clear selectivity for Mn^II^ over Fe^II^ based on binding affinity alone, which probably reflects low bioavailability of Fe^II^ in these organisms; the biology of *B. subtilis* is manganese-centric,^9^ *S. sanguinis* readily accumulates Mn^II^,^63^ and *E. coli* (despite being iron-centric) expresses its class Ib RNR only in severely iron-limited or oxidative stress conditions where Mn predominates. *B. anthracis* NrdF has been suggested to exhibit partial intrinsic selectivity for Mn^II^ over Fe^II^, but the study was conducted *in crystallo* rather than in solution.^26^

In this context, the observation that short incubations of *Lmo* β with Chelex-100 only remove unbound Mn^II^, and even a 2-h incubation only depletes half of the bound Mn^II^, was surprising (Figure S13). This observation indicates that both metal sites are more kinetically inert than in previously studied Mn-dependent RNRs. Nevertheless, one Mn^II^ site appears to be more inert than the other, as one site can be exchanged to form heterobimetallic clusters: with Zn^II^ to form Mn^II^Zn^II^ clusters, and (to a lesser extent) with Fe^II^ to form Mn^II^Fe^II^ clusters that can be oxidized to a Mn^III^Fe^III^ state (Figure S21). The capacity to form these heterodinuclear metalated states may be governed by the solvent accessibility of one or both of the metal-binding sites. In the X-ray structure of *Lmo*’s 2RCC cluster relative *Bha* β, in which one chain is in the apo state, core helix 1 is unwound from D88 to D93 in the apo chain but not the metal-bound chain, resulting in a ∼300̵Å^3^ cavity that exposes metal site 1 to solvent (Figure S24). D88 is one of the ligands to site 1, the metal site nearest to the radical-harboring Tyr (Figure 1C); thus, metal ion binding would seem to be necessary to wind the D88-D93 helical region and protect the metal site and tyrosine from solvent prior to tyrosyl radical generation. If retained in *Lmo* β, as expected, this conformational change in turn might provide a kinetic barrier to the dissociation of bound metal ions. Unwinding of the analogous helix in the apo form of *Francisella hispaniensis* β (a “divergent” class Ia) has also been observed, with hydrogen–deuterium exchange mass spectrometry (HDX-MS) suggesting that metal ion binding induces a conformational change.^64^ Class Id β subunits exhibit flexibility in this region as well, albeit to expose the dimanganese­(II) cofactor to enable access of superoxide during cofactor assembly.^31,65^ Similar X-ray and MS studies of the *Lmo* system would indicate whether different metal occupancy induces distinct conformational dynamics, perhaps also accounting for *Lmo* β’s poor diferric-Y• assembly and activity in vivo and in vitro.

Full dimanganese-Y• assembly requires approximately 30 min in *Lmo* RNR, compared to <40 s for *B. subtilis* NrdF, which itself is substantially slower than class Ia and Ic RNRs (Table 2).^19^ This extremely slow assembly led us to investigate the potential activity of the Mn^III^Mn^IV^ intermediate itself, which, in turn, led us to the discovery that α (in the presence of ATP) increases the rate of Y• formation by >10-fold, into a range that is more likely physiologically relevant. The Mn^III^Mn^IV^ intermediate is formed in our in vitro assemblies faster than α addition, so the effect of α is not to accelerate formation of this intermediate but rather to accelerate its conversion to the dimanganese-Y• cofactor. We are not aware of an RNR for which α has been shown to enhance cofactor assembly kinetics. In class Ie RNRs, coexpression of α slightly increases the yield of dihydroxyphenylalanine radical in cells, but the effect is modest, and no kinetic information is available.^8^ Since α would likely be present when β is being translated, our observation raises the possibility that α might also influence the metal selectivity of β by altering its conformational dynamics, such as via the putative unwound helical segment near site 1. Alternatively, if dissociation of NrdI is a requirement for the completion of cofactor assembly, α binding might facilitate that process; however, structural alignment suggests that the binding mode of NrdI observed in its complexes with class Ib β2’s^20^ is not incompatible with complexation with α2, at least for the half of the complex that is engaged in electron transfer.^5,6^ While the difficulty of obtaining large amounts of α made it challenging to assess multiple conditions beyond the results shown herein, our work motivates further biochemical and structural studies to interrogate the mechanism of α’s effect on cofactor assembly in the *Lmo* RNR. It also prompts investigation of whether α may influence cofactor assembly in other RNRs.

We propose that the unusual kinetic properties of the *Lmo* class Ib RNR in metal binding and assembly together may reflect a mechanism of ensuring correct metalation of an essential enzyme, despite potentially large shifts in manganese vs iron availability, such as during the course of infection. While perhaps “manganese-centric” in general, *Lmo*’s unusual life cycle also potentially puts it at particular risk of iron toxicity. Indeed, *Lmo*’s Fe^II^-effluxing P_1B4_-type ATPase, FrvA, has been demonstrated to be critical to virulence in infection models.^45,46^ Some *Lmo* isolates harbor an additional P_1B4_-type ATPase on an accessory plasmid, putatively regulated by a riboswitch that responds to Fe^II^ in vitro.^66,67^ Thus, avoiding mismetalation of its manganese-dependent, sole aerobic RNR with iron upon cytosolic entry may be one of the compelling reasons for *Lmo* using these iron exporters to maintain low intracellular labile iron levels, even when iron is abundant extracellularly. Conversely, although Mn acquisition and function in *Lmo* have not been studied as extensively as iron homeostasis, all three components of the ABC transporter mediating Mn^II^ uptake are downregulated inside the host cytosol, where RNR activity would presumably be important for bacterial proliferation.^68^ Further insight into the metal preferences of the 2RCC cluster RNRs may help us to better understand this interplay of iron and manganese homeostasis in pathogens like *Lmo* and may provide new strategies for combating infections caused by these organisms.

Finally, it is tempting to propose that other 2RCC cluster members will have similar properties: Mn-dependence of activity, α-dependence of assembly kinetics, kinetic inertness of the dimanganese­(II) form. Many of the organisms whose putative RNR β subunits are in this cluster, including Listeriaceae, also encode putative NrdIs, and it seems likely that our results will extend to those systems. The cluster also contains the β of *Campylobacter jejuni*, another foodborne pathogen, the genome of which does not encode a NrdI. While our study was in progress, *C. jejuni* was shown to possess a different flavodoxin that was reported to enhance RNR reactivation with iron.^69^ In that study, the *C. jejuni* β was purified from *E. coli* with a non-negligible amount of Mn, but assembly of a diferric-Y• cofactor was the focus, and a potential role of Mn in activating this RNR was not examined, which should be reassessed in light of our results. Of course, the 2RCC cluster also contains the *B. halodurans* β. *B. halodurans* was recently reclassified first as *Alkalihalobacillus halodurans* ^70^ and later *Halalkibacterium halodurans*.^71^ *B. halodurans* does not encode a NrdI-like protein adjacent to the RNR operon, or anywhere in the genome based on BLAST analysis, and other closely related genera like *Halobacillus* (e.g., *H. litoralis*) also appear to lack NrdIs. As we do not anticipate that a dimanganese cofactor could be formed without superoxide as an oxidant, these observations suggest a different source of superoxide in these organisms, or that the cofactor requirements of the 2RCC cluster RNRs may vary by organism. Purification and characterization of additional RNRs of this cluster will be necessary to understand their unifying properties and how they relate to the other Mn-dependent (class Ib, Ic, and Id) and NrdI-dependent (class Ib and Ie) RNR subclasses functionally and evolutionarily.

## Supplementary Material



## Data Availability

Materials for the expression of all protein constructs can be provided by J.A.C. pending scientific review and the completion of a material transfer agreement.

## Notes

Experimental section; supplementary results section describing experiments with heterobimetallic cofactors; supplementary tables, including a full description of assembly kinetics data; supplementary figures for biochemical and spectroscopic characterization of dimanganese, diiron, and manganese/iron forms of *Lmo* β; and supplementary references (PDF)

## References

[1] Nordlund P., Reichard P. (2006). Ribonucleotide reductases. Annu.
Rev. Biochem..

[2] Greene B. L., Kang G., Cui C., Bennati M., Nocera D. G., Drennan C. L., Stubbe J. (2020). Ribonucleotide
reductases: Structure,
chemistry, and metabolism suggest new therapeutic targets. Annu. Rev. Biochem..

[3] Stubbe J., van der Donk W. A. (1998). Protein
radicals in enzyme catalysis. Chem. Rev..

[4] Wei Y., Mathies G., Yokoyama K., Chen J., Griffin R. G., Stubbe J. (2014). A chemically competent
thiosulfuranyl radical on the *Escherichia coli* class III ribonucleotide reductase. J. Am.
Chem. Soc..

[5] Kang G., Taguchi A. T., Stubbe J., Drennan C. L. (2020). Structure of a trapped
radical transfer pathway within a ribonucleotide reductase holocomplex. Science.

[6] Westmoreland D. E., Feliciano P. R., Kang G., Cui C., Kim A., Stubbe J., Nocera D. G., Drennan C. L. (2024). 2.6-Å resolution
cryo-EM structure of a class Ia ribonucleotide reductase trapped with
mechanism-based inhibitor N_3_CDP. Proc. Natl. Acad. Sci. U. S. A..

[7] Ando N., Brignole E. J., Zimanyi C. M., Funk M. A., Yokoyama K., Asturias F. J., Stubbe J., Drennan C. L. (2011). Structural interconversions
modulate activity of *Escherichia coli* ribonucleotide reductase. Proc. Natl. Acad.
Sci. U. S. A..

[8] Srinivas V., Lebrette H., Lundin D., Kutin Y., Sahlin M., Lerche M., Eirich J., Branca R. M. M., Cox N., Sjöberg B.-M., Högbom M. (2018). Metal-free ribonucleotide reduction
powered by a DOPA radical in Mycoplasma pathogens. Nature.

[9] Helmann J. D. (2014). Specificity
of metal sensing: Iron and manganese homeostasis in *Bacillus
subtilis*. J. Biol. Chem..

[10] Atkin C. L., Thelander L., Reichard P., Lang G. (1973). Iron and free radical
in ribonucleotide reductase: Exchange of iron and Mössbauer
spectroscopy of the protein B2 subunit of the *Escherichia
coli* enzyme. J. Biol. Chem..

[11] Bollinger J. M., Edmondson D. E., Huynh B. H., Filley J., Norton J. R., Stubbe J. (1991). Mechanism of assembly of the tyrosyl
radical-dinuclear iron cluster cofactor of ribonucleotide reductase. Science.

[12] Cotruvo J. A., Stubbe J. (2012). Metallation
and mismetallation of
iron and manganese proteins in vitro and in vivo: the class I ribonucleotide
reductases as a case study. Metallomics.

[13] Högbom M. (2011). Metal use
in ribonucleotide reductase R2, di-iron, di-manganese and heterodinuclear–an
intricate bioinorganic workaround to use different metals for the
same reaction. Metallomics.

[14] Ruskoski T. B., Boal A. K. (2021). The periodic table
of ribonucleotide reductases. J. Biol. Chem..

[15] Čapek J., Večerek B. (2023). Why is manganese
so valuable to bacterial pathogens?. Front.
Cell. Infect. Microbiol..

[16] Jordan A., Pontis E., Atta M., Krook M., Gibert I., Barbé J., Reichard P. (1994). A second class I ribonucleotide reductase
in Enterobacteriaceae: characterization of the *Salmonella* typhimurium enzyme. Proc. Natl. Acad. Sci.
U. S. A..

[17] Cotruvo J. A., Stubbe J. (2008). NrdI, a flavodoxin
involved in maintenance
of the diferric-tyrosyl radical cofactor in *Escherichia
coli* class Ib ribonucleotide reductase. Proc. Natl. Acad. Sci. U. S. A..

[18] Cotruvo J. A., Stubbe J. (2010). An active
dimanganese­(III)-tyrosyl
radical cofactor in *Escherichia coli* class Ib ribonucleotide reductase. Biochemistry.

[19] Cotruvo J.
A., Stich T. A., Britt R. D., Stubbe J. (2013). Mechanism
of assembly of the dimanganese-tyrosyl radical cofactor of class Ib
ribonucleotide reductase: Enzymatic generation of superoxide is required
for tyrosine oxidation via a Mn­(III)­Mn­(IV) intermediate. J. Am. Chem. Soc..

[20] Boal A. K., Cotruvo J. A., Stubbe J., Rosenzweig A. C. (2010). Structural
basis for activation of class Ib ribonucleotide reductase. Science.

[21] Cox N., Ogata H., Stolle P., Reijerse E., Auling G., Lubitz W. (2010). A tyrosyl–dimanganese
coupled spin system is
the native metalloradical cofactor of the R2F subunit of the ribonucleotide
reductase of *Corynebacterium ammoniagenes*. J. Am. Chem. Soc..

[22] Cotruvo J. A., Stubbe J. (2011). *Escherichia coli* class Ib ribonucleotide reductase
contains a dimanganese­(III)-tyrosyl
radical cofactor in vivo. Biochemistry.

[23] Martin J. E., Imlay J. A. (2011). The alternative
aerobic ribonucleotide reductase of *Escherichia coli*, NrdEF, is a manganese-dependent
enzyme that enables cell replication during periods of iron starvation. Mol. Microbiol..

[24] Zhang Y., Stubbe J. (2011). *Bacillus subtilis* class Ib ribonucleotide
reductase is a dimanganese­(III)-tyrosyl radical enzyme. Biochemistry.

[25] Makhlynets O., Boal A. K., Rhodes D. L. V., Kitten T., Rosenzweig A. C., Stubbe J. A. (2014). *Streptococcus
sanguinis* class Ib ribonucleotide
reductase: High activity with both iron and manganese cofactors and
structural insights. J. Biol. Chem..

[26] Gra̅ve K., Griese J. J., Berggren G., Bennett M. D., Högbom M. (2020). The *Bacillus anthracis* class Ib ribonucleotide reductase subunit
NrdF intrinsically selects manganese over iron. J. Biol. Inorg. Chem..

[200] Jiang W., Yun D., Saleh L., Barr E. W., Xing G., Hoffart L. M., Maslak M.-A., Krebs C., Bollinger J. M. (2007). A manganese­(IV)/iron­(III) cofactor in Chlamydia trachomatis
ribonucleotide reductase. Science.

[27] Andersson C. S., Högbom M. (2009). A *Mycobacterium tuberculosis* ligand-binding
Mn/Fe protein reveals a new cofactor in a remodeled R2-protein scaffold. Proc. Natl. Acad. Sci. U. S. A..

[28] Manley O. M., Phan H. N., Stewart A. K., Mosley D. A., Xue S., Cha L., Bai H., Lightfoot V. C., Rucker P. A., Collins L. (2022). Self-sacrificial
tyrosine cleavage by an Fe:Mn oxygenase for the
biosynthesis of para-aminobenzoate in *Chlamydia trachomatis*. Proc. Natl. Acad. Sci. U. S. A..

[29] Wooldridge R., Stone S., Pedraza A., Ray W. K., Helm R. F., Allen K. D. (2023). The *Chlamydia
trachomatis*
*p*-aminobenzoate synthase CADD
is a manganese-dependent oxygenase
that uses its own amino acid residues as substrates. FEBS Lett..

[30] Powell M. M., Rao G., Britt R. D., Rittle J. (2023). Enzymatic hydroxylation of aliphatic
C–H bonds by a Mn/Fe cofactor. J. Am.
Chem. Soc..

[31] Rose H. R., Ghosh M. K., Maggiolo A. O., Pollock C. J., Blaesi E. J., Hajj V., Wei Y., Rajakovich L. J., Chang W. C., Han Y. (2018). Structural basis for
superoxide activation of *Flavobacterium johnsoniae* class I ribonucleotide reductase and for radical initiation by its
dimanganese cofactor. Biochemistry.

[32] Rozman
Grinberg I., Lundin D., Hasan M., Crona M., Jonna V. R., Loderer C., Sahlin M., Markova N., Borovok I., Berggren G. (2018). Novel ATP-cone-driven
allosteric regulation of ribonucleotide reductase via the radical-generating
subunit. eLife.

[33] Blaesi E. J., Palowitch G. M., Hu K., Kim A. J., Rose H. R., Alapati R., Lougee M. G., Kim H. J., Taguchi A. T., Tan K. O. (2018). Metal-free class Ie ribonucleotide reductase from pathogens
initiates catalysis with a tyrosinederived dihydroxyphenylalanine
radical. Proc. Natl. Acad. Sci. U. S. A..

[34] Zallot R., Oberg N., Gerlt J. A. (2019). The EFI web resource for genomic
enzymology tools: Leveraging protein, genome, and metagenome databases
to discover novel enzymes and metabolic pathways. Biochemistry.

[35] Shannon P., Markiel A., Ozier O., Baliga N. S., Wang J. T., Ramage D., Amin N., Schwikowski B., Ideker T. (2003). Cytoscape: A software environment for integrated models
of biomolecular interaction networks. Genome
Res..

[36] Rose, H. R. ; Palowitch, G. M. ; Hu, K. ; Gandhi, A. ; Boal, A. K. Structure-function relationships in assembly of the radical-initiating cofactors of class Ia–e ribonucleotide reductases. In Comprehensive Natural Products III; Liu, H.-W. ; Begley, T. P. , Eds.; Elsevier, 2020; pp. 415–441.

[37] Ofer A., Kreft J., Logan D. T., Cohen G., Borovok I., Aharonowitz Y. (2011). Implications of the inability of *Listeria monocytogenes* EGD-e to grow anaerobically due to a deletion in the class III NrdD
ribonucleotide reductase for its use as a model laboratory strain. J. Bacteriol..

[38] Tilney L. G., Portnoy D. A. (1989). Actin filaments
and the growth, movement, and spread
of the intracellular bacterial parasite, *Listeria monocytogenes*. J. Cell Biol..

[39] Vázquez-Boland J. A., Kuhn M., Berche P., Chakraborty T., Domínguez-Bernal G., Goebel W., González-Zorn B., Wehland J., Kreft J. (2001). *Listeria* pathogenesis
and molecular virulence determinants. Clin.
Microbiol. Rev..

[40] Cossart P., Lebreton A. (2014). A trip in the “New Microbiology”
with
the bacterial pathogen *Listeria monocytogenes*. FEBS Lett..

[41] Parker M. J., Zhu X., Stubbe J. (2014). *Bacillus
subtilis* class Ib ribonucleotide
reductase: High activity and dynamic subunit interactions. Biochemistry.

[42] Swaminathan B., Gerner-Smidt P. (2007). The epidemiology of human listeriosis. Microbes Infect..

[43] Conte M. P., Longhi C., Petrone G., Polidoro M., Valenti P., Seganti L. (2000). Modulation of actA
gene expression in *Listeria
monocytogenes* by iron. J. Med. Microbiol..

[44] Murdoch C. C., Skaar E. P. (2022). Nutritional immunity: the battle for nutrient metals
at the host–pathogen interface. Nat.
Rev. Microbiol..

[45] McLaughlin H. P., Bahey-El-Din M., Casey P. G., Hill C., Gahan C. G. M. (2013). A mutant
in the *Listeria monocytogenes* fur-regulated virulence
locus (frvA) induces cellular immunity and confers protection against
listeriosis in mice. J. Med. Microbiol..

[46] Pi H., Patel S. J., Argüello J. M., Helmann J. D. (2016). The *Listeria
monocytogenes* Fur-regulated virulence protein FrvA is an
Fe­(II) efflux P_1B4_-type ATPase. Mol.
Microbiol..

[47] Guan G., Pinochet-Barros A., Gaballa A., Patel S. J., Argüello J. M., Helmann J. D. (2015). PfeT, a P_1B4_-type ATPase, effluxes ferrous
iron and protects *Bacillus subtilis* against iron
intoxication. Mol. Microbiol..

[48] Lofstad M., Gudim I., Hammerstad M., Røhr K., Hersleth H.-P. (2016). Activation of the class Ib ribonucleotide
reductase
by a flavodoxin reductase in *Bacillus cereus*. Biochemistry.

[49] Dassama L. M. K., Boal A. K., Krebs C., Rosenzweig A. C., Bollinger J. M. (2012). Evidence that
the β subunit
of *C*
*hlamydia trachomatis* ribonucleotide
reductase is active with the manganese ion of its manganese­(IV)/iron­(III)
cofactor in site 1. J. Am. Chem. Soc..

[50] Bollinger J. M., Tong W. H., Stubbe J. A., Huynh B. H., Edmondson D. E., Ravi N. (1994). Mechanism of assembly of the tyrosyl
radical-diiron­(III) cofactor of *E. coli* ribonucleotide reductase. 3. Kinetics of the limiting Fe^2+^ reaction by optical, EPR, and Mössbauer spectroscopies. J. Am. Chem. Soc..

[51] Outten C. E., O’Halloran V. T. (2001). Femtomolar
sensitivity of metalloregulatory proteins
controlling zinc homeostasis. Science.

[52] Anjem A., Varghese S., Imlay J. A. (2009). Manganese import is a key element
of the OxyR response to hydrogen peroxide in *Escherichia
coli*. Mol. Microbiol..

[53] Osman D., Martini M. A., Foster A. W., Chen J., Scott A. J. P., Morton R. J., Steed J. W., Lurie-Luke E., Huggins T. G., Lawrence A. D. (2019). Bacterial sensors define
intracellular free energies for correct enzyme metalation. Nat. Chem. Biol..

[54] Park J., Cleary M. B., Li D., Mattocks J. A., Xu J., Wang H., Mukhopadhyay S., Gale E. M., Cotruvo J. A. (2022). A genetically encoded fluorescent sensor for manganese­(II),
engineered from lanmodulin. Proc. Natl. Acad.
Sci. U. S. A..

[55] Torrents E., Westman M., Sahlin M., Sjöberg B.-M. (2006). Ribonucleotide
reductase modularity: Atypical duplication of the ATP-cone domain
in *Pseudomonas aeruginosa*. J. Biol. Chem..

[56] Epel B., Schäfer K.-O., Quentmeier A., Friedrich C., Lubitz W. (2005). Multifrequency EPR
analysis of the dimanganese cluster
of the putative sulfate thiohydrolase SoxB of *Paracoccus pantotrophus*. J. Biol. Inorg. Chem..

[57] Golombek A. P., Hendrich M. P. (2003). Quantitative analysis
of dinuclear manganese­(II) EPR
spectra. J. Magn. Reson..

[58] Khangulov S. V., Barynin V. V., Antonyuk-Barynina S.
V. (1990). Manganese-containing
catalase from *Thermus thermophilus* peroxide-induced
redox transformation of manganese ions in presence of specific inhibitors
of catalase activity. Biochim. Biophys. Acta,
Bioenerg..

[59] Yun D., Krebs C., Gupta G. P., Iwig D. F., Huynh B. H., Bollinger J. M. (2002). Facile electron transfer during formation
of cluster X and kinetic competence of X for tyrosyl radical production
in protein R2 of ribonucleotide reductase from mouse. Biochemistry.

[60] Jiang W., Hoffart L. M., Krebs C., Bollinger J. M. (2007). A manganese­(IV)/iron­(IV) intermediate in assembly of
the manganese­(IV)/iron­(III) cofactor of *Chlamydia trachomatis* ribonucleotide reductase. Biochemistry.

[61] Jones G. S., D’Orazio S. E. F. (2013). *Listeria monocytogenes*: Cultivation and laboratory maintenance. Curr.
Protoc. Microbiol..

[62] Jayachandran M., Yoon J., Wu J., Cipurko D., Quon J., Makhlynets O. (2021). Mechanistic
studies of the cofactor assembly in class
Ib ribonucleotide reductases and protein affinity for Mn^II^ and Fe^II^. Metallomics.

[63] Murgas C. J., Green S. P., Forney A. K., Korba R. M., An S.-S., Kitten T., Lucas H. R. (2020). Intracellular
metal speciation in *Streptococcus sanguinis* establishes
SsaACB as critical for
redox maintenance. ACS Infect. Dis..

[64] Peduzzi O. M., Palowitch G. M., Gajewski J. P., Hu K., Wheeler A., Laremore T. N., Silletti S., Komives E. A., Allen B. D., Silakov A. (2025). A structurally divergent class Ia ribonucleotide reductase
from a tick-borne pathogen. Biochemistry.

[65] Rozman
Grinberg I., Berglund S., Hasan M., Lundin D., Ho F. M., Magnuson A., Logan D. T., Sjöberg B.-M., Berggren G. (2019). Class Id ribonucleotide reductase utilizes a Mn_2_(IV,III) cofactor and undergoes large conformational changes
on metal loading. J. Biol. Inorg. Chem..

[66] Xu J., Cotruvo J. A. (2020). The *czcD* (NiCo)
riboswitch responds to iron­(II). Biochemistry.

[67] Xu J., Cotruvo J. A. (2022). Reconsidering the *czcD* (NiCo) riboswitch as an iron
riboswitch. ACS
Bio Med. Chem. Au.

[68] Chatterjee S. S., Hossain H., Otten S., Kuenne C., Kuchmina K., Machata S., Domann E., Chakraborty T., Hain T. (2006). Intracellular gene expression profile
of *Listeria monocytogenes*. Infect. Immun..

[69] Alqurashi A., Alfs L., Swann J., Butt J. N., Kelly D. J. (2021). The flavodoxin
FldA activates the class Ia ribonucleotide reductase of *Campylobacter
jejuni*. Mol. Microbiol..

[70] Patel S., Gupta R. S. (2020). A phylogenomic and
comparative genomic framework for
resolving the polyphyly of the genus *Bacillus*: Proposal
for six new genera of *Bacillus* species, *Peribacillus* gen. nov., *Cytobacillus* gen. nov., *Mesobacillus* gen. nov., *Neobacillus* gen. nov., *Metabacillus* gen. nov. and *Alkalihalobacillus* gen. nov. Int. J. Syst. Evol. Microbiol..

[71] Joshi A., Thite S., Karodi P., Joseph N., Lodha T. (2021). *Alkalihalobacterium
elongatum* gen. nov. sp. nov.: An antibiotic-producing bacterium
isolated from Lonar Lake and reclassification of the genus *Alkalihalobacillus* into seven novel genera. Front. Microbiol..

