# Supplementary material for: Kinetically Divergent Dimanganese–Tyrosyl Radical Cofactor Assembly in Listeria monocytogenes Class I Ribonucleotide Reductase
Source: Biochemistry. 2026 Jul 13;65(15):2387–99. doi: 10.1021/acs.biochem.6c00369 (PMC13445590; doi:10.1021/acs.biochem.6c00369)
Supplement: Supplementary file 1 [file bi6c00369_si_001.pdf]

Supporting Information for

Kinetically divergent dimanganese-tyrosyl radical cofactor assembly in *Listeria monocytogenes* class I ribonucleotide reductase

Paoning G. Chong,‡ Saborni Biswas,‡ Chihjung Chen, Joseph A. Cotruvo, Jr.*

Department of Chemistry, The Pennsylvania State University, University Park, Pennsylvania, 16802

*Corresponding author: juc96@psu.edu

This file contains the Experimental Section, Supplementary Results, Supplementary Tables S1-S6, Supplementary Figures S1-S22, and Supplementary References.

Experimental Section Page S2

Supplementary Results Page S13

Supplementary Tables Page S16

Supplementary Figures Page S20

Supplementary References Page S37

EXPERIMENTAL SECTION

General considerations. Chemical reagents were purchased from Sigma Aldrich at the highest purity available, unless otherwise stated. Sodium phosphate monobasic monohydrate was obtained from Fisher Scientific, MOPS free acid was obtained from Dot Scientific, potassium hexacyanoferrate(III) and kanamycin sulfate were purchased from Research Products International, isopropyl-β-D-thiogalactopyranoside was purchased from Chem-Impex International, adenosine-5'-triphosphate disodium salt hydrate was from Alfa Aesar, Aristar Ultra ultra-pure nitric acid (67-70%) was from VWR, and 37% hydrochloric acid was obtained from Supelco. *E. coli* strains [5-α and BL21(DE3)] for cloning and recombinant protein expression, DNase I, T4 DNA ligase, OneTaq DNA polymerase, and Q5 DNA polymerase were obtained from New England Biolabs. PCR cleanup kits and E.Z.N.A. plasmid DNA mini prep kits were obtained from Omega Bio-tek. GeneJET gel extraction kits were obtained from ThermoFisher. Protein gel electrophoresis was performed on 16% Tris-glycine gels and a Mini Gel tank from ThermoFisher. Automated protein chromatography was run on a GE Healthcare Biosciences Äkta Pure fast protein liquid chromatography (FPLC) system. UV-Visible spectrophotometric measurements were collected on a Cary 60 UV/visible spectrophotometer using a quartz cuvette (Starna Cells) or on a BioTek Synergy H1 microplate reader using Greiner UV-Star 96 well plates (for analyses of chromatography fractions during purification). Quantification of CDP and dCDP for activity measurements used an Agilent G6410B Triple Quad mass spectrometer.

Untagged β in pET-29b(+), N-terminally His_6-tagged α in pET-28a, N-terminally His_6-tagged NrdI in pET-28a, and N-terminally His_6-tagged TrxL in pET-28a were ordered from Twist Bioscience (**Table S5**). The sequence encoding His_6SUMO from pSUMO vector (Nova LifeTech, Inc.) was amplified with the primers SUMO-fwd and SUMO-rev to add 20 bp overlaps to His-α in pET28a(+) and the His_6-SUMO-α construct was created by Gibson assembly using primers SUMOα-fwd1, SUMOα-rev1, SUMOα-fwd2, and SUMOα-rev2 to yield pSUMO-α (**Table S6**). The expression plasmid for ULP1 protease in pET-28a was a gift of Prof. Squire Booker.

Expression and purification of *Lmo* RNR proteins.

His_6SUMO-α. A 100-mL culture of *E. coli* BL21(DE3) transformed with pSUMO-α, grown overnight (16 h), was used to inoculate at 50× dilution two 6 L flasks containing 2 L of LB media and 50 μg/mL kanamycin. Cultures were grown at 37 °C with shaking at 180 rpm until OD_{600nm} =

0.6 was reached, at which point 0.4 mM IPTG was added. The temperature was decreased to 18 °C and the cultures were further incubated for 12 h. Bacterial cultures were centrifuged at 7,000 ×*g* for 7 min at 4 °C and cell paste was collected.

Cell paste was resuspended in 5 mL **Buffer A** (50 mM sodium phosphate, 100 mM NaCl, 10 mM imidazole, 5% glycerol, pH 7.0) per g cell paste, with 5 mM β-mercaptoethanol (BME), 0.5 mM phenylmethanesulfonyl fluoride (PMSF), 1 EDTA-free protease cocktail inhibitor per 50 mL suspension, and 3 μL DNase I per mL suspension added. The suspension was sonicated on a QSonica Q500 at 50% amplitude for 14 min, with a 10 s "on" and 20 s "off" cycle. The solution was then centrifuged at 55,000 ×*g* for 45 min at 4 °C. A Ni-NTA gravity flow column (10 mL) was equilibrated with 15 column volumes (CV) of Buffer A including 5 mM BME and 0.5 mM PMSF. The supernatant fraction from the lysate was loaded onto the column, followed by 30 CV of **Buffer B** (50 mM sodium phosphate, 300 mM NaCl, 30 mM imidazole, 5% glycerol, 5 mM BME, 0.5 mM PMSF, 5% glycerol, pH 7.0). His_6SUMO-α was eluted with 5 CV **Buffer C** (50 mM sodium phosphate, 100 mM NaCl, 250 mM imidazole, 5 mM BME, 0.5 mM PMSF, 5% glycerol, pH 7.0). The eluent was concentrated to <5 mL using an Amicon 30K MWCO filter and loaded onto a Q FF 16/10 column, pre-equilibrated in **Buffer D** (30 mM MOPS, 100 mM NaCl, 5 mM tris(2-carboxyethyl)phosphine (TCEP), 5% glycerol, pH 7.0), on an AKTA system via a 5 mL capillary loop. The sample was injected into the column, which was then washed with 2 CV Buffer D and eluted with a linear gradient of 100-600 mM NaCl at a flow rate of 5.0 mL/min in the same buffer. His_6SUMO-α eluted at 420 mM NaCl and its purity was verified by SDS-PAGE. The purification yielded 2.75 mg per L culture.

Apo-β. A 200-mL culture of *E. coli* BL21(DE3) transformed with pET29b-β, grown overnight (16 h), was used to inoculate at 50× dilution three 6 L flasks containing 2 L of LB media and 50 μg/mL kanamycin. Cultures were grown at 37 °C with shaking at 180 rpm until OD_{600nm} = 0.5 was reached, at which point 100 μM 1,10-phenanthroline was added. After 15 min, 200 μM $MnCl_2$ was added, followed by 0.4 mM IPTG 5 min later. Addition of $MnCl_2$ was important for achieving high yields of full-length protein. The temperature was decreased to 30 °C and the cultures were further incubated for 5-6 h. Bacterial cultures were harvested as described above.

Cell paste was resuspended in 5 mL **Buffer E** (30 mM MOPS, 100 mM KCl, 5% glycerol, 1 mM EDTA, pH 7.0) per g cell paste, with 0.5 mM PMSF, 1 EDTA-free protease cocktail inhibitor

per 50 mL suspension, and 1 µL DNase I per mL suspension added. The suspension was sonicated on a QSonica Q500 at 50% amplitude for 20 min, with a 10 s "on" and 20 s "off" cycle. The solution was then centrifuged at 55,000 ×*g* for 40 min at 4 °C. The supernatant was placed on a stir plate with a stir bar and a solution of 6% streptomycin sulfate was added to a final concentration of 0.8% over the course of 5-10 min, followed by centrifugation at 57,000 ×*g* for 20 min at 4 °C. A Q-Sepharose Fast Flow gravity flow column (20 mL) was equilibrated with 10 CV **Buffer F** (30 mM MOPS, 5% glycerol, 0.5 mM PMSF, 1 mM EDTA, pH 7.0) containing 200 mM NaCl. The supernatant was loaded onto the column, followed by a 3 CV wash with Buffer F containing 200 mM NaCl. The column was eluted with a linear gradient of 200-700 mM NaCl in the same buffer and 5 mL fractions were collected. The fractions were tested on a plate reader and fractions with $A_{260nm}/A_{280nm} < 0.65$ were pooled and concentrated to <5 mL using an Amicon 10K MWCO filtration device. The protein sample was then loaded onto a HiLoad 16/600 Superdex 75 pg column, pre-equilibrated in **Buffer G** (30 mM MOPS, 100 mM KCl, 5% glycerol, pH 7.0), on an AKTA system via a 5 mL capillary loop. The column was eluted with 1.2 CV Buffer G at a flow rate of 1.0 mL/min. Apo-β eluted at 58-72 mL and its purity was verified by SDS-PAGE. ICP-MS of the purified protein showed no result over background for Fe, LOD = 0.932 ppb (0.023 Fe/β2 in a typical 500 µM β2 sample) and 0.015 Mn/β2, 0.036 Zn/β2. The purification yielded 23 mg per L culture.

His₆-NrdI. A 200-mL culture of *E. coli* BL21(DE3) transformed with pET28a-NrdI, grown overnight (16 h), was used to inoculate at 50× dilution three 6 L flasks containing 2 L of LB media and 50 µg/mL kanamycin. Cultures were grown at 37 °C with shaking at 180 rpm until OD_{600nm} = 0.5 was reached, at which point 10 µM riboflavin-5′-phosphate was added, followed 10 min later by addition of 0.4 mM IPTG. The temperature was decreased to 18 °C and the cultures were further incubated for 16 h. Cultures were harvested as described above.

Cell paste was resuspended in 5 mL Buffer A per g cell paste, with 5 mM BME, 0.5 mM PMSF, 1 EDTA-free protease cocktail inhibitor per 50 mL suspension, and 1 µL DNase I per mL suspension added. The suspension was sonicated on a QSonica Q500 at 50% amplitude for 20 min, with a 10 s "on" and 20 s "off" cycle. The solution was then centrifuged at 55,000 ×*g* for 40 min at 4 °C. A Ni-NTA gravity flow column (10 mL) was equilibrated with 15 column volumes (CV) of Buffer A containing 5 mM BME and 0.5 mM PMSF. The supernatant fraction from the lysate

was loaded onto the column, followed by 30 CV of Buffer B. His_6NrdI was eluted with 5 CV Buffer C. The eluent was concentrated to <5 mL using an Amicon 10K MWCO filter. Apoprotein was precipitated in a water bath at 35 °C for 6 min and centrifuged at 18,000 ×*g* for 4 min at 4 °C. The precipitation and centrifugation steps were repeated at the same temperature. The protein sample was then loaded onto a Q FF 16/10 column, pre-equilibrated in Buffer D, on an AKTA system via a 5 mL capillary loop. The sample was injected into the column, which was then washed with 2 CV Buffer D and eluted with a linear gradient of 200-700 mM NaCl at a flow rate of 5.0 mL/min in the same buffer. His_6NrdI eluted at 410 mM NaCl and its purity was verified by SDS-PAGE. The purification yielded 2 mg per L culture.

His_6-TrxL. A 100-mL culture of *E. coli* BL21(DE3) transformed with pET28a-TrxL, grown overnight (16 h), was used to inoculate at 50× dilution two 6 L flasks containing 2 L of LB media and 50 µg/mL kanamycin. Cultures were grown at 37 °C with shaking at 180 rpm until OD_{600nm} = 0.6 was reached, at which point 0.4 mM IPTG was added. The cultures were further incubated for 4 h. Cultures were harvested as described above.

Cell paste was resuspended in 5 mL Buffer A per g cell paste, with 5 mM BME, 0.5 mM PMSF, 1 EDTA-free protease cocktail inhibitor per 50 mL suspension, and 1 µL DNase I per mL suspension added. The suspension was sonicated on a QSonica Q500 at 50% amplitude for 14 min, with a 10 s "on" and 20 s "off" cycle. The solution was then centrifuged at 55,000 ×*g* for 45 min at 4 °C. A Ni-NTA gravity flow column (10 mL) was equilibrated with 15 column volumes (CV) of Buffer A containing 5 mM BME and 0.5 mM PMSF. The supernatant fraction from the lysate was loaded onto the column, followed by 30 CV of Buffer B. His_6TrxL was eluted with 5 CV Buffer C. The eluent was concentrated to <5 mL using an Amicon 3K MWCO filter. The protein sample was then loaded onto a HiLoad 16/600 Superdex 75 pg column, pre-equilibrated in Buffer D, on an AKTA system via a 5 mL capillary loop. The sample was injected into the column, which was eluted with 1.2 CV Buffer D at a flow rate of 1.0 mL/min. His_6TrxL eluted at 79-88 mL and its purity was verified by SDS-PAGE. The purification yielded 11 mg per L culture.

Determination of protein concentrations. Protein concentrations were determined spectrophotometrically on a Cary 60 UV-Vis using molar absorption coefficients (ε_{280nm}) computationally determined by the ExPASy ProtParam tool.[1] The ε_{280nm} values used were 103,600

M^{-1} cm^{-1} for apo-β2 (dimer), 144,510 M^{-1} cm^{-1} for His_6-SUMO-α, and 9,970 M^{-1} cm^{-1} for His_6-TrxL. The concentration of $NrdI_{ox}$ was determined using the extinction coefficient of its riboflavin-5′-phosphate (FMN) cofactor at 454 nm, 12,200 M^{-1} cm^{-1}, which was determined as described.[2]

Determination of Y• concentration by UV-visible spectroscopy. The dropline method[3] was used to estimate Y• concentration spectrophotometrically. For the diferric-Y• cofactor, Eq. 1 was used, whereas for manganese-containing assemblies Eq. 2 was used, with an approximated ε_{409nm} or ε_{410nm} = 3,000 $M^{-1}cm^{-1}$, adapted from *E. coli* class Ib RNR.[4, 5]

$$[Y\bullet] = [A_{409nm} - 2(A_{404nm} + A_{419nm})/3]/\varepsilon_{409nm} \text{ (Eq. 1)}$$

$$[Y\bullet] = [A_{410nm} - (A_{405nm} + A_{415nm})/2]/\varepsilon_{410nm} \text{ (Eq. 2)}$$

Preparation of proteins and reagents for cluster assembly reactions. A Zeba spin desalting column was equilibrated with Buffer G. To remove the TCEP present in the storage buffer, His_6-NrdI (referred to as NrdI subsequently for simplicity) was applied to the column, which was centrifuged at 1,500 ×*g* for 1 min. NrdI and apo-β were placed in 5- or 10-mL pear-shaped flasks and attached to a Schlenk line. Protein samples were cycled through vacuum and argon purges three times on ice and were then brought into an MBraun anaerobic glovebox. Protein samples were cooled at 4 °C for 5 min before centrifugation at 13,000 ×*g* for 2 min to remove any precipitate. NrdI and apo-β concentrations were determined spectrophotometrically. Solutions of ~100 mM $MnCl_2$ and/or $FeSO_4$ and 85 mM $Na_2S_2O_4$ were prepared anaerobically in Buffer G. Oxygen-saturated buffer was prepared by placing Buffer G in a 50-mL round bottom flask. The flask was sealed with a rubber septum, after which a needle connected to a 99.5% UHP O_2 gas cylinder was pushed through septum and O_2 was bubbled into the buffer at 4 °C for 30 min.

Assembly of diferric-Y• β2. In the glovebox, apo-β2 (approx. 300 µM β2, approx. 250 µL) was incubated with 4 equiv. Fe^{II}/β2 and incubated for 15 min at 4 °C. The sample was brought out of the glovebox in an anaerobic cuvette sealed with a septum cap. The cuvette cap was opened and O_2-saturated buffer (approx. 350 µL of 1.1 mM O_2 at 4 °C) was added to initiate the assembly reaction. For diferric assemblies with a gel filtration step, the sample was brought out of the anaerobic glove box and directly injected via a 0.5 mL capillary loop onto a Superdex 200 Increase 10/300 GL column (stored at 4 °C and pre-equilibrated with Buffer G) and eluted with 1.2 CV of

Buffer G at a flow rate of 0.75 mL/min while monitoring absorbances at 280 nm, 260 nm, and 409 nm. As soon as the protein eluted, samples with absorbance at 409 nm were analyzed by UV-visible spectroscopy from 240-800 nm to verify presence of tyrosyl radical and frozen for EPR spectroscopy or used for activity assays. UV-visible spectra were collected over 30 min and ICP-MS samples were prepared.

Reduction of $NrdI_{ox}$ to $NrdI_{hq}$. An anaerobic solution of ~85 mM sodium dithionite was standardized spectrophotometrically against potassium ferricyanide in Buffer G, as described.[2, 6] NrdI was reduced in the anaerobic chamber to the hydroquinone state using 1 equivalent sodium dithionite solution from this standardized solution, and allowing 5 min for full reaction. Spectrophotometric titrations showed that ~1 equiv. dithionite was sufficient to fully reduce $NrdI_{ox}$ to $NrdI_{hq}$ by this procedure, as in previously described NrdIs (**Figure S5**).[2, 4]

Assembly of dimanganese-Y• β2. In the glovebox, apo-β2 (approx. 350 μM β2 and 200 μL) was incubated with 3.4 equiv. Mn^{II}/β2 (4 equiv. Mn^{II}/β2 for initial experiments; 3.4 equiv. minimized mononuclear Mn^{II} signal without needing to add Chelex) and incubated for 15-20 min. $NrdI_{hq}$ (1.5 or 2 equiv. per β2, approx. 210 or 280 μL of 500 μM) was added to β2 and the sample was removed from the glovebox in an anaerobic cuvette sealed with a septum cap. O_2-saturated buffer (approx. 200 μL of 1.1 mM O_2 at 4 °C) was added, upon which the cuvette was immediately inverted to mix, and the UV-visible absorption spectrum was recorded periodically over 60-90 min at 21 °C.

In order to remove some of the unreacted β and therefore increase Y• content, certain samples were injected onto a Superdex 200 Increase 10/300 GL column pre-equilibrated with Buffer G via a 0.5- or 1-mL capillary loop and eluted with 1.2 CV of Buffer G at a flow rate of 0.75 mL/min while monitoring absorbance at the wavelengths 280 nm, 454 nm, and 410 nm. UV-visible spectra from 240-800 nm were collected from fractions with an absorbance peak at 410 nm to verify radical content. Radical-containing fractions were pooled and prepared for ICP-MS, EPR spectroscopy, and activity assays. To quantify the β protein in this sample, a UV-visible spectrum was acquired and the NrdI contribution was removed by spectral subtraction using a reference NrdI spectrum.

Assembly of dimanganese-Y• β2 with addition of SUMO-α, CDP and ATP. A 350 μM β2 solution (~8 μL) was combined with Mn^{II} (3.4 equiv./β2) and $NrdI_{hq}$ (2 equiv./β2; 550 μM, 9.5 μL) in a total volume of 18.5 μL. Meanwhile, in order to remove reducing agent, SUMO-α (100 μL, ~100 μM) was exchanged into Buffer G without TCEP by using a 7K MWCO Zeba Spin desalting column. Subsequently, an 85 μL solution containing 62 μM SUMO-α, 2.8 mM CDP, and 2.8 mM ATP was prepared. O_2-saturated buffer (16 μL, 1.1 mM O_2 at 4 °C) was added to the β reaction and mixed for 10 s to initiate cofactor assembly. Subsequently, the SUMO-α/CDP/ATP solution was added, yielding 120 μL with final concentrations of 22 μM β2, 22 μM SUMO-α2, 2 mM CDP, and 2 mM ATP. The cuvette was immediately inverted to ensure rapid mixing. UV–visible absorption spectra were recorded within <12 s of mixing and monitored periodically over 15 min at 21 °C.

RNR activity assays. The activities of various metalated forms of β2 were assayed in the presence of a 5-fold excess of His_6SUMO-α. Dimanganese-Y• β2, His_6SUMO-α, and His_6-TrxL were thawed on ice. Diferric-Y• was assembled immediately prior to activity assays, without freezing, unless activated during the SEC step. Activity of dimanganese β2 at time points where $Mn^{III}Mn^{IV}$ had not yet converted to Y• was determined by using a freshly assembled dimanganese-Y• β2 cofactor and initiating assays immediately (within 15-20 s) following O_2 addition. The reaction mix consisted of 7.5 μM α2, 1.1 mM cytidine 5′-diphosphate (CDP), 2 mM adenosine adenosine 5'triphosphate (ATP), 5 mM dithiothreitol (DTT), 0 or 10 μM TrxL, and 12.6 mM $MgSO_4$. The total assay volume was 300 μL. After pre-incubation at 21 °C, 1.5 μM β2 was added to initiate the reaction. At time points of 10, 90, 210, and 330 s (15, 60, 120, 180 s for "$Mn^{III}Mn^{IV}$" assays), 70 μL was removed and the reaction was quenched by addition of 7 μL of 2 M formic acid.

Sample work-up was performed as described by Blaesi et al.[7] Quenched reactions were spun through a Nanosep column with a 10K Omega Membrane filter and transferred to an LC/MS vial. The eluate (10 μL) was injected onto an Extend-C18 column (1.8 μm, 4.6 × 50 mm, Agilent Technologies) in 90% solvent B (acetonitrile) and 10% solvent A (25 mM ammonium formate in water, pH 9.0). Compounds were eluted for detection by mass spectrometry by a programmed solvent gradient: 90% B for 2 min; 90-0% B over 8 min; 0% B for 2 min; 0-90% B over 8 min. CDP and dCDP were detected on an Agilent G6410B Triple Quad mass spectrometer operating in

the negative-ion mode (with a dwell time of 100 ms and fragmentor at 135) and quantified via integration of single-ion monitoring (SIM) peaks at *m*/*z* = 402 and 386, respectively.

In initial experiments, we sought to demonstrate whether the presence of the N-terminal SUMO tag affected RNR activity. SUMO-α was incubated with ULP1 protease in a 500:1 molar ratio, respectively, for 1 h at 4 °C, after which removal of the N-terminal SUMO-tag from α was verified by SDS-PAGE. Activity assays were set up as described above with dimanganese-Y• β2, without TrxL, and with SUMO-α or α. The activities of the β protein were similar in assays with SUMO-α versus with α with the SUMO tag removed (201 U/mg with SUMO-α and 168 U/mg with α, n = 1). Therefore, in subsequent experiments, His_6SUMO-α was used as purified, without removal of the SUMO tag.

MnFe-β2 cluster assembly. Apo-β (300 µL, 279 µM β2) was brought into an anaerobic glovebox and incubated with 2 molar equiv. Mn^{II}/β2 for 15 min at 4 °C, followed by addition of a second 2 equiv. Fe^{II}/β2 and a further 15–20 min incubation at 4 °C. The protein was then incubated with 2 equiv. $NrdI_{hq}$/β2 (463 µL, 360 µM), transferred to a septum-capped anaerobic cuvette, removed from the glovebox, and mixed with 300 µL O_2-saturated buffer to a final volume of 1061 µL. The cuvette was inverted several times and UV-visible spectra were recorded over 60 min. Aliquots were prepared for ICP-MS, EPR spectroscopy, and activity assays. The activated EPR sample was prepared by concentrating approximately 500 µL of the reaction mixture described above and treating it with Chelex for 5 min, yielding a final β2 concentration of 124 µM.

MnZn-β2 assembly. To examine Zn^{II}-mediated displacement of Mn^{II} from the dimanganese(II) site of β2, apo-β2 (370 µL, 70 µM in Buffer G) was incubated aerobically with 3.4 molar equiv. $MnCl_2$, followed by addition of 1.6, 1.7, 2.4, or 3.4 equiv. $ZnCl_2$ as described above. Dissociated metal ions were removed by adding Chelex-100 (washed twice in Buffer G, 25% v/v of protein sample, ~100 µL) and nutating for 5 min; control EPR experiments confirmed that this treatment did not alter the dimanganese(II) cluster signal. The sample was transferred to a new 1.7-mL centrifuge tube, avoiding transfer of Chelex-100 beads. Each sample was passed through a 0.5 mL Zeba spin column pre-equilibrated in Buffer G (1,500 ×*g*, 2 min). A Buffer G blank was processed in parallel for ICP-MS. Eluted protein was analyzed by UV-visible spectrophotometry, EPR spectroscopy, and ICP-MS. Cofactor activation was attempted with two samples (3.4 Mn^{II}

followed by 1.7 or 3.4 Zn^{II}), which showed no detectable dimanganese cluster features in their EPR spectra. These samples were then thawed, deoxygenated, and brought into an anaerobic glovebox. Approximately 180 µL of 35 µM β2 was incubated with 2 equiv. $NrdI_{hq}$/β2 and activated following the same protocol described above. The reaction was monitored by UV-visible spectroscopy from 240-800 nm for 60 min, showing no evidence of Y•.

Inductively coupled plasma mass spectrometry (ICP-MS) sample preparation. Protein samples (10-70 µL) were digested in 200 µL aqua regia (1:3 HNO_3 : HCl) for >4 h at 95 °C to evaporate the solution. The solid residuals were resolubilized with 300 µL 70% HNO_3 and incubated in a microtube mixer for 30 min, before diluting to 2.5% HNO_3 with ultrapure water. Samples were analyzed on a Thermo Scientific iCAP RQ ICP-MS instrument at the Penn State College of Earth and Mineral Sciences, Earth and Environmental Systems Institute, Laboratory for Isotopes and Metals in the Environment. A blank sample prepared in parallel with the protein samples was subtracted from each analyte prior to metal quantification.

Continuous-wave (CW) X-band EPR spectroscopy. X-band EPR samples were prepared by transferring 200-250 µL of sample into 4 mm OD/3mm ID clear fused-quartz tubes (SP Wilmad-Labglass) before freezing with liquid N_2. CW EPR measurements were performed on a Freiburg Instruments MS-5000X X-band spectrometer with helium- or nitrogen-flow cryostat and a variable-temperature TeraFlex Bruker resonator. The following standard parameters were used for spectral collection unless otherwise stated: 100 kHz modulation frequency; 0.35 mT modulation amplitude; 81.92 ms conversion time; 5.12 ms time constant; 1 mW power. Spectra were analyzed on ESRStudio (Freiburg Instruments) and simulations were carried out using SpinCount.[8, 9] The software diagonalizes the electronic terms of the spin Hamiltonian: $H = \beta S \cdot g \cdot B + S \cdot D \cdot S + S.A.I - g_n \beta_n B \cdot I + I \cdot P \cdot I$, where the parameters have the usual definitions, and performs least-squares fitting of simulations to the spectra. The quantitative simulations are generated with consideration of all intensity factors, which allows the computation of simulated spectra for a specified sample concentration.

Quantification of the EPR signals was performed by double integration and comparison with standards of known concentrations: 0.12 mM Cu^{II}-EDTA in 5% glycerol was measured at 25 K and 0.94 mM TEMPO in acetonitrile was measured at 150 K using the above parameters using

the SpinCount software. The spin concentration of the sample measured at 77 K was determined by comparison with a Cu(II) standard of known concentration recorded under identical instrumental conditions. To account for the temperature dependence of continuous-wave EPR signal intensity, all double-integrated spectra were normalized according to the Curie-law relationship $I \propto N/T$, as described.[10, 11] The Cu(II) standard spectrum was collected at 25 K, whereas the sample spectrum was collected at 77 K. The spin concentration of the sample was calculated using Eq. 3:

$$C_{\text{samp}} = C_{\text{std}} \left(\frac{I_{\text{samp}}}{I_{\text{std}}}\right) \left(\frac{T_{\text{samp}}}{T_{\text{std}}}\right) \quad \text{(Eq. 3)}$$

where I_{samp} and I_{std} are the double-integrated intensities of the sample and standard, respectively, and T_{samp} = 77 K and T_{std} = 25 K. Quantitative EPR analysis and the use of Cu(II) standards followed established procedures.[12]

Simulations of dimanganese-Y• were performed on the spectra measured at 150 K. As a result of the decoupling from the dimanganese(III) cluster at this temperature, the tyrosyl radical can be simulated as an isolated S = 1/2 and g = (2.026, 2.003, 1.988) species. For the kinetic traces of the formation of Y• and of the decay of $Mn^{III}Mn^{IV}$ monitored by EPR (shown in **Figure 4** and **Figure S20**), all spectra were spin-standardized to ensure accurate quantification (as described above). The formation of Y• was monitored at 150 K by measuring the peak-to-peak amplitude between g ~ (2.019-1.996), while the decay of the $Mn^{III}Mn^{IV}$ signal was tracked at 20 K by monitoring the peak-to-peak amplitude between g ~ (2.323-2.305).

Simulations can be used to assign the hyperfine interactions between the unpaired electron of the tyrosine residue with the four protons present at the 3 and 5 positions (*meta*) of the phenyl ring and the two protons on the β-carbon of the amino acid side chain. Although we were unable to use such simulations in the case of the dimanganese-Y• cofactor for the reasons described in the main text, we could in the case of the diferric-Y• cofactor (**Table S2**). Based on the McConnell relation (Eq. 4), the angle, θ, describing the dihedral angle from the plane normal to the phenyl ring to the β-proton was calculated:

$$A_{iso} = B \cdot \rho\text{C1} \cdot \cos^2\theta \quad \text{(Eq. 4)}$$

The values of B and (ρC1) were fixed as explained for the *B. subtilis* class Ib RNR.[4] The θ value for the diferric cluster-associated Y• was calculated to 50 ± 0.3°, similar to the angle

calculated for *M. tuberculosis* class Ib diferric-Y• β2 (52 ± 6°) and photosystem II (Y_D•) (52 ± 6°).[13, 14]

SUPPLEMENTARY RESULTS

Attempts to characterize the dimanganese-Y• in more detail using EPR spectroscopy

Unfortunately, detailed characterization of the relaxation properties of this Y• proved elusive due to the inability of remove the multiline dimanganese(II) signal from the Y•. Furthermore, the relaxation in this system is very fast, resulting in signals that do not saturate even at 100 mW and 150 K, such that $P_{1/2}$ values (microwave power at half-saturation of the signal) could not be derived. Sharpening of the signal at higher temperatures is due to the decoupling of the Y• from the nearby Mn-based spin-centers. Attempts to specifically probe the dimanganese-Y• cofactor for T_1/T_2 relaxation times by pulsed X-band EPR or to obtain better resolution of hyperfine interactions by CW Q-band EPR were unsuccessful due to interference from underlying dimanganese(II) spectral features. Further attempts to isolate the dimanganese-Y• from the multiline dimanganese(II) signal using differential power saturation at both 10 K and 100 K were unsuccessful, revealing distinct challenges at each temperature (**Figure S16**). At 10 K, power scans aimed at separating the radical from the multiline dimanganese(II) background via signal subtraction failed due to their comparable relaxation properties. When a similar power scan was attempted at 100 K, fast relaxation across the system again prevented the isolation of the radical. At this elevated temperature, the rapid electronic relaxation of the metal ions averages out their magnetic interaction, causing the tyrosyl radical Y• to magnetically decouple from the metal center. However, multiline dimanganese(II) signal exhibited anomalous relaxation behavior at 100 K, increasing in intensity with microwave power rather than undergoing expected saturation or thermal broadening, thus preventing deconvolution of the two signals.

Attempts to assemble heterodinuclear cofactors

The extremely slow Y• formation led us to investigate other possible mechanisms of *Lmo* β activation. Early observations had indicated the ability of the protein to form small amounts of heterodinuclear $Mn^{III}Fe^{III}$ cofactor (**Figure S7**) and to copurify with significant amounts of Zn (**Table S1**, **Figure 1**). Therefore, we probed heterodinuclear cofactor assembly in vitro.

We first tested the possibility of a mononuclear Mn site allowing for Y• formation with the other site filled by a redox-inert Zn^{II} ion. β was loaded first with one metal, then exposed to another, treated with Chelex for 5 min to remove unbound metal ions, and then metal contents evaluated by ICP-MS (**Table S4**). When β was loaded first with 4 Zn^{II}/β2, the Zn ions could not be replaced

by addition of 4 Mn^{II}/β2. However, if 4 Mn^{II}/β2 were added first, ~2 Zn^{II}/β2 could be incorporated. These results suggested that one of the metal-binding sites may be sufficiently selective (or kinetically inert) for Mn^{II} such that it can prevent mismetalation even by Zn^{II}, an ion at the higher end of the Irving-Williams series, whereas the other site is more exchangeable. We leveraged these observations to load a $Mn^{II}Zn^{II}$ cofactor by incubation of apo-β2 with 3.4 Mn^{II}/β2 for 10 min followed by incubation with 1.6, 1.7, 2.4, and 3.4 Zn^{II}/β2 for 10 min, followed by 5 min Chelex treatment and desalting. EPR spectroscopy showed a six-line spectrum indicative of mononuclear Mn^{II} which was nearly identical in intensity for all samples, except for 3.4 Zn^{II}/β2, which resulted in 83% intensity compared to 1.6 Zn^{II}/β2 (**Figure S21A**). An attempt to use $NrdI_{hq}$ and O_2 to activate a $Mn^{II}Zn^{II}$ cluster created using 3.4 Mn^{II}/β2 and 1.7 Zn^{II}/β2 as described above did not produce any Y• observed by UV-visible absorption or by EPR spectroscopy (**Figure S21B**), or quantifiable activity by LC-MS (data not shown). These studies effectively eliminated the possibility of an active mononuclear manganese cofactor and established the strong preference (or kinetic inertness) of one metal site for Mn^{II}.

Using the same strategy that yielded a $Mn^{II}Zn^{II}$ metal cluster, we assembled $Mn^{II}Fe^{II}$ clusters by first loading 3.4 Mn/β2 followed by 1.7 Fe/β2, followed by a 5 min Chelex treatment. Alternatively, samples could also be prepared by adding 1.7 Mn/β2 followed by 1.7 Fe/β2 (as used in **Figure S21C**). Addition of 2 $NrdI_{hq}$/β2 and excess O_2 resulted in protein with activity of 101 ± 6 U/mg β2, enhanced to 310 ± 21 U/mg β2 in the presence of 10 μM TrxL. These proteins contained 1.79 ± 0.21 Mn and 1.36 ± 0.46 Fe per β2 with 0.11 ± 0.06 Y•/β2 determined by EPR (**Table 1**). When assayed with TrxL, the protein activated in the presence of Mn^{II} and Fe^{II} had a specific activity of 2800 U/mg β2 per Y•/β2, ~60% of the activity that would be expected if dimanganese-Y• were responsible for the activity of this protein, and ~5-fold higher than diferric-Y• β2 (**Table 1**). In support of the unlikeliness of diferric-Y• cofactor having been formed, and in line with our previous observations that NrdI is unable to activate diferrous clusters, we observed no evidence of a Y• signal in the EPR spectrum at liquid helium temperatures (**Figure S21C**). This was confirmed by power-dependence studies (**Figure S21D**), as diferric-coupled Y• would be expected to saturate at much lower powers (<0.1 mW) than metal-centered radicals; no evidence of Y• was observed at 10 μW. At higher temperatures (T > 90 K) the signal of a tyrosyl radical started to emerge, sharpening as temperature increased, similar to the dimanganese-assembled cluster. Finally, although partially obscured by the strong $Mn^{II}Mn^{II}$ background signal, the 6-line

feature characteristic of the $Mn^{III}Fe^{III}$ form of the *C. trachomatis* class Ic RNR[15] was also clearly visible at low temperatures, confirming the ability of the system to oxidize $Mn^{II}Fe^{II}$ clusters to at least $Mn^{III}Fe^{III}$ (**Figure S21C**). Therefore, *Lmo* β metalated with Mn^{II} and Fe^{II} can be activated using NrdI and O_2, it forms Y• likely from some amount of $Mn^{II}Mn^{II}$ cluster present but not from any diferrous cluster, it can oxidize $Mn^{II}Fe^{II}$ at least to $Mn^{III}Fe^{III}$, and the cluster has a lower than expected activity given the amount of Y• generated. While this leaves open the possibility than a novel cofactor was generated in addition to dimanganese-Y•, the rather low cofactor yield and complexity of the EPR spectra (in particular, the inability to remove $Mn^{II}Mn^{II}$ signals) unfortunately make it impossible to conclusively demonstrate such a possibility; regardless, its activity would have to be quite low such that it would be unlikely to be relevant.

SUPPLEMENTARY TABLES

Table S1. Metal contents of *Lmo* β expressed and purified under different conditions, as determined by ICP-MS following purification. Values represent mean ± standard deviation for three biological replicates, except 100 µM 1,10-phenanthroline (1,10-phen) and 200 µM Mn^{II}, for which $n = 2$.

Expression conditions	Mn (equiv/β2)	Fe (equiv/β2)	Zn (equiv/β2)
LB	0.15 ± 0.04	1.03 ± 0.18	0.13 ± 0.04
LB + 100 µM Mn^{II}	1.76 ± 0.46	0.40 ± 0.10	0.04 ± 0.01
LB + 500 µM Mn^{II}	2.66 ± 0.77	0.41 ± 0.08	0.02 ± 0.01
LB + 100 µM 1,10-phen + 200 µM Mn^{II}	3.31 ± 0.42	0.07 ± 0.04	0.03 ± 0.06
LB + 100 µM 1,10-phen + 200 µM Mn^{II}, purified in presence of 1 mM EDTA	0.04 ± 0.01	0.05 ± 0.04	0.01 ± 0.01

Table S2. Hyperfine interactions between the unpaired electron of the tyrosyl radical with the neighboring four protons in the diiron-activated cluster.

	Fe^{III}_2-Y•		
**(MHz)**	A_x	A_y	A_z
**β-H1**	53	59	51
**β-H2**	-3	7	-5
**H3**	-17	-5	-20
**H5**	-17	-5	-20

Table S3. Rate constants in s^{-1} and half-lives (min, in parentheses) for intermediate decay and Y• formation in *Lmo* RNR in cofactor assembly experiments under various conditions. Lines 1–3 represent dimanganese assembly replicates performed at 25 °C (data in **Figure 4** are from experiment #2); #4 was carried out identically to #1-3 but at 37 °C (**Figure S19**); and lines 5–7 are replicates of assemblies activated in the presence of SUMO-α, CDP, and ATP at 25 °C (data in **Figure 5** are from experiment #5). Note that, in the cases of #5 and #6, Y• is already partially formed at the shortest time point acquired (12 s). ND: not determined, UV-vis or EPR data not collected for the particular experiment.

	**$Mn^{III}Mn^{IV}$ decay (EPR)**	**Mn^{III}_2-Y• formation (UV-vis)**	**Mn^{III}_2-Y• formation (EPR)**
#1	0.0022 ± 0.0003 (5.2 ± 0.6)	NA	0.0006 ± 0.0002 (18.9 ± 5.9)
#2	0.0016 ± 0.0003 (7.0 ± 1.1)	0.0015 ± 0.0003 (7.6 ± 2.0)	0.0013 ± 0.0004 (8.9 ± 2.8)
#3	0.0010 ± 0.0002 (11.2± 1.8)	0.0010 ± 0.0001 (11.2 ± 1.2)	0.0006 ± 0.0001 (18.5 ± 2.7)
**#1-3 average**	**0.0016 ± 0.0005 (7.8 ± 2.2)**	**0.0013 ± 0.0003 (9.4 ± 1.8)**	**0.0008 ± 0.0005 (15.4 ± 7.1)**
#4 (37°C)	ND	0.0036 ± 0.0004 (3.2 ± 0.6)	ND
#5 (SUMO-α, ATP)	ND	0.0075 ± 0.0006 (1.5 ± 0.1)	ND
#6 (SUMO-α, ATP)	ND	0.0105 ± 0.0005 (1.0 ± 0.1)	ND
**#5-6 average**	**ND**	**0.0090 ± 0.0008 (1.3 ± 0.1)**	**ND**
#7 (SUMO-α, ATP, CDP)	ND	0.0149 ± 0.0017 (0.8 ± 0.1)	ND
#8 (SUMO-α, ATP, CDP)	ND	0.0341 ± 0.0048 (0.3 ± 0.1)	ND
**#7-8 average**	**ND**	**0.0245 ± 0.0051 0.6 ± 0.1**	**ND**

Table S4. Metal contents of *Lmo* β following 20-min incubations with Mn^{II} and Zn^{II}, followed by Chelex treatment to remove unbound metal ions and analysis by ICP-MS. Means ± standard deviations from 2 biological replicates.

**Sample**	**Mn (equiv./β2)**	**Zn (equiv./β2)**
**4 Mn + 2 Zn**	1.91 ± 0.10	2.15 ± 0.15

Table S5. Sequences of codon-optimized *Lmo* RNR genes used for this study. These sequences were inserted into the vectors using NdeI/XhoI restriction sites. In pET28a(+), insertion into the NdeI site adds MGSSHHHHHHSSGLVPRGSH in front of the N-terminal Met encoded by the below sequences. The protein sequence of the full SUMO-α is provided in **Figure S3**.

Gene	Sequence
Untagged β in pET-29b(+)	ATGGCAAACCAAAAGGAGCAGTTAACACGTATTAAGATCCTTGAGCCGTTGTTCCCTAATCGTT<br>CCACCTCCATTATCAATGGCGAGACCTCCGGGATCTTGAACTGGAACGACATCCCCTATCCAAG<br>CTTCTACCGTGCGTACAAAGAACTGTCGACAAACTATTGGATTCCTGATGAGGTAGACATGAAG<br>TCTGACGCGAAACAATATCCAGCGCTGTCAGAACAGGAGAAATATGCTTTCGATGCTATCATCG<br>GACTTTTAGCCACTCTGGATTCACCCCAGACGCGCTTTATCTATAACATCGCGGAATATATTAC<br>AGACCCGGCGGTACATGCGAACGCGGCAATTATTGCTCAGCAAGAGGTCATTCATAATGAGTCA<br>TATTCCTATGTTTTGGCCTCAATCACCAATCTGCAAGAGCAGAACCGCGTCTTTGAGCTGGCAC<br>GTACGCACCCTACTATTATCAAACGTAACGAGCCTATCATGGATGCGTACGACGACTTCATGAA<br>TAACAAAACCGGTGAAACTTTAGTCAAGGCTTTGATCCAAAGTAGCATCCTTGAAGGAATCAAC<br>TTCTATTCAGGCTTTGCTTATTTTTACAACCTGGTTCGTCAAAATAAAATGACAGGCACAGGTA<br>AAATCATCTCCTTTATCAATCGTGACGAGCTGGCTCACTCTAAGTTCATTTCAGAGGTGATTCG<br>CGCAATTTTAGGGGAGAACCCAGAGCTGCAAACAGAGGAATTGGTCGAATACACCCATGAAGCT<br>TTTCGTCATGCCGTGGAACTTGAGACGGAATGGAGCGAGGAAGTTTTACAAGGCATCGAAGGCA<br>TCGACGTGGAGGAAATGGTAGATTACGTCAAGTACCGTGCCAATAAGATGTTGGGCATGTTGGG<br>AATCCCTGAGCTTTACCCTGGACACAGCGACAACACAATGACGTGGATTAAGGCCTACGCTGAC<br>AACTTCACCGAAACTAAGACTGATTTTTTCGAAATGCGTAATTCTTCATACAAAAAGACGAATA<br>TGGATAATGGGTTCGATGATCTGTAA
His$_6$NrdI in pET-28a(+)	ATGCGTATTTTGTTAGCCTATGACTCGCTGTCGGGAAACACCAAAATGGTGGCAGATGAAATTG<br>AGGAGATTCTGATTTCAGAGGGTCACGAGGTTGTATCATTTCGCGTGTCCCCTTTGGCGGAATA<br>CCCACTGGATGAAGATTTCGACCTTTATGTGTTAGGGGCATGGACTGTCGATTACGGCCGCACA<br>CCACCCGACATGAAGGACTTCATTGCGGAACTTGCGGTGAAGCCTAAGAACGTGGCCATCTTCG<br>GTACGGGAGAGACTCAGTGGGGTATGGACTTCTACTGTGGCGCGGTTGATCGTATGGCCAAGTA<br>TTTCGGTACTAGTTACCCGACTCTGAAGATCGAGCAGATGCCTCATACGGAACAGGACGTAGCT<br>GACATTGATAACTGGGTGCGTAAGATCCTTGCGCTGCGCTCCGGCATCAAATAA
His$_6$TrxL in pET-28a(+)	ATGACTTCGATTGAAATTAAATCGCCGGAAGAATTTCAAGCACATATCAACGGGGAGGAGCTGG<br>TGTATGTTGATTATTGGAAAGACAATTGCCCAAATTGTAAGATGCTGGACTTGTCGTTCGCAGA<br>GTTTAAGAATAGCGAAATCGCTTCGAAAGTAAAGGTTCTGAAAGTAAAATTGGAAGAAATGGGA<br>GAGAACTTCTTTTTCGACCGTGATGTTCAGCAAACTCCGACATTAGTTTTATATAAGGGGGGTG<br>AAGAGATTCATCGTCTTAACGGATTCATCCCACCTAACAAAATTGAAGAGGCAGTCTCGCTTAA<br>TGCATAA
His$_6$α in pET-28a(+)	ATGAAATGGGGTCTGCAAATGACCACTTATATTGTGAAAGATGGGGGAAACCGCAAGTTGCCAT<br>TTGATAAATCACGCTTGGATGGATACCTTGAAAAGATTCATGAGGAGTTTCCGAAGCTTGATTT<br>GGAGGATTACAAACGCAAGGTTTTCAACTTTGTGGAAAAAAAAGAGGACTACGCGGCGGATGAG<br>TTGGTAGACTACTTGATTCGTGAGGCGGAGGCACGCACAGATATCCACATTCCTGAGTGGGAGC<br>ATTTCGCCGCGCGCTTATACTTAAACAAACTTTATAAGAAAGCCAGCAAAAACCGCTTTTACAA<br>CGACGACGATAAATACGGAAGCTACGTCGGACTGCAGGAATCCCTGGGGGAGCGCGGGATCTAC<br>TCAGGAAATATTCTGAAAAATTATTCCAAAGAGGACCTTATTGCTGCTGGGAAACTGATTGACC<br>CAGAAAAAGACAAGTTATTCACGTACAACGGACTGTATCTGCTTGCGACCCGTTATTTAGCCAC<br>AGATTCGGAACGCAATGTGTACGAGTTGCCACAGGAACGTTGGCTTACTATTGCTCTTTACCTG<br>ATGCAGAATGAGCCGAAAGAGAAGCGTATGAAGTTAGTAGAGGAGGCGTACTGGGCTTTATCGA<br>ATCTTTACATGACTGTTGCCACTCCTACGTTGGCAAATGCAGGGAAGGTAGGGGGCCAGCTGAG<br>CTCTTGCTTCATCGACACCGTTGACGATTCCTTACAAGGCATCTACGATTCAAATACGGATGTT<br>GCACGCGTCTCGAAACATGGTGGAGGGGTCGGCGCGTACCTGGGTTATGTACGCTCGACCGGTG<br>CGGCAATTCGTGGTGTTAAGGGTGCCTCGGGGGGCGTAATTCCCTGGATTAAGCAGCTTAACAA<br>CACCGCGGTATCTGTTGACCAGTTGGGTCAGCGTAAGGGTGCTATTGCCGTCTACTTGGATGTC

	TTCCATAAGGACATCGAATCCTTTTTGGATCTGCGCTTGAACAACGGCGACCAACGCTTACGTG<br>CTCACGATGTATTCACCGCGGTGTGCATTCCTGACATCTTCATGGAGGCTGTCGAACGCCGCGG<br>AGAGTGGTACCTTTTCGACCCGCACGAGGTTAAAGCTAAGAAAGGGTGGTATCTGCAAGATTTC<br>TACGACGAATCCAAGGGCGAAGGGACCTTCCGCGAAAAATACGAAGAACTTGTCGCGGATGAAA<br>CGATCTCTAAGAAGATCGTGAAAGCAATTGATATTATGAAGCGCATTATGATGTCTCAACTGGA<br>GACGGGGAACCCATTCATGTTCTATCGTGACGAAGTCAATCGTATGAATCCCAATAAACACGAA<br>GGGATGGTTTACTCCTCGAATCTGTGTACGGAAATCATGCAGAATATGTCGCCGACTAAAATGA<br>TTCAAGAGATCATTTCGGGAGACCAGATTGTTATCACGAAGCAGGCGGGAGACTTTGTCGTATG<br>CAACTTGTCTAGCGTTAATTTAGGACGTGCAGTTGTAGCAGAGGAAGGTACTCTGGAACGCTTG<br>ATTGAAGTTGAAGTACGTATGTTAGACAATGTCATCGATTTGAACGAGTTACCCGTGCCACAGG<br>CCACTATTACTAACCAGAAGTATCGTAGCATTGGGTTAGGGACTTTTGGGTGGCACCACCTGCT<br>GGCGCTTAAAAACATTGCGTGGGATTCGGAAGAGGCGGAGAAATATGCAGACGAGCTTTATGAG<br>CAGATTAATTACCTTGCAATCCGCGCCTCAAATAAATTAGCACAGGAGAAGGGAGCCTACAAGG<br>TATTTAAAGGTTCCGATTGGAACACTGGAGAATATTTCGCACGTCGCAATTACAATAGCCCCGA<br>GTGGCAAGAGTTGGCAAAGGAGGTGGCGGAAAAAGGGTTGCGCAATGCCTACCTGGTTGCGGTC<br>GCACCTAACATGTCCACAGCCCAGATTGCTGGATCCACAGCCTCGATTGATCCAATTTATTCAG<br>CATTCTACTACGAGGAGAAGAAAGACTATCGTCGTCCGGTGATTGCACCCGACCTTAATCTTAG<br>CACTTACCCGTACTACGAAAAAGGAGCCTACAAGGTTGATCAATTCGCGTCCGTTCGTCAGAAC<br>GGTCGCCGCCAACGCCACGTAGATCAATCACTTTCATTTAACTTTTATGTTCCCTCGGCATTA<br>AGGCATCCAAGCTGTTGGAATTGCACATGACTGCTTGGAACGAGGGGCTTAAGACGACCTATTA<br>CGTCCGTTCTAATGACATTGACGTCGAAGAATGCGAATGGTGCTCATCCTAA

Table S6. Primers used for cloning the His$_6$-SUMOα construct.

Primer	Sequence
**SUMO-fwd**	5′-TTAACTTTAATGGGTCATCACCATCATCATC-3′
**SUMO-rev**	5′-TCACCGCGAACAGATTGGAGGTATGAAATGGGGTCTGCAAATGACC-3′
**SUMOα-fwd1**	5′-GTTTAACTTTAAGAAGGAGAT-3′
**SUMOα-rev1**	5′-TTCCTTTCGGGCTTT-3′
**SUMOα-fwd2**	5′-CTAACAAAGCCCGAAAG-3′
**SUMOα-rev2**	5′-ATCTCCTTCTTAAAGTTAAAC-3′

SUPPLEMENTARY FIGURES

Figure S1. Organization of the *Lmo* class I RNR gene cluster, drawn to scale. The present work indicates that the genes encoding α and β, *nrdA* and *nrdB*, respectively, should be renamed as *nrdE* and *nrdF*, according to the convention for class Ib RNRs.

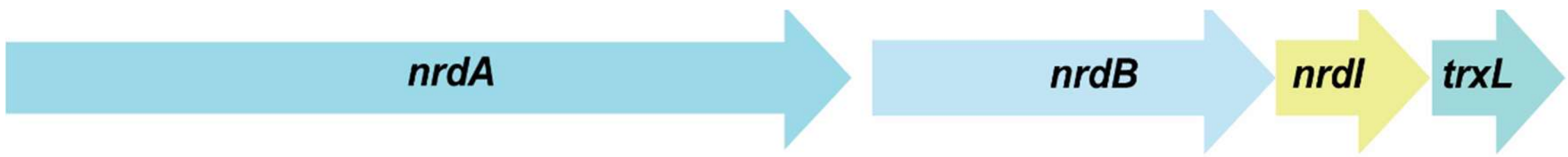

Figure S2. SDS–PAGE (16% Tris-Glycine) analysis of purified *Lmo* class I RNR proteins (15 μg of each protein was loaded). Lane 1: molecular weight ladder. Lanes 2-5: SUMO-α, β, His_6NrdI, and His_6TrxL.

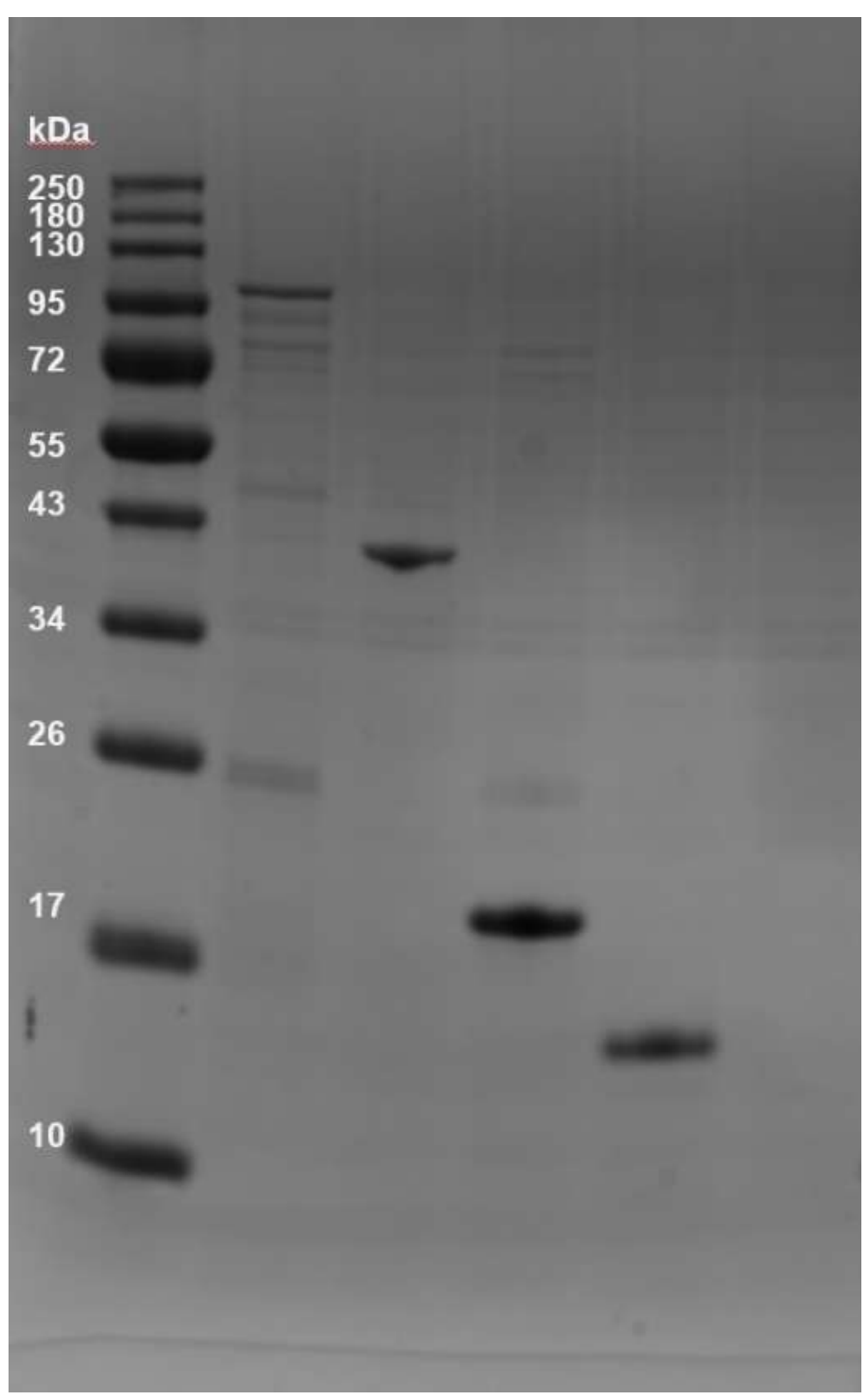

Figure S3. Mass spectrometry characterization of full-length SUMO-α. **(A)** An intact protein MALDI-TOF mass spectrum of *Lmo* SUMO-α, with expected mass of 99524.47 Da (no N-terminal Met) as calculated by the ExPASy ProtParam tool. The $[M + H]^+$ ion with m/z = 99382.445 is 143 Da less than the expected MW. However, the $[M+2H]^{2+}$ ion with m/z = 49,739 and the $[M+3H]^{3+}$ ion with m/z = 33161 both correspond to 99,474 Da, which lies just within the 500 ppm instrument error range (±50 Da in a 100-kDa protein) of the expected MW. Signals (2) and (3) are unknown impurities. **(B)** Predicted amino acid sequence of full-length SUMO-α. The His_6-SUMO tag is underlined.

A

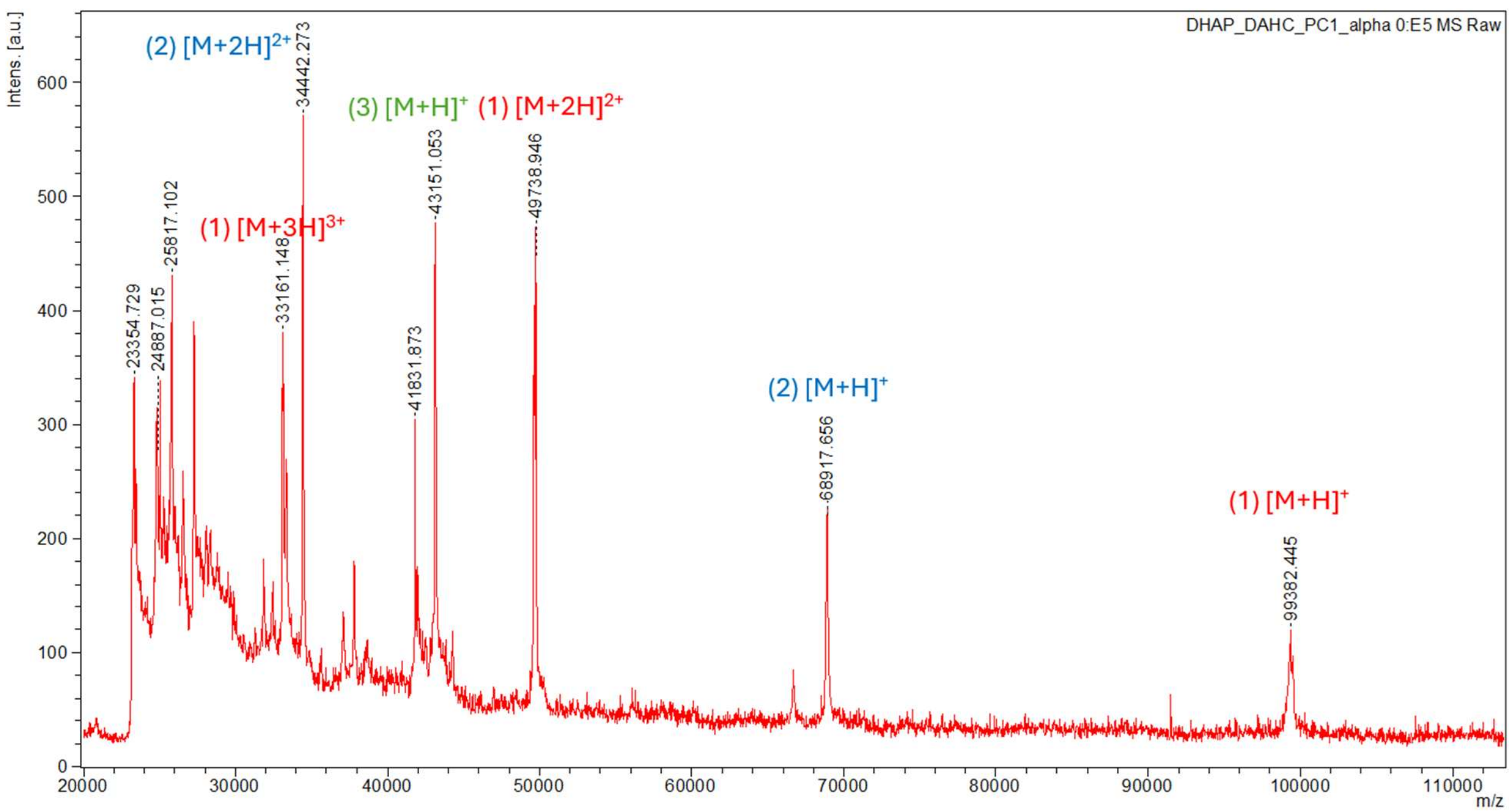

B

```
GHHHHHHGSLQDSEVNQEAKPEVKPEVKPETHINLKVSDGSSEIFFKIKKTTPLRRLMEAFAKRQGKEMDSLRFLYD
GIRIQADQAPEDLDMEDNDIIEAHREQIGGKWGLQMTTYIVKDGGNRKLPFDKSRLDGYLEKIHEEFPKLDLEDYKR
KVFNFVEKKEDYAADELVDYLIREAEARTDIHIPEWEHFAARLYLNKLYKKASKNRFYNDDDKYGSYVGLQESLGER
GIYSGNILKNYSKEDLIAAGKLIDPEKDKLFTYNGLYLLATRYLATDSERNVYELPQERWLTIALYLMQNEPKEKRM
KLVEEAYWALSNLYMTVATPTLANAGKVGGQLSSCFIDTVDDSLQGIYDSNTDVARVSKHGGGVGAYLGYVRSTGAA
IRGVKGASGGVIPWIKQLNNTAVSVDQLGQRKGAIAVYLDVFHKDIESFLDLRLNNGDQRLRAHDVFTAVCIPDIFM
EAVERRGEWYLFDPHEVKAKKGWYLQDFYDESKGEGTFREKYEELVADETISKKIVKAIDIMKRIMMSQLETGNPFM
FYRDEVNRMNPNKHEGMVYSSNLCTEIMQNMSPTKMIQEIISGDQIVITKQAGDFVVCNLSSVNLGRAVVAEEGTLE
RLIEVEVRMLDNVIDLNELPVPQATITNQKYRSIGLGTFGWHHLLALKNIAWDSEEAEKYADELYEQINYLAIRASN
KLAQEKGAYKVFKGSDWNTGEYFARRNYNSPEWQELAKEVAEKGLRNAYLVAVAPNMSTAQIAGSTASIDPIYSAFY
YEEKKDYRRPVIAPDLNLSTYPYYEKGAYKVDQFASVRQNGRRQRHVDQSLSFNFYVPSGIKASKLLELHMTAWNEG
LKTTYYVRSNDIDVEECEWCSS
```

Figure S4. UV-visible spectra of NrdI before and after heat precipitation of apo-NrdI. The spectrum for untreated NrdI was normalized according to A_{454}. A_{280}/A_{454} decreased from 8.5 to 6.0 after heat treatment for this sample.

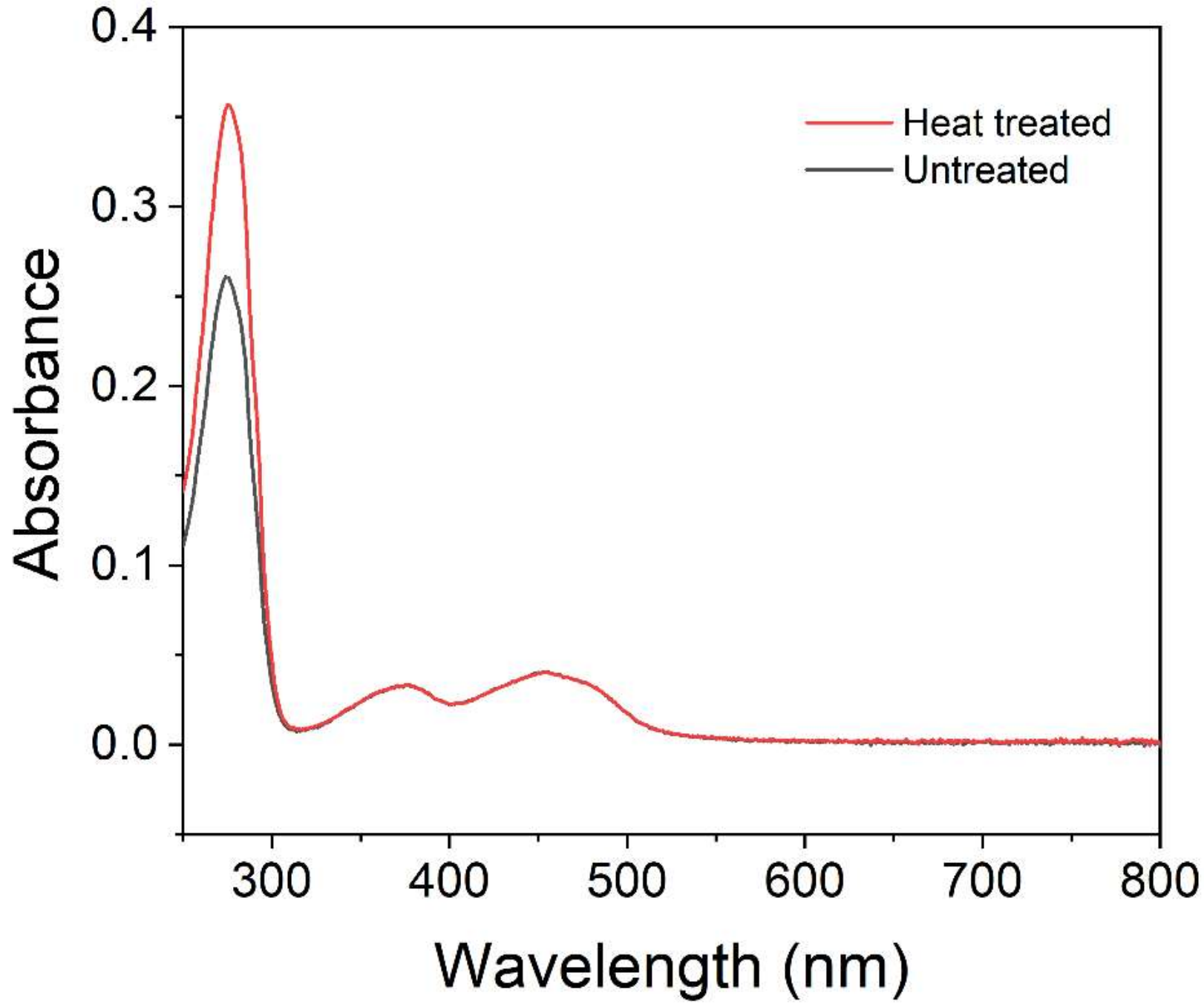

Figure S5. UV-visible spectra of 20 μM NrdI titrated with sodium dithionite showing approximately stoichiometric reduction via a blue semiquinone as with previously characterized NrdI proteins.[2]

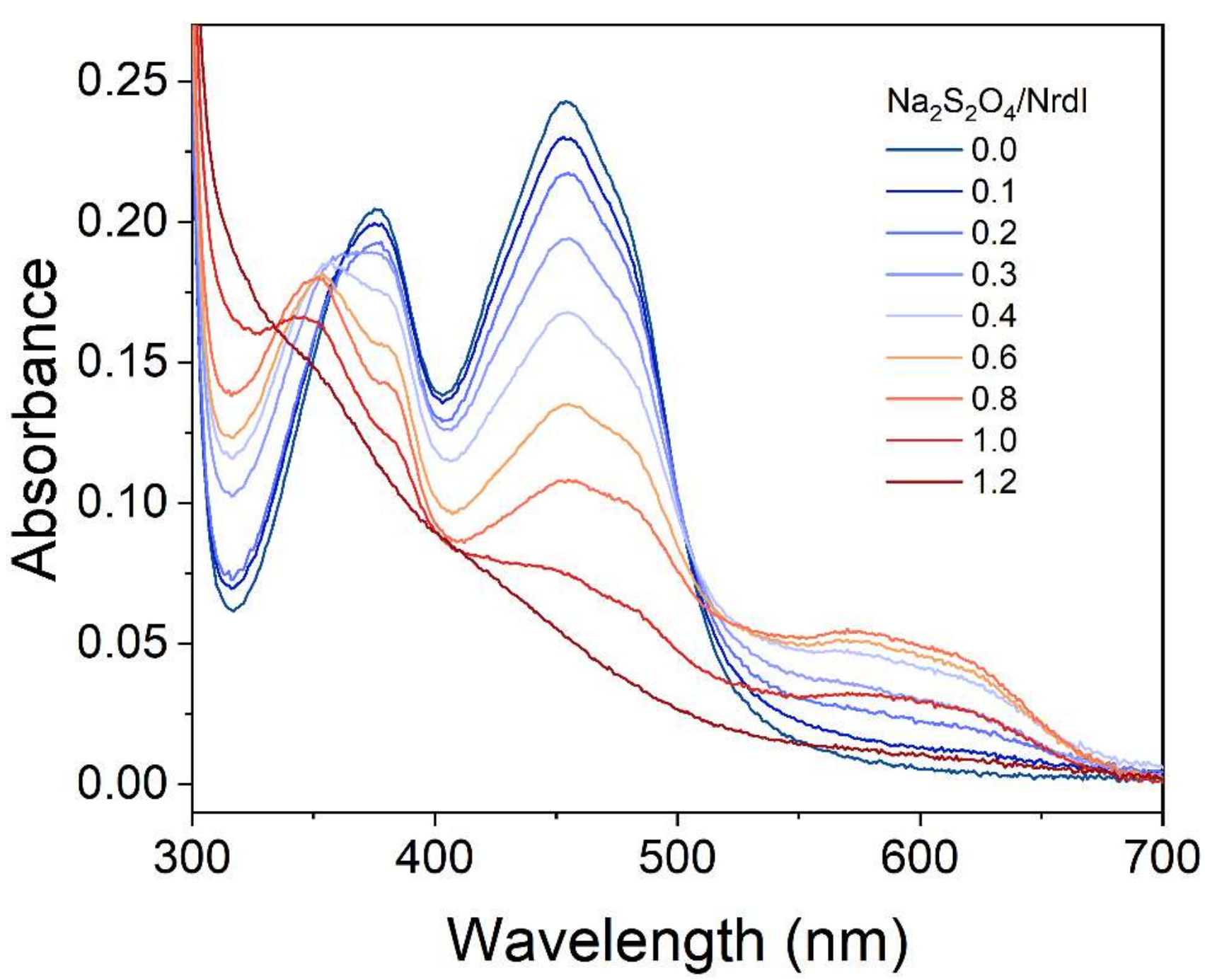

Figure S6. Mass spectrometry characterization of apo-β. **(A)** An intact protein MALDI-TOF mass spectrum of *Lmo* apo-β, with expected mass of 40,086.03 Da (no N-terminal Met) as calculated by the ExPASy ProtParam tool. The *m/z* of the $[M+H]^+$ ion, 40037, is 50 less than expected, which is beyond the instrument error (~20 Da in a 40 kDa protein). The impurity at 43157 Da is unknown. **(B)** The ESI mass spectrum is an exact match to the expected molecular weight (e.g., 1542.7354 peak corresponds to $z = 26$). **(C)** Predicted amino acid sequence of full-length apo-β.

A

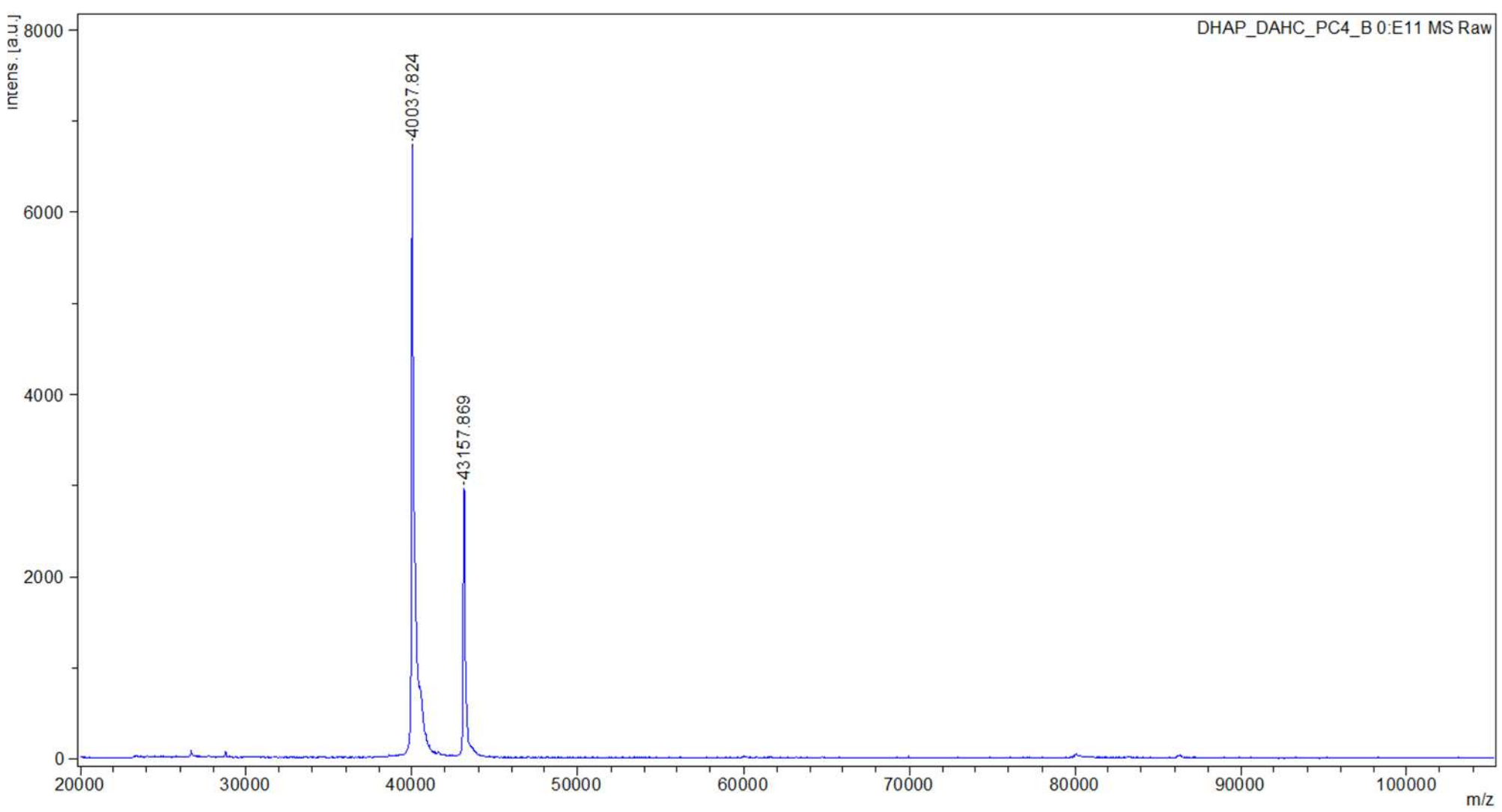

B

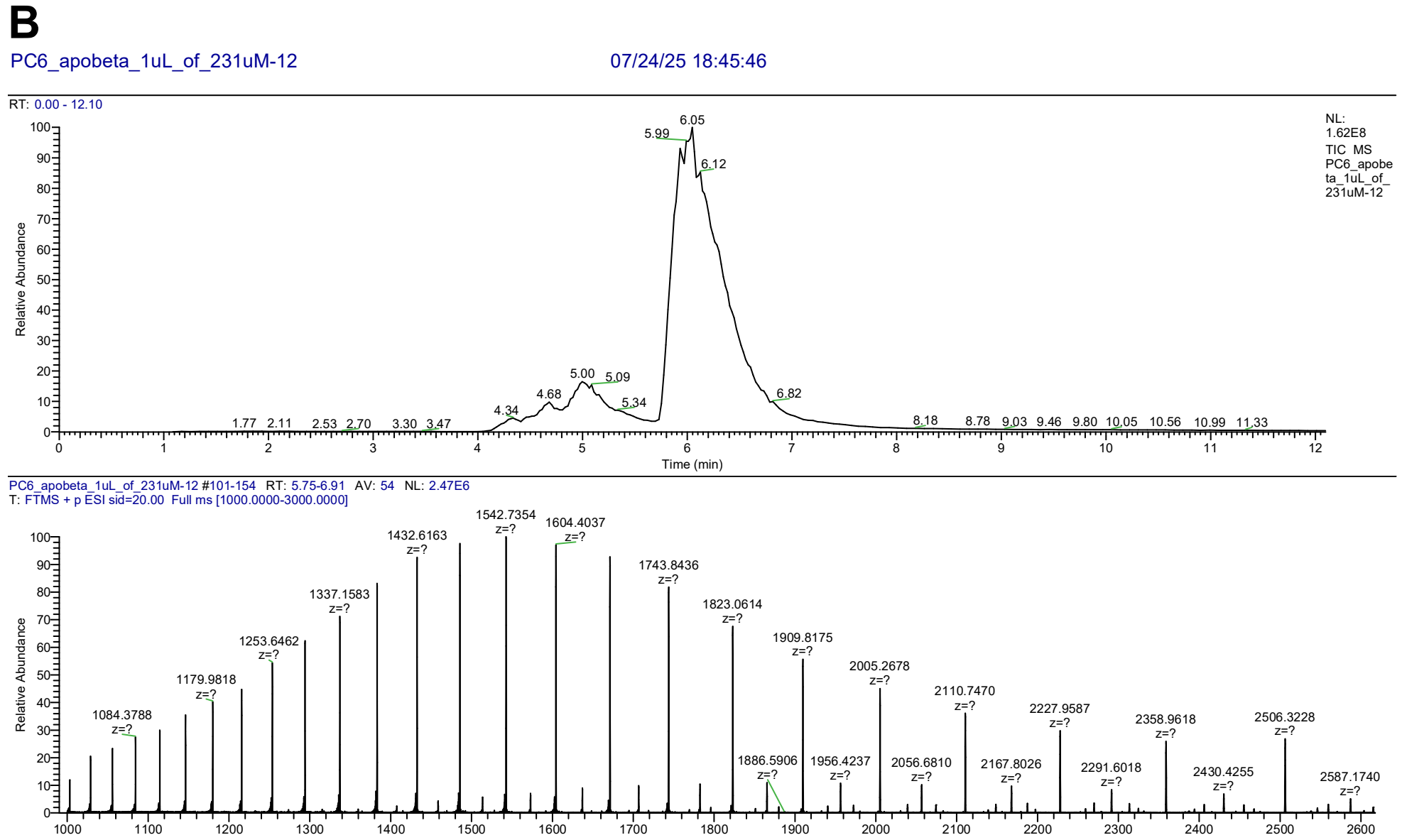

C

```
ANQKEQLTRIKILEPLFPNRSTSIINGETSGILNWNDIPYPSFYRAYKELSTNYWIPDEVDMKSDAKQYPALSEQEK
YAFDAIIGLLATLDSPQTRFIYNIAEYITDPAVHANAAIIAQQEVIHNESYSYVLASITNLQEQNRVFELARTHPTI
IKRNEPIMDAYDDFMNNKTGETLVKALIQSSILEGINFYSGFAYFYNLVRQNKMTGTGKIISFINRDELAHSKFISE
VIRAILGENPELQTEELVEYTHEAFRHAVELETEWSEEVLQGIEGIDVEEMVDYVKYRANKMLGMLGIPELYPGHSD
NTMTWIKAYADNFTETKTDFFEMRNSSYKKTNMDNGFDDL
```

Figure S7. (A) UV-visible spectrum of β (722 µM β2) as purified from *E. coli* grown in LB (see **Table S1** for metal contents). **(B)** X-band EPR spectrum of the same protein, collected at 77 K with 1 mW power and 0.35 mT modulation amplitude, showing the Y• signal at g = 2.005 with simulation (black). The spectrum was simulated using S = 1/2 and g = [2.026, 2.003, 1.988]. See also **Table S2**. **(C)** Full scan EPR spectrum collected at 20 K. **(D)** Enlargement of the putative $Mn^{III}Fe^{III}$ signal. Simulation of $Mn^{III}Fe^{III}$ (black) approximates to 0.03 $Mn^{III}Fe^{III}$/β2. Simulations used a g-tensor of the S = 1/2 ground state [2.045, 2.044, 2.002] and a ^{55}Mn hyperfine coupling tensor, A_{Mn}, of [276, 346, 301] MHz, similar to *Chlamydia trachomatis* class Ic RNR.[16, 17] **(E)** Simulation for g ~ 4.3 "junk" iron signal with parameters (g ~ 2, D = 7.0, E/D = 0.33).[18]

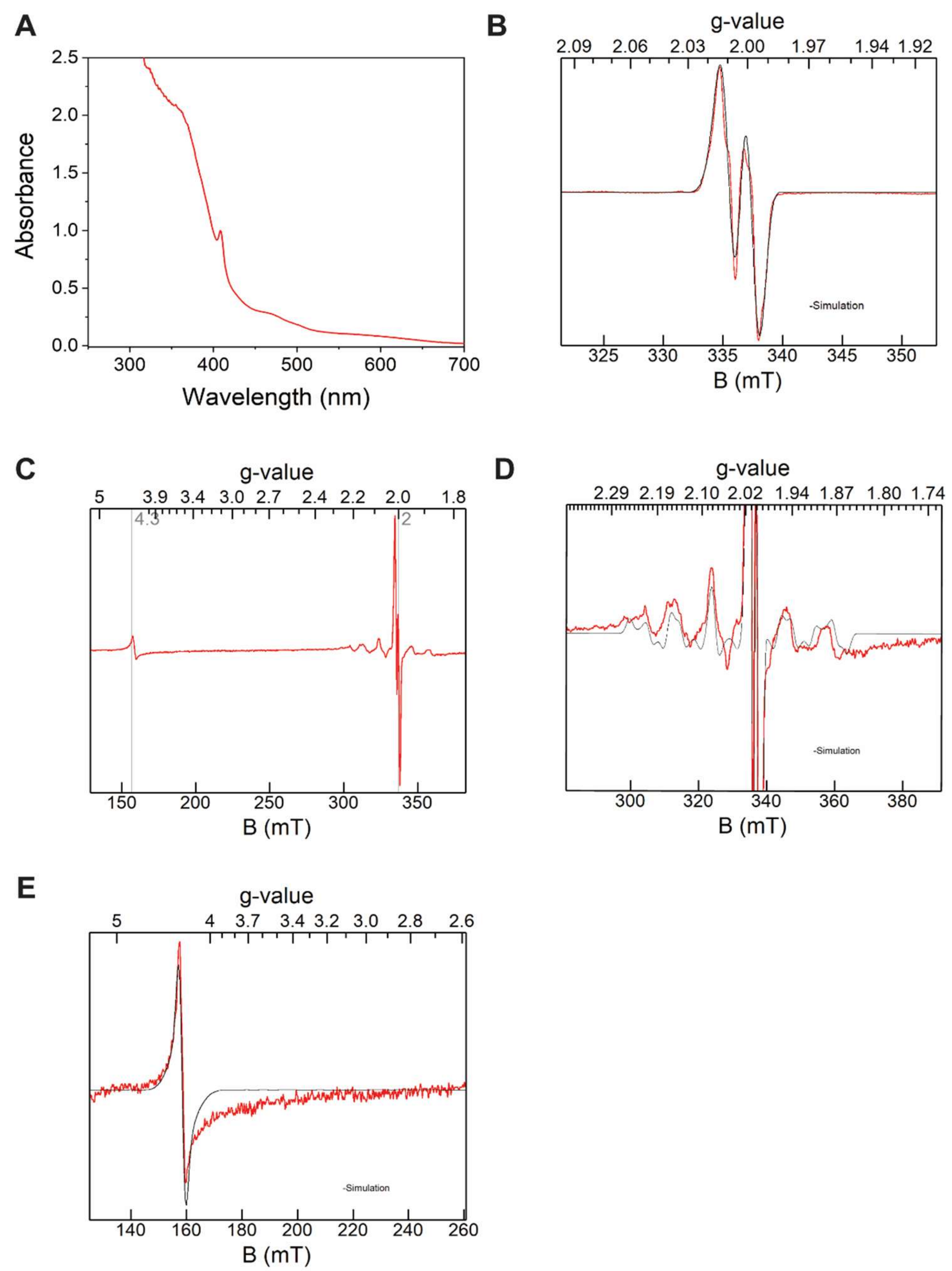

Figure S8. dCDP production determined by LC/MS analysis of reactions of β2 **(A)** as purified, **(B)** after diferric-Y• assembly and S200 chromatography, **(C)** after dimanganese-Y• assembly, and **(D)** after dimanganese-Y• assembly and S200 chromatography, when assayed with 5-fold excess of SUMO-α2 and with DTT, and in the presence of TrxL (+) or absence of TrxL (–). The slopes of the lines of best fit were used to calculated the rate of dCDP formation and used to calculate the specific activities reported in **Table 1**.

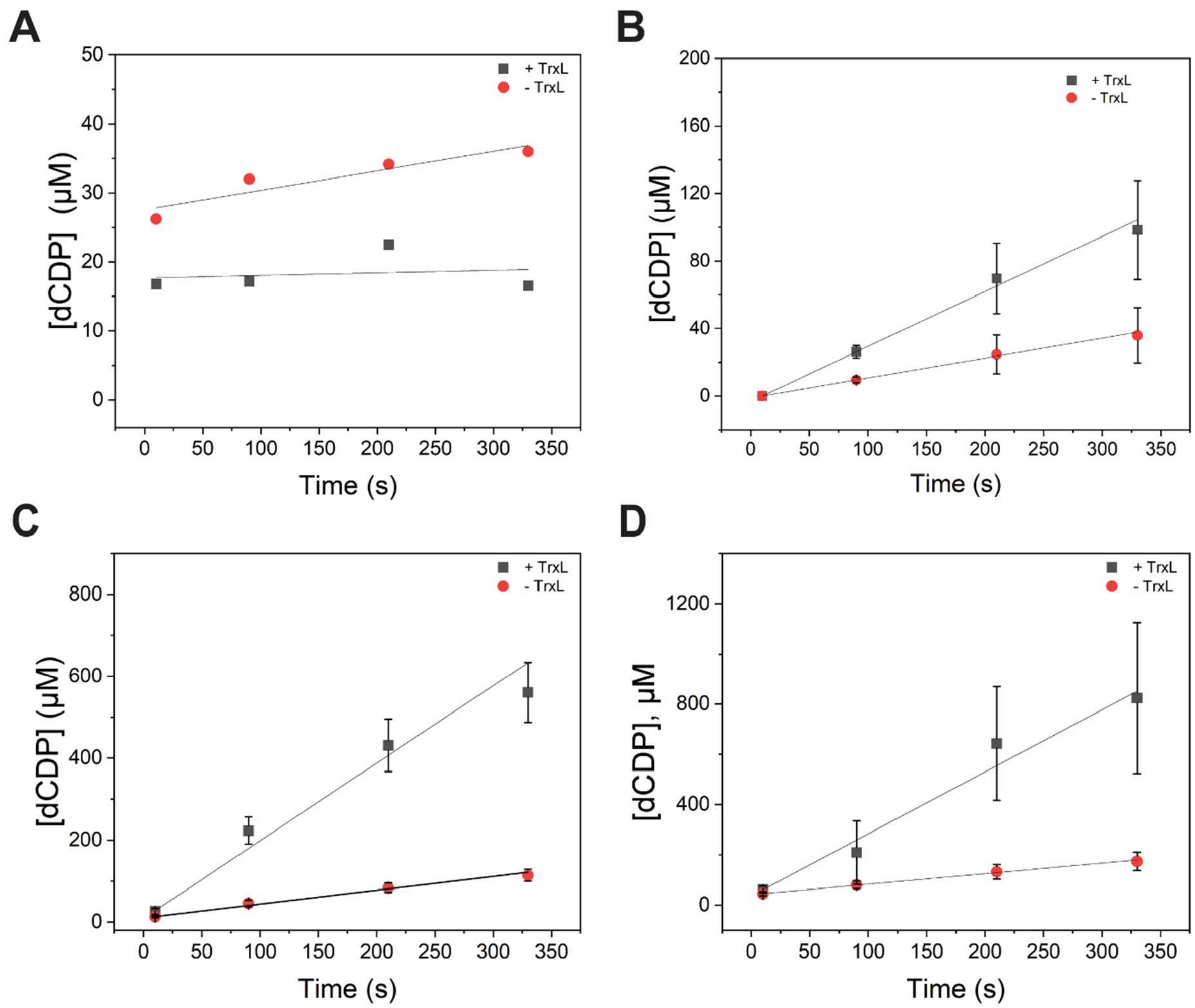

Figure S9. (A) UV-visible spectra showing diferric-Y• decay over time and **(B)** Y•/β2 as calculated by the dropline correction method. Diferrous-β2 (54 μM) was reacted with excess O_2 at 25 °C. The Y•/β2 content immediately after assembly was 0.54 by EPR and 0.46 by the UV-visible dropline method, in reasonable agreement (~15% difference). **(C)** X-band EPR spectra of the samples from (A) taken at t = 2 min and t = 122 min following O_2 addition. After 122 nm, only 26% of Y• content remains. **(D)** UV-visible spectrum showing diferric-Y• formation at 37 °C with stoichiometric amount of O_2 and measured at t = 30 min.

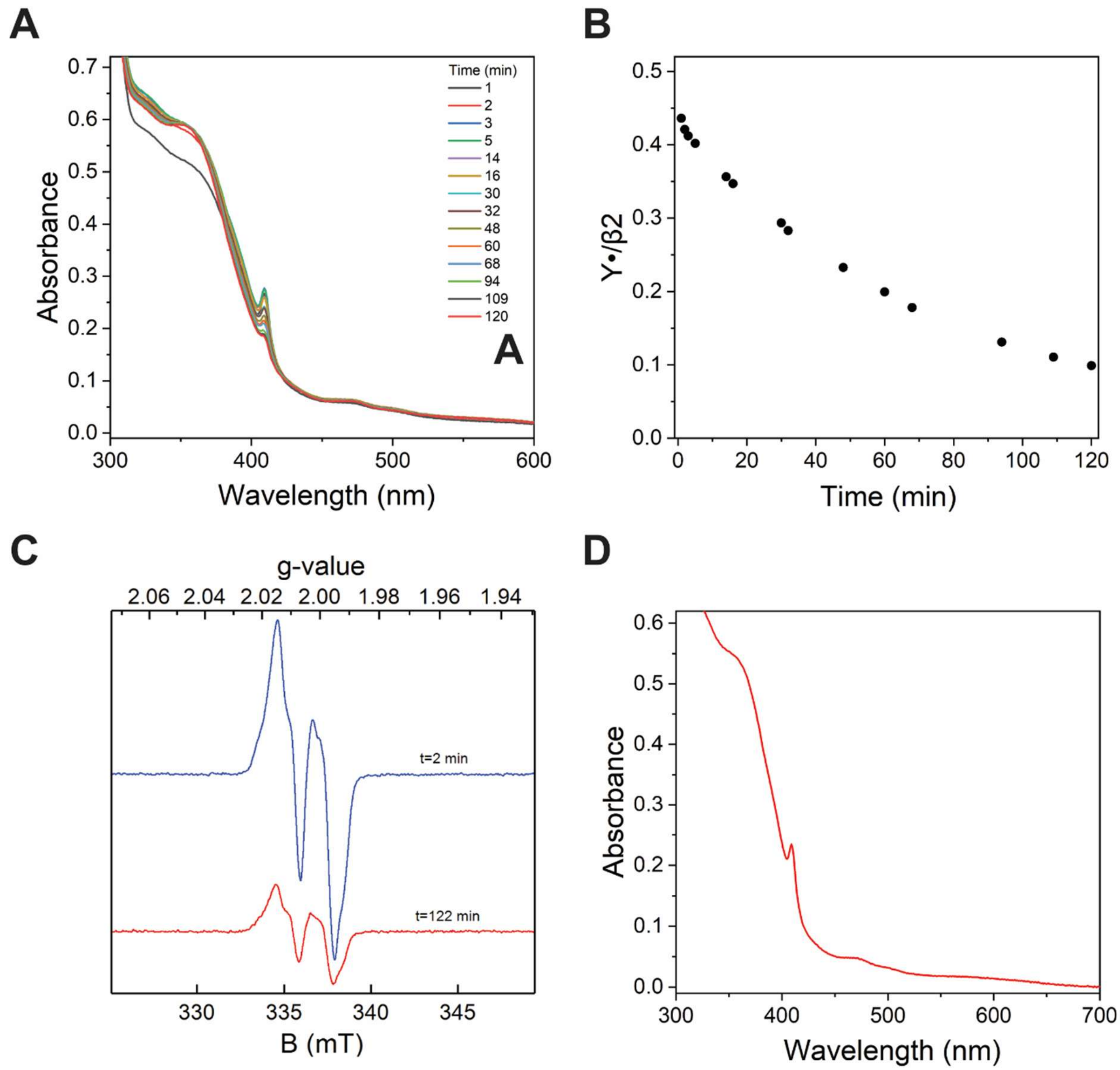

Figure S10. (A) Chromatogram of diferrous-β2, prepared anaerobically and activated by direct injection of 0.75 mL of 220 μM β2 onto an aerobic S200 analytical column (24 mL column volume, void volume ~7.5 mL), monitoring absorbance at 280 nm (protein) and 409 nm (diferric-Y• cofactor). The major peak elutes at approximately 14 mL, corresponding to approximately 75 kDa, consistent with the expected β2 dimer, with absorption at 409 nm showing assembly of metal cofactor. Minor peaks around 9 mL represent higher-molecular-weight aggregates. **(B)** UV-visible spectrum of the fractions pooled based on presence of the 409 nm absorbance peak, showing assembly of the diferric-Y• cofactor.

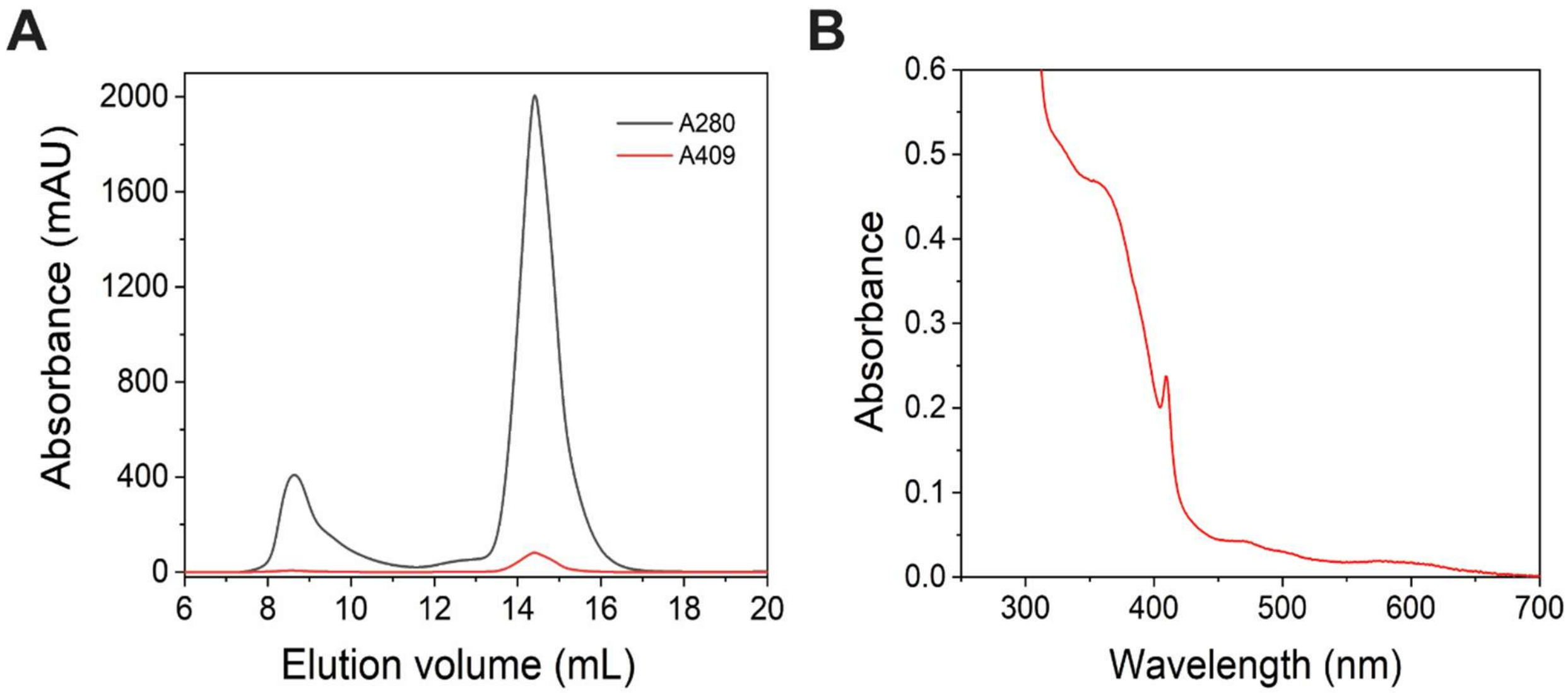

Figure S11. (A) UV-visible absorption spectra of diferrous-β2 (25 μM β2) incubated with 2 $NrdI_{hq}$/β2 and exposed to O_2. **(B)** Absorbance at 409 nm over time indicates Y• does not form in appreciable quantities.

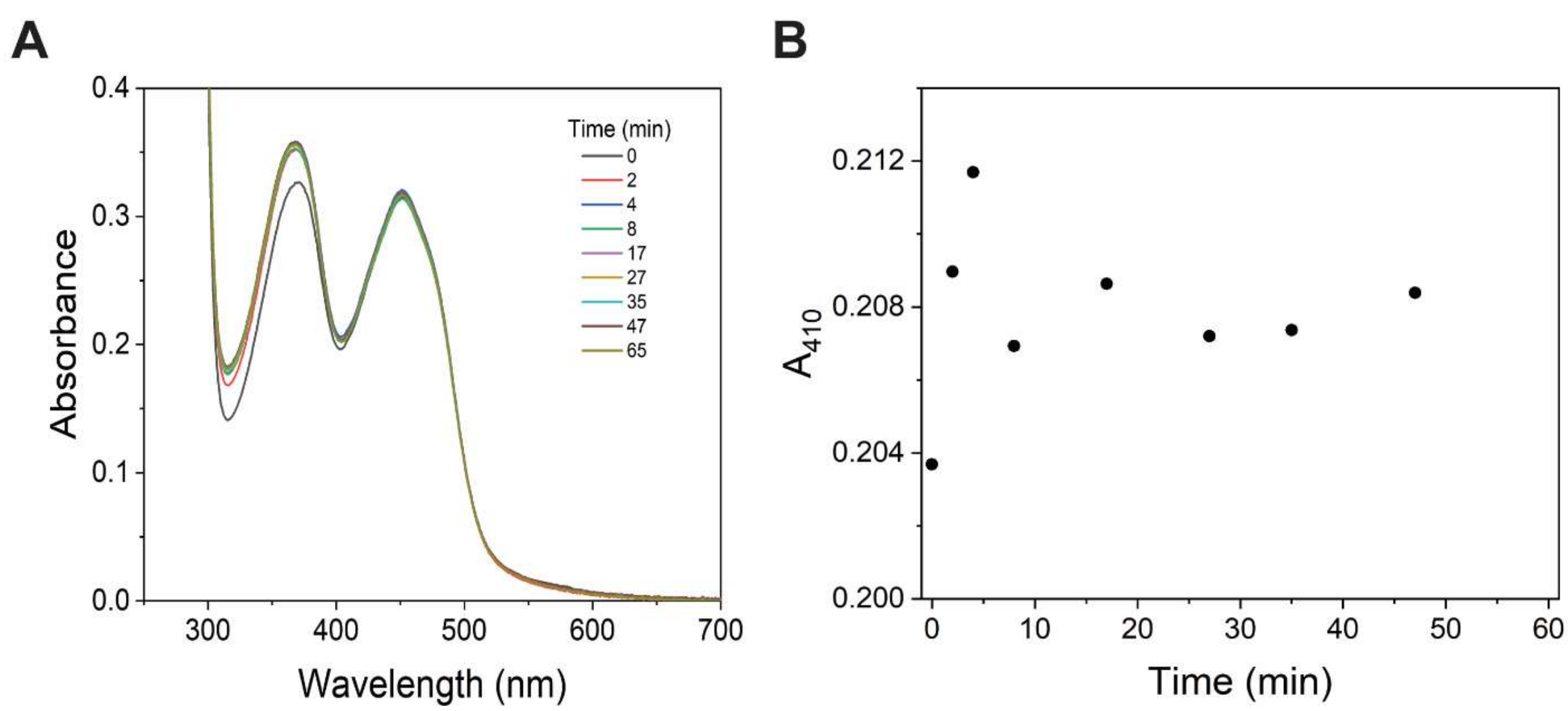

Figure S12. (A) Size exclusion chromatography (SEC) of manganese/NrdI-activated β partially separates unreacted β and improves Y•/β2 content. Mn^{II}-loaded β (50.5 µM β2) was activated by reaction of 2 $NrdI_{hq}$/β2 with excess O_2 at 25 °C. Chromatogram for Superdex 200 analytical separation of the reaction mixture (24 mL column volume, void volume ~7.5 mL). The primary contributor to absorbance at 454 nm is the FMN cofactor of NrdI. Analytical SEC of apo-β prior to activation shows that the aggregate eluting at 8-10 mL was not present in the apo preparation; therefore, it was generated during the activation procedure. **(B)** SDS-PAGE analysis of SEC fractions. Fractions 2–4 (eluting at 8-10 mL) show a band at ~40 kDa (aggregated β) and a faint band at 18 kDa (NrdI lacking its flavin cofactor, judging from the lack of a 454 nm absorbance). Fractions 7–8 (eluting at 13-15 mL, apparent MW = 110 kDa; β2:$NrdI_2$ complex would be 116 kDa) contain the expected bands at 40 kDa and 18 kDa. Fractions 9–10 (eluting at ~16-18 mL) correspond to inactive NrdI, without its FMN cofactor. The blue box indicates fractions containing Y•, as verified by UV-visible absorption spectra. Expected bands are at 40 kDa for β and 18 kDa for His_6NrdI, showing incomplete separation of NrdI from β2. **(C)** UV–vis spectra of the activated dimanganese-Y• cluster 40 min after activation, and pooled fractions 7–8 following Superdex 200 analytical separation.

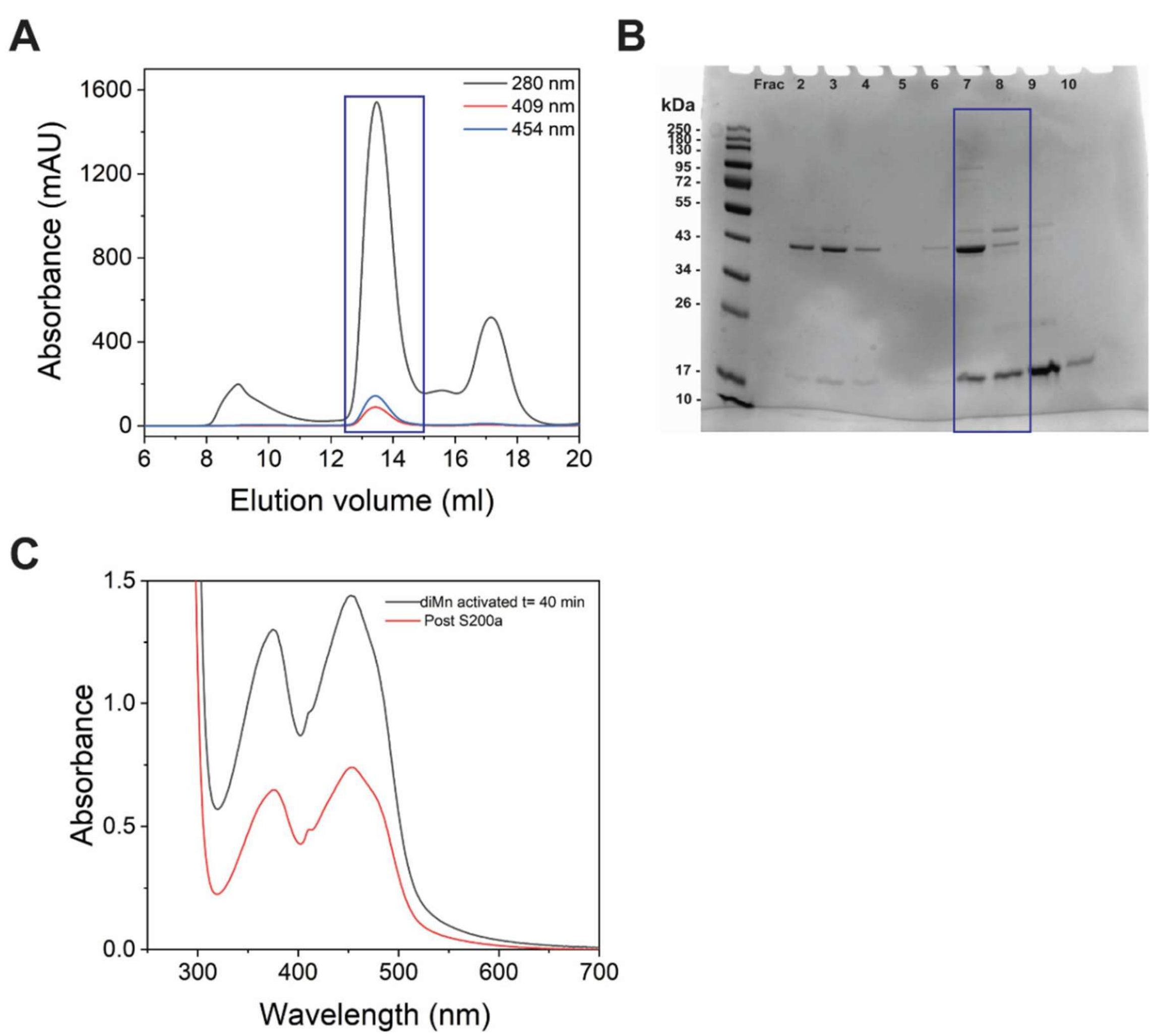

Figure S13. (A) X-band EPR spectrum of dimanganese(II)-β2 (49 μM, 4 Mn^{II}/β2) treated with 12.5% v/v Chelex-100 for 2 min, collected at 20 K with 1 mW power. **(B)** Quantification by ICP-MS of Mn retained by β2 (50 μM) preincubated with 4 Mn^{II}/β2 and treated with 12.5% v/v Chelex-100 at 4 °C on a nutating mixer over the course of 2 h. Two experiments were performed and each point is derived from 3 technical replicates.

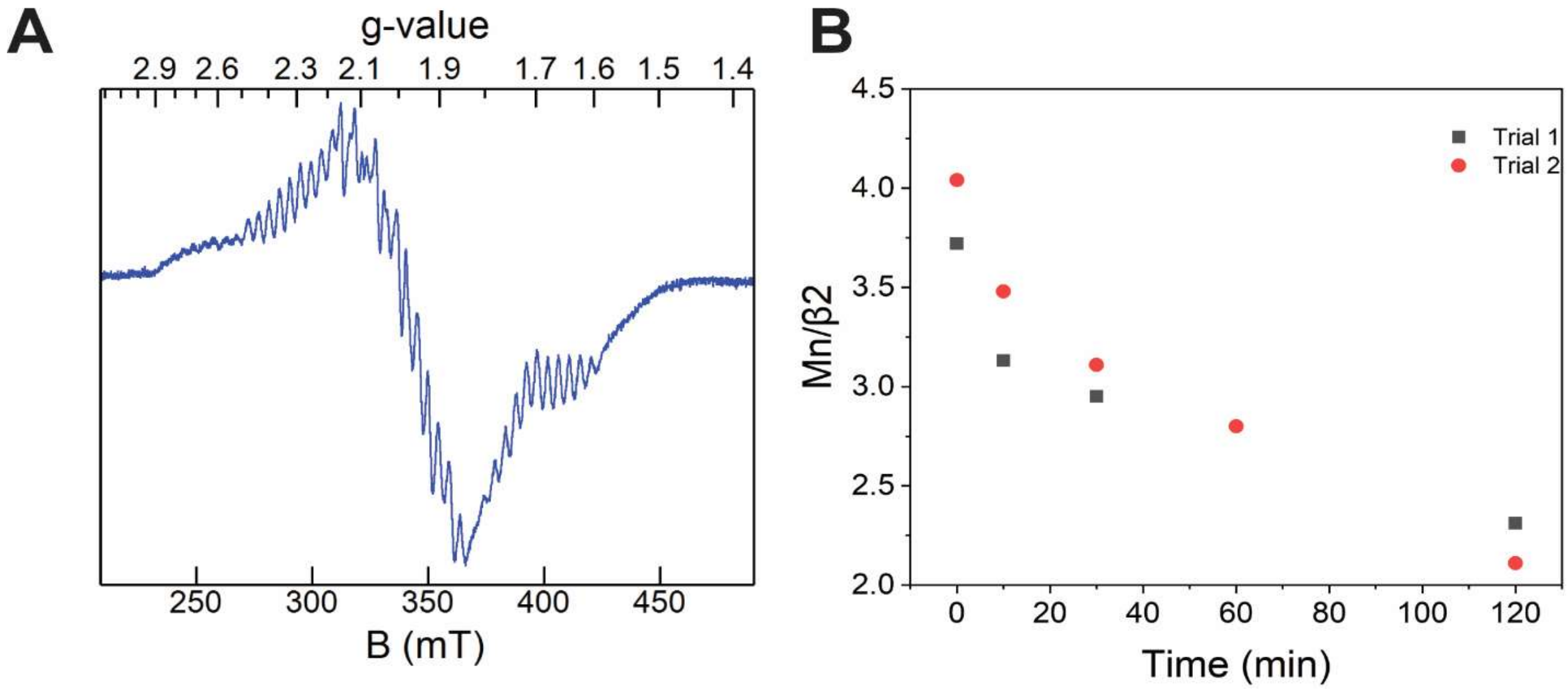

Figure S14. EPR spectra of apo β (50 μM) incubated with either 3.4 equiv. (red) or 4.0 equiv. (blue) Mn^{II} ions, measured at 20 K with 1 mW power and 0.35 mT modulation amplitude. The blue spectrum shows features indicative of mononuclear Mn^{II}, whereas the red spectrum shows only dimanganese(II) cluster.

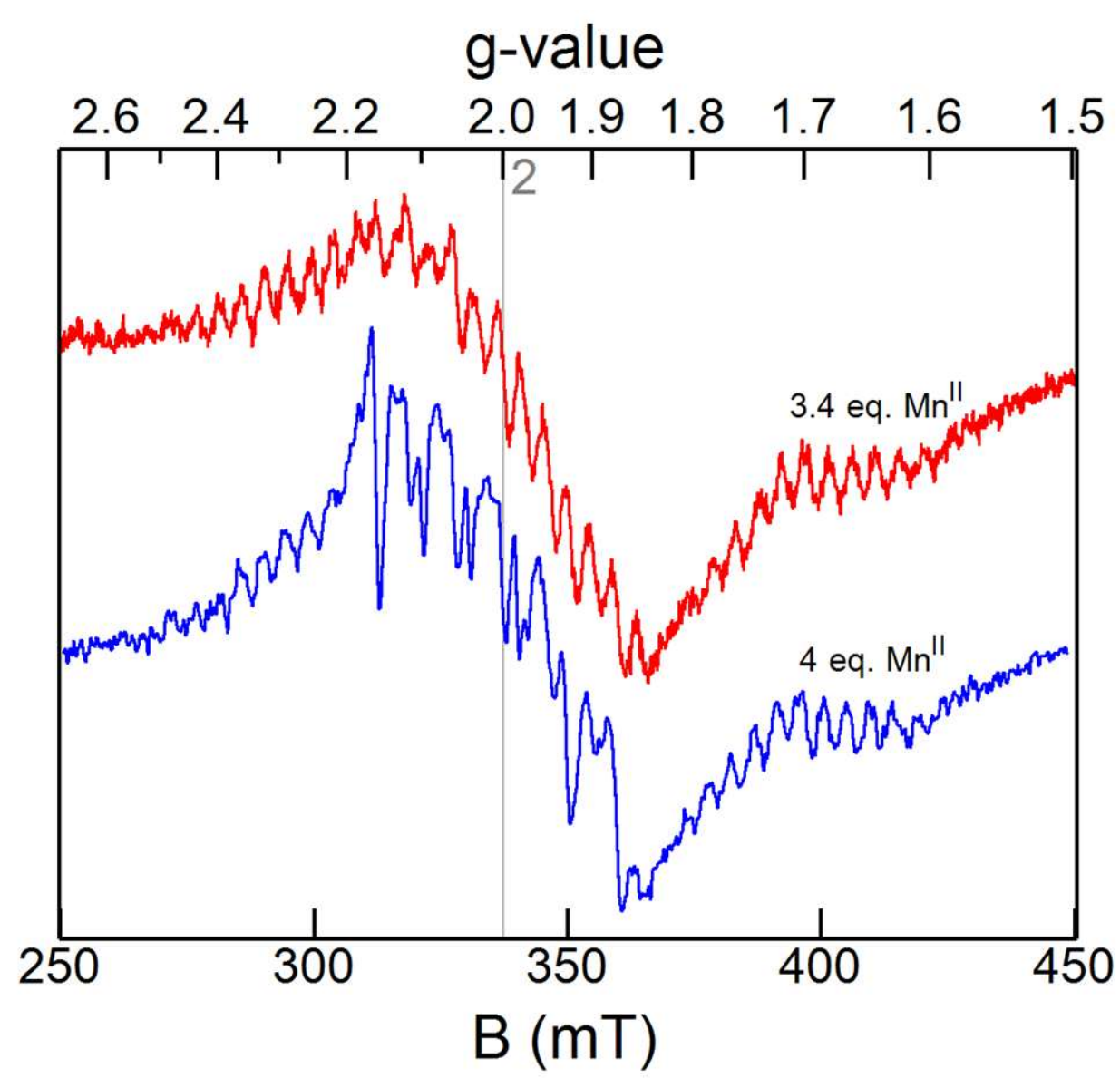

Figure S15. Variable temperature X-band EPR spectra of unactivated Mn_2^{II}-β2 (130 μM β2). **(A)** Temperature-dependent X-band EPR spectra of unactivated dimanganese cofactor prepared by addition of 3.4 Mn^{II}/β2. Temperatures from 10 K to 90 K were run with liquid He and temperatures from 90 K to 200 K were run with liquid N_2. All spectra were collected with 1 mW power and 0.35 mT modulation amplitude. **(B)** The double integral of EPR intensity from 200-450 mT multiplied by temperature, as a function of temperature, indicating antiferromagnetic coupling of the Mn^{II} ions.

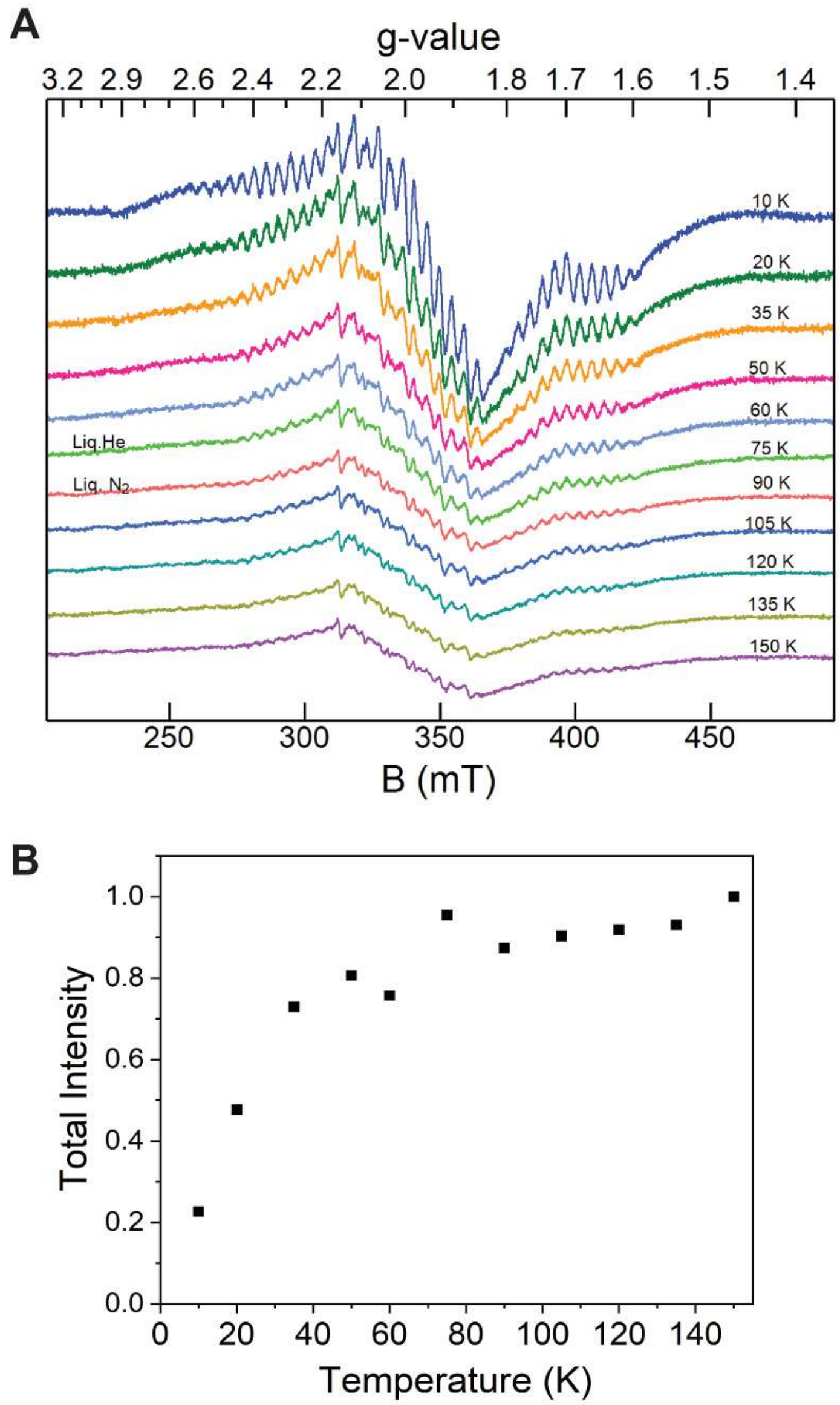

Figure S16. X-band EPR power sweep of dimanganese-Y• assembly (60 µM β2, 2 equiv. NrdI/β2) frozen at t = 90 min. Spectra were collected at T =10 K **(A)** and T = 100 K **(B)** with variable power and 0.35 mT modulation amplitude.

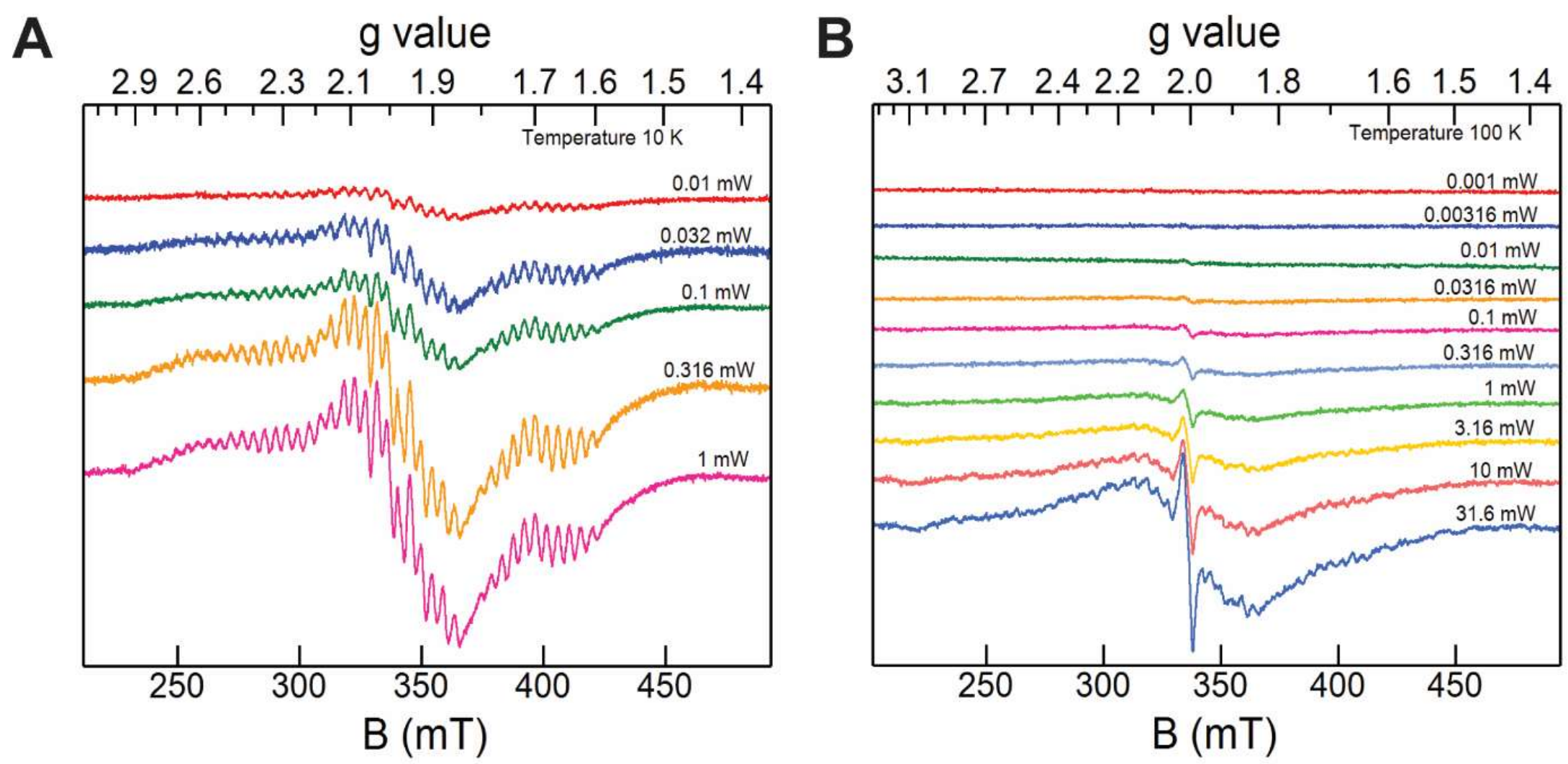

Figure S17. (A) EPR spectra of β2 (70 µM, 2 NrdI/β2) containing the Mn^{III}_2-Y• cofactor (red), collected at 150 K with 1 mW power and 0.35 mT modulation amplitude. Simulation is in black. **(B)** Signal zoomed in around 300-380 mT. Due to the broad dimanganese(II) signal in the background, the simulation will slightly overestimate Y• content.

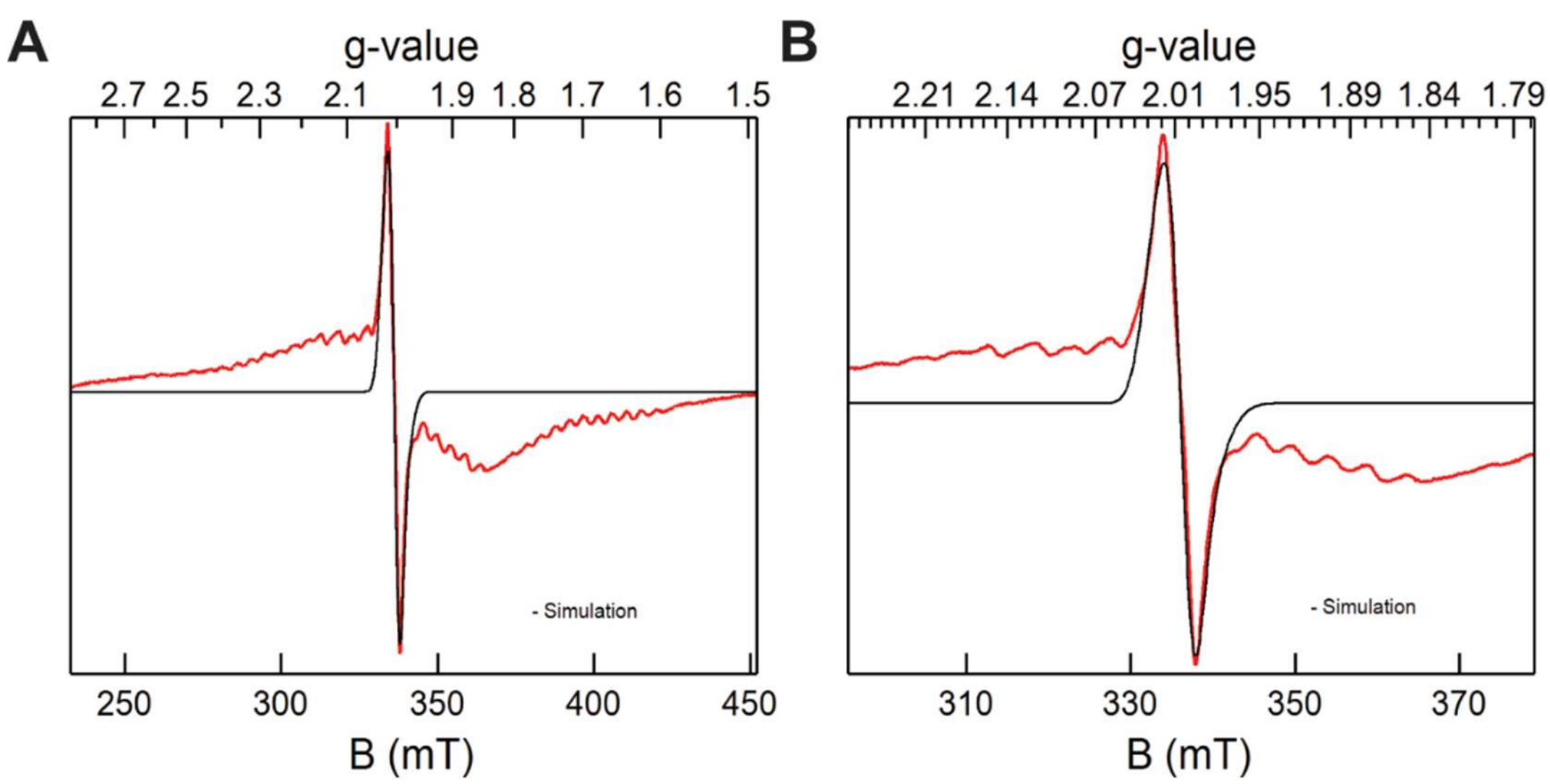

Figure S18. Simulation (black) of the EPR spectrum of the $Mn^{III}Mn^{IV}$ intermediate (pink) frozen 44 s after activation of 70 µM β2 (2 NrdI/β2) with O_2, collected at 20 K with 1 mW power and 0.35 mT modulation amplitude. Parameters for the simulation are A values of (-454, -454, -339) MHz and (229, 299, 263) MHz for the Mn^{III} and Mn^{IV} centers, respectively.

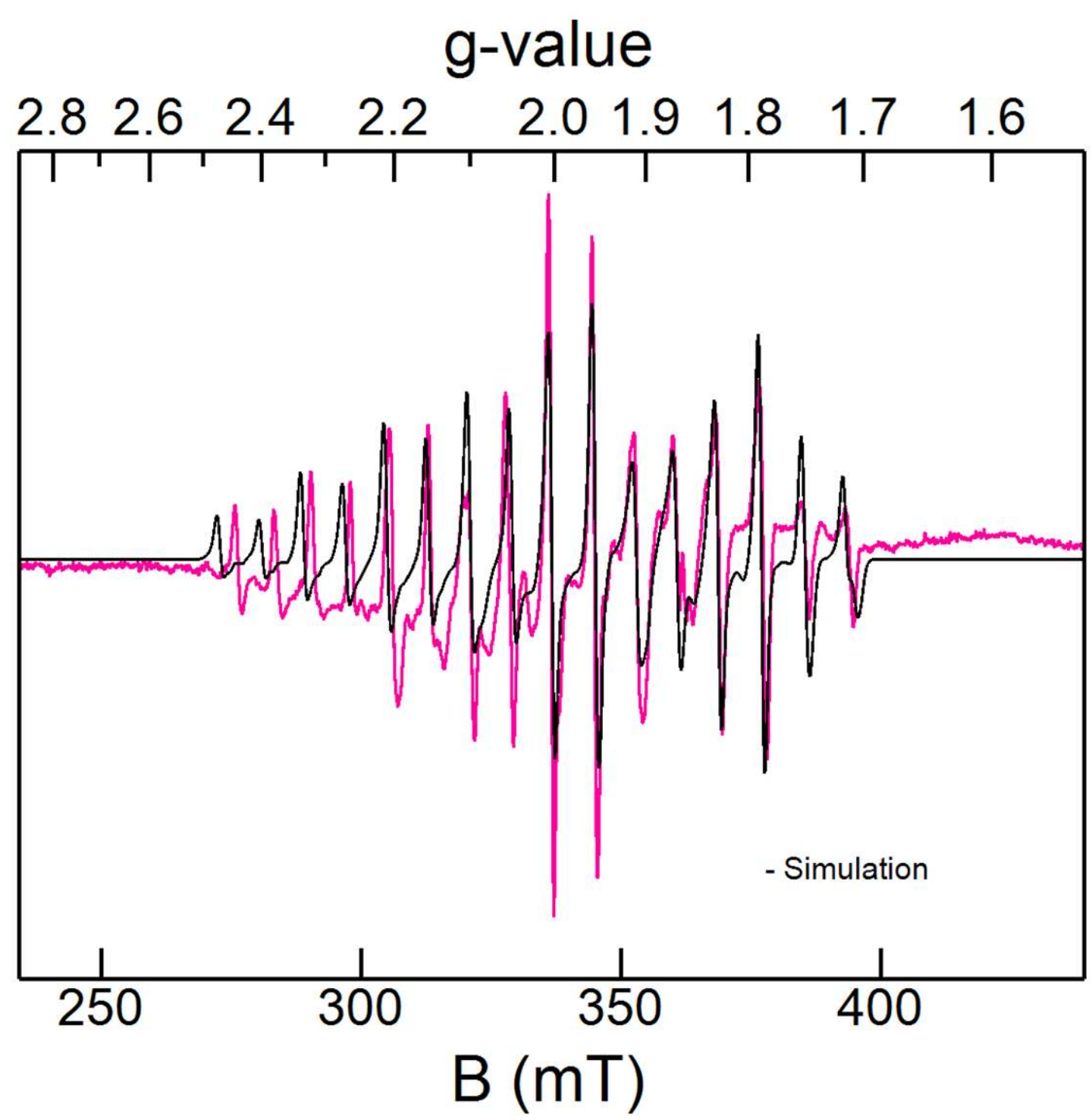

Figure S19. Comparison of dimanganese-Y• assembly reactions incubated at 37 °C (48 µM β2, 2 NrdI/β2) and at 25 °C (70 µM β2, 2 NrdI/β2). Y• formation was followed by absorbance at 410 nm. Higher temperature increases the rate constant for Y• formation by approximately 2-fold ($k = 0.0036\ s^{-1}$, $t_{1/2} = 3.2$ min).

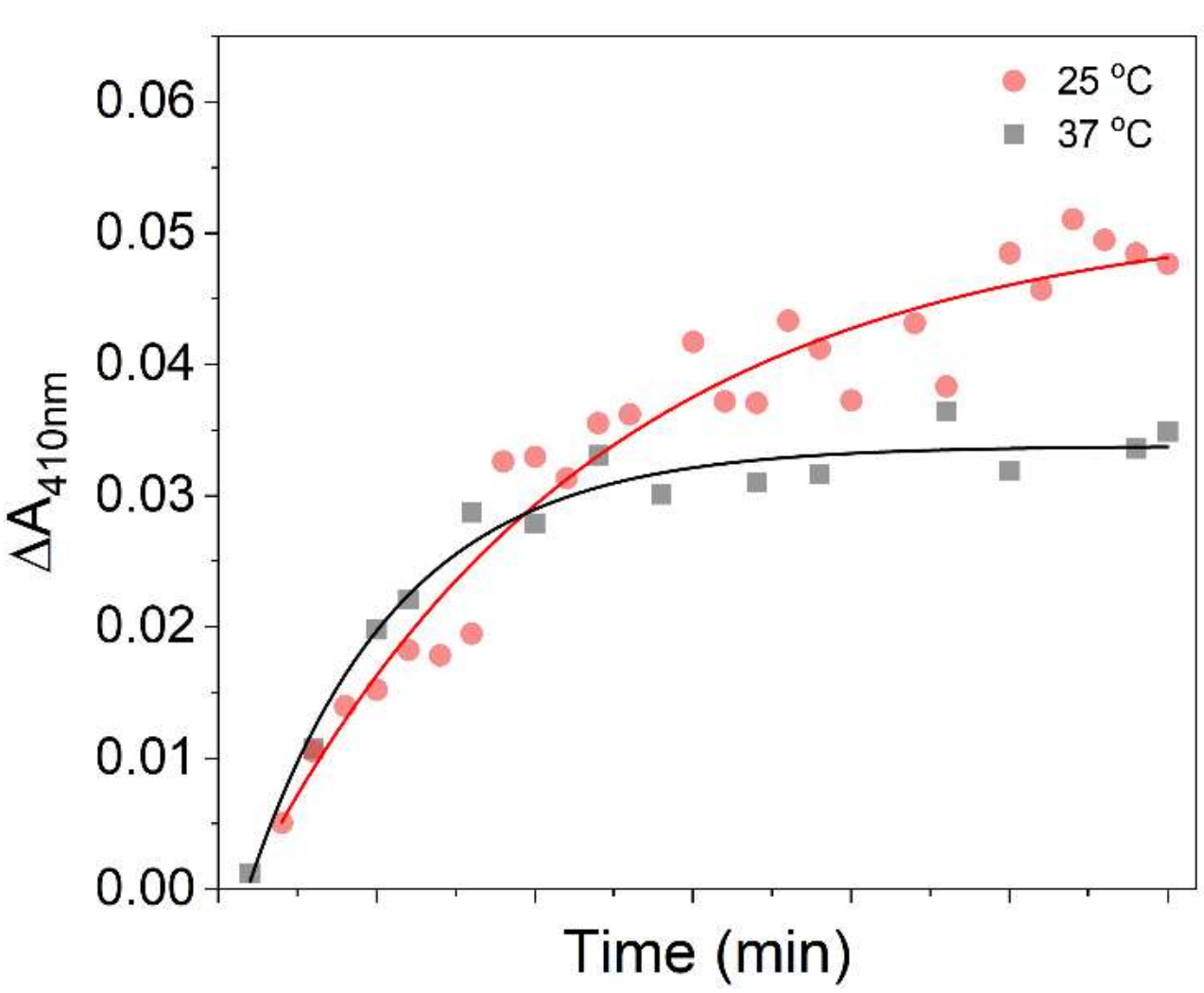

Figure S20. Replicate of dimanganese-Y• cofactor assembly monitored in parallel by UV-visible absorption and X-band EPR spectroscopy (70 µM β2, 2 equiv. NrdI/β2). **(A)** 20 K EPR spectra of samples quenched at various timepoints. **(B)** 150 K EPR spectra. A small amount of mononuclear Mn^{II} signal is apparent at this temperature. **(C)** UV-visible absorbance at 410 nm of dimanganese-Y• cofactor assembly, run in parallel with the X-band EPR samples. **(D)** Kinetic traces for Y• formation and $Mn^{III}Mn^{IV}$ decay following cofactor activation as quantified by EPR, with single-exponential fits (see **Table S3**, #3).

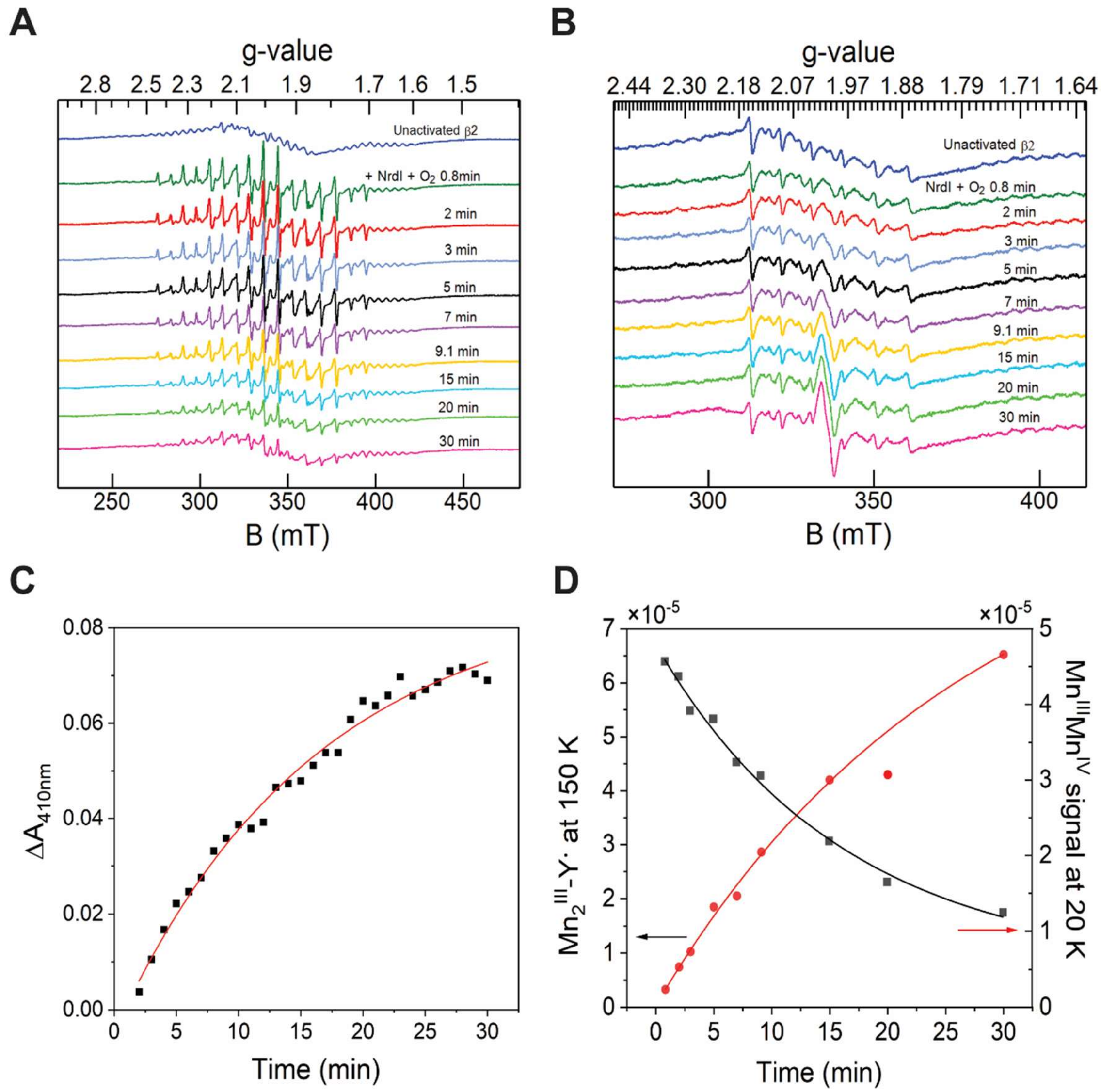

Figure S21. (A) X-band EPR spectra of $Mn^{II}Zn^{II}$ cofactors assembled by incubation of 70 µM β2 with 3.4 Mn/β2 for 10 min followed by incubation with varied molar equivalents of Zn^{II} for 10 min, followed by a 5-min treatment with Chelex and desalting to remove unbound metal ions. Spectra have been normalized to the intensity of 3.4 Mn^{II}/β2 + 1.6 Zn^{II}/β2, with the normalization factors shown at right. 3.4 equiv. Zn^{II} are required to reduce the intensity of the mononuclear Mn^{II} signal by 20%. Spectra were collected at 20 K with 1 mW power and 0.35 mT modulation amplitude. **(B)** CW X-Band EPR spectra of the 3.4 Mn^{II}/β2 + 1.7 Zn^{II}/β2 sample activated with 2 $NrdI_{hq}$/β2 and excess O_2, with spectra acquired at T = 20 K (blue) and T = 150 K (red). **(C)** Variable temperature X-Band EPR spectra of 124 µM β2 activated with 1.7 equiv. Mn^{II} + 1.7 equiv. Fe^{II}, 2 NrdI/β2, and O_2, 50 min post-O_2 addition, concentrated and treated with Chelex for 5 min. Temperatures from 10 K to 90 K were run with liquid He and temperatures from 90 K to 200 K were run with liquid N_2. All spectra were collected with 1 mW power and 0.35 mT modulation amplitude. **(D)** X-Band EPR power sweep of the same sample as in (C). Spectra were collected at 10 K (top) with variable power with 0.35 mT modulation amplitude.

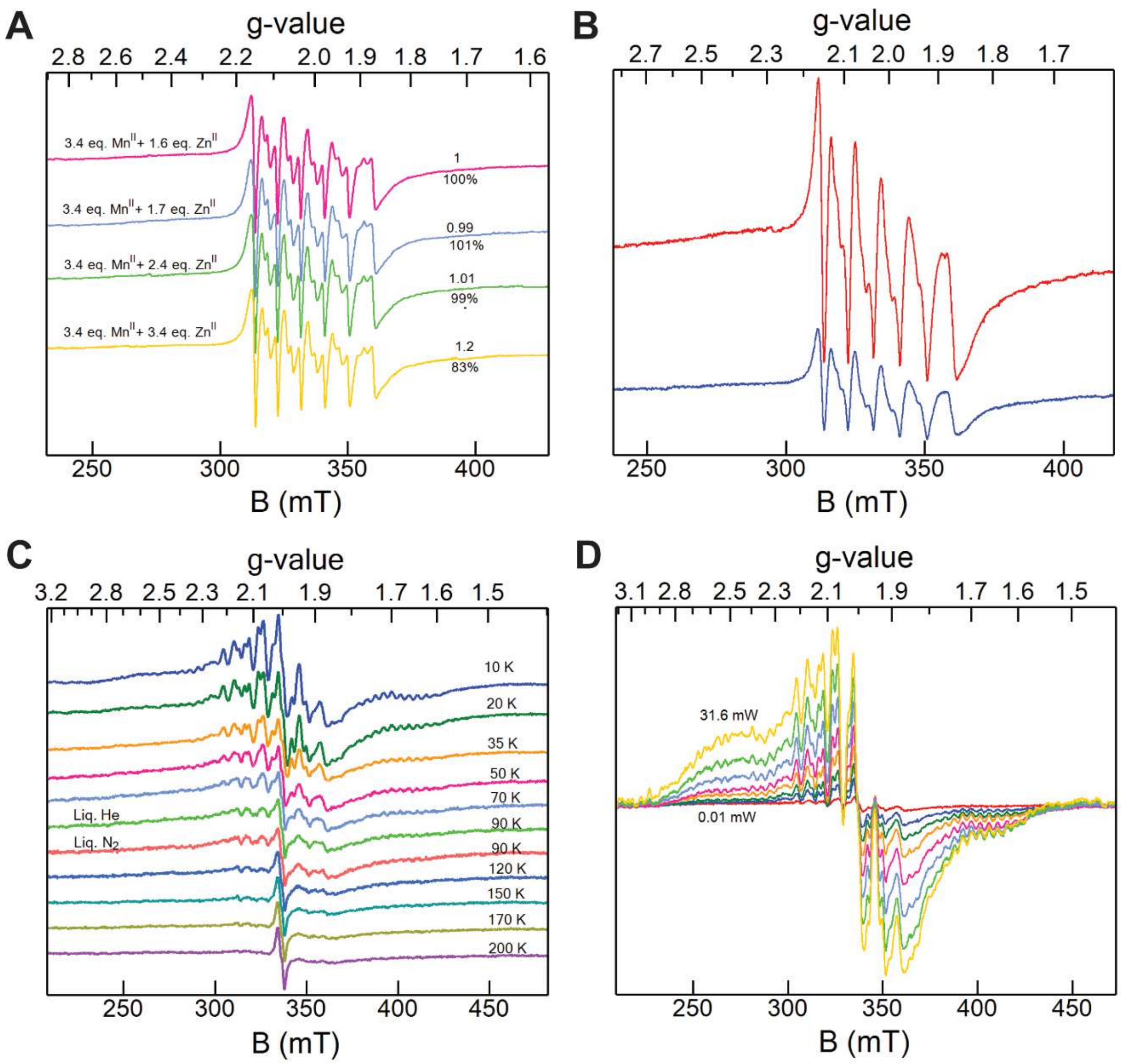

Figure S22. dCDP production determined by LC/MS analysis of reactions of β2 after MnFe cofactor assembly, when assayed with a 5-fold excess SUMO-α2 and DTT, and in the presence of TrxL (+) or absence of TrxL (–). The slopes of the lines of best fit were used to calculate the rate of dCDP formation and used to calculate the specific activities reported in **Table 1**.

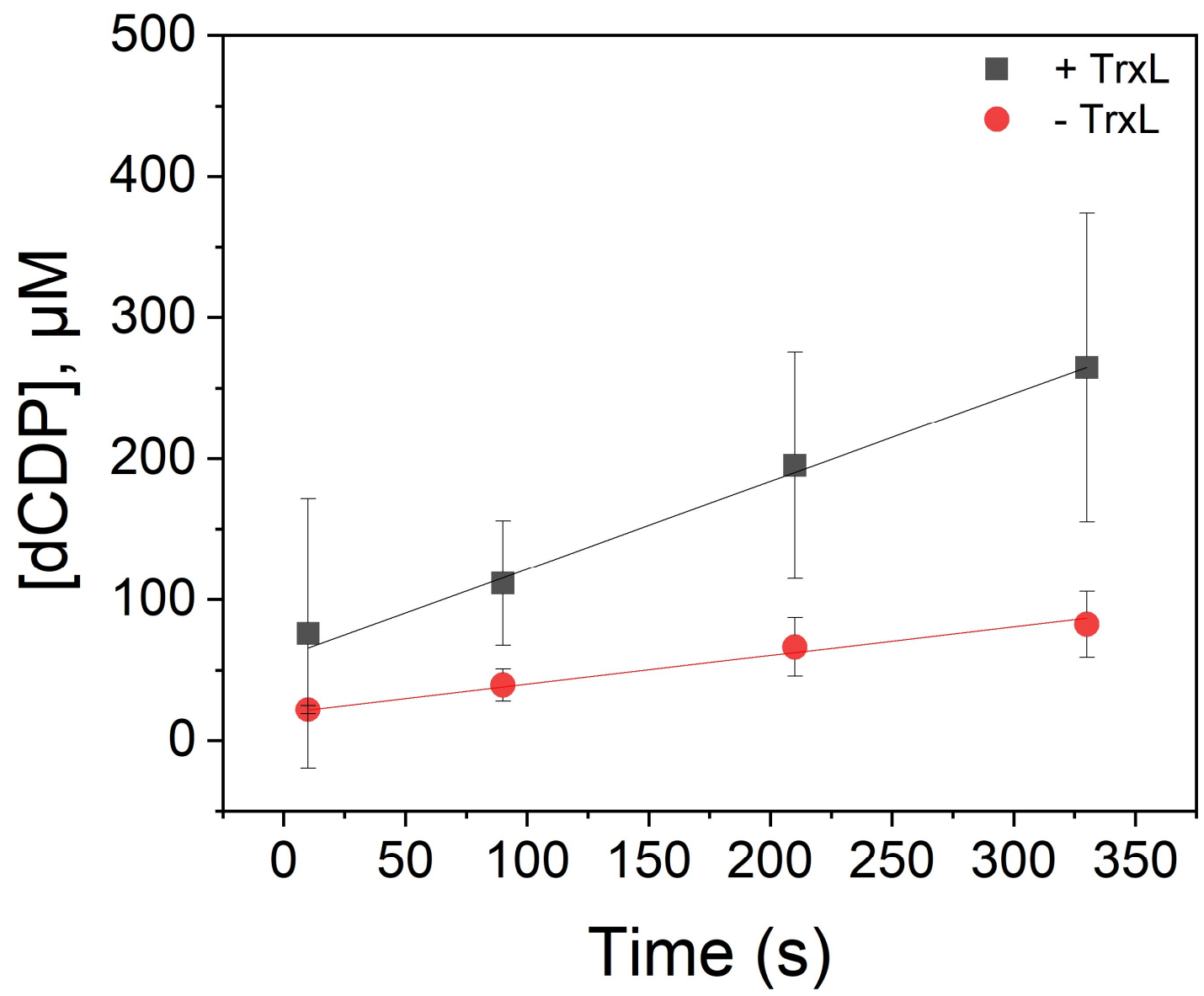

Figure S23. UV-visible absorbance at 410 nm of dimanganese-Y• cofactor assembly reaction in presence of SUMO-α and ATP; 30 μM Mn^{II}_2-β2 and 60 μM reduced were NrdI mixed with O_2, followed by addition of SUMO-α (60 μM) and ATP (2 mM).

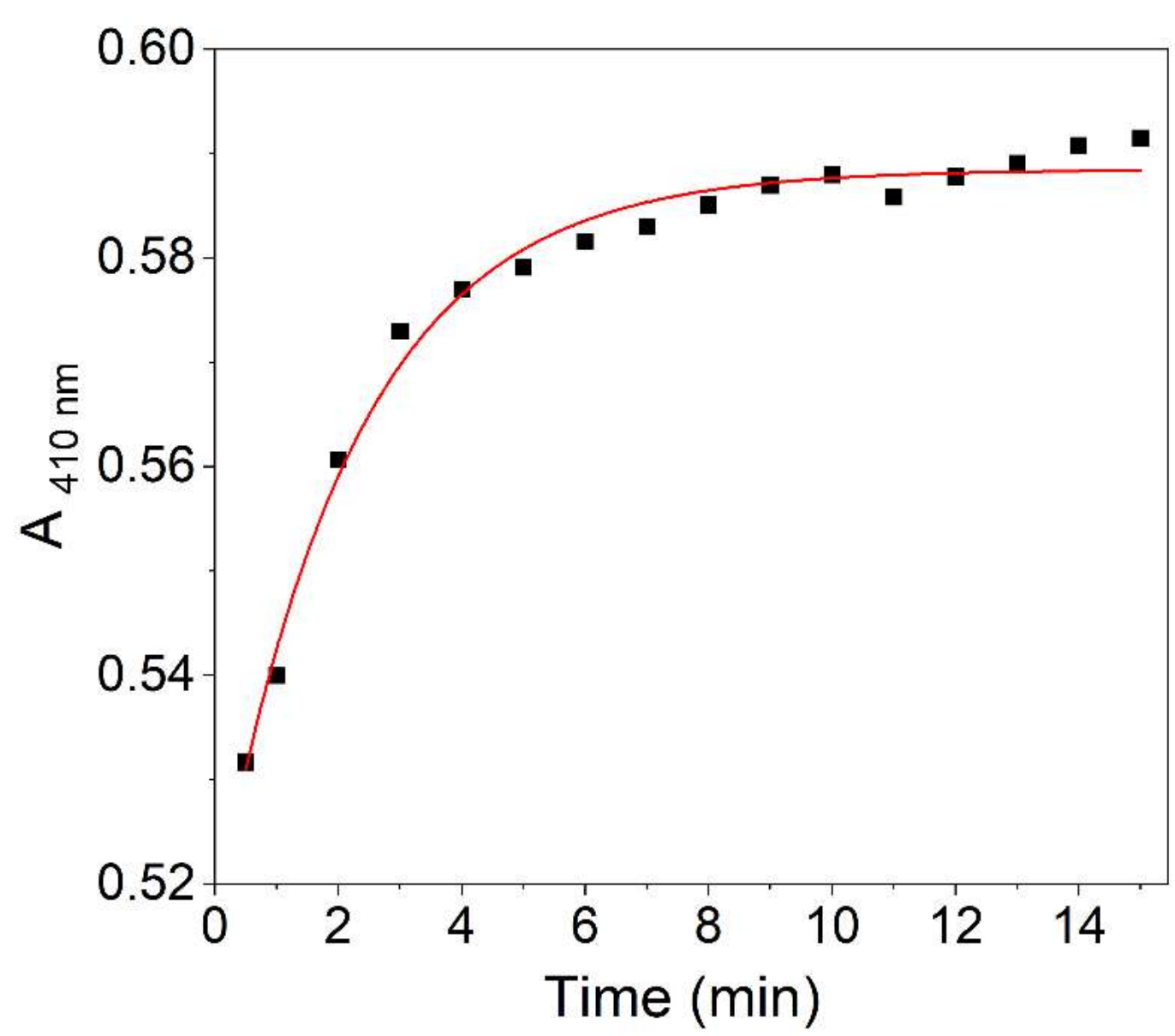

Figure S24. Chain A from the x-ray crystal structure of *Bha* β (PDB code: 2RCC), in which metal ions were not modeled. Core helix 1, including the unwound region from D88 to D93, is shown in dark blue, and metal-binding residues from (**Figure 1C**) are shown in sticks. Electron density from the D88 side chain is not modeled in the PDB file. The asymmetric unit of the x-ray crystal structure of *Bha* β contains three chains: the apo chain shown here and two chains each with two metal ions modeled as Zn^{II} occupying their dinuclear metal-binding site (**Figure 1C**). If indeed they are Zn^{II} ions, we speculate that their presence may reflect inability to assemble the correct metallocofactor during heterologous expression in *E. coli* and non-specific binding of Zn^{II} during purification.

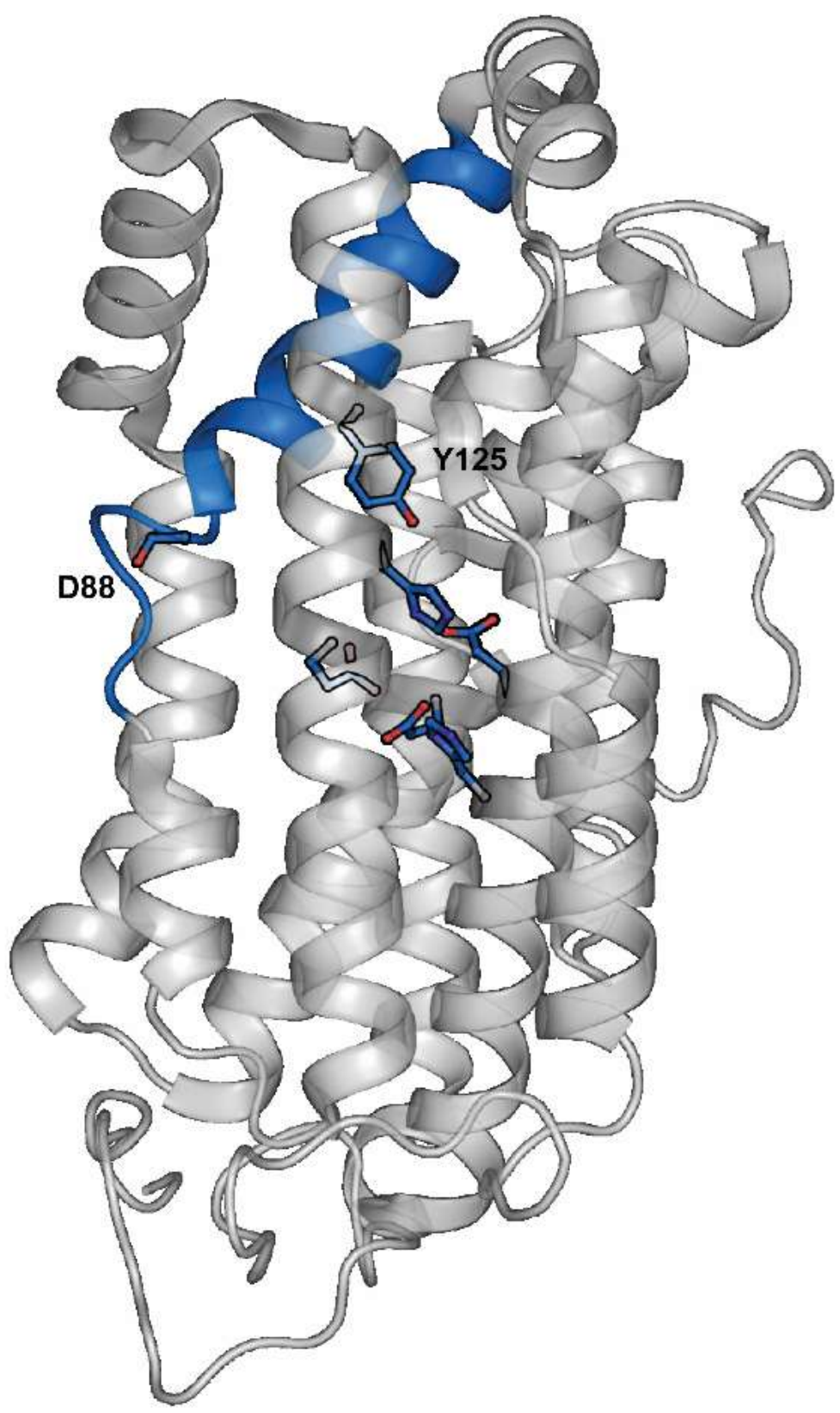

SUPPLEMENTARY REFERENCES

(1) Gasteiger, E.; Hoogland, C.; Gattiker, A.; Duvaud, S. e.; Wilkins, M. R.; Appel, R. D.; Bairoch, A. Protein Identification and Analysis Tools on the ExPASy Server. In *The Proteomics Protocols Handbook*, Walker, J. M. Ed.; Humana Press, 2005; pp 571-607.
(2) Cotruvo, J. A.; Stubbe, J. NrdI, a flavodoxin involved in maintenance of the diferric-tyrosyl radical cofactor in *Escherichia coli* class Ib ribonucleotide reductase. *Proc. Natl. Acad. Sci. U.S.A.* **2008**, *105* (38), 14383-14388. DOI: 10.1073/pnas.0807348105.
(3) Bollinger, J. M.; Tong, W. H.; Ravi, N.; Huynh, B. H.; Edmondson, D. E.; Stubbe, J. A. Use of rapid kinetics methods to study the assembly of the diferric-tyrosyl radical cofactor of *E. coli* ribonucleotide reductase. *Methods in Enzymology* **1995**, *258*, 278-303. DOI: 10.1016/0076-6879(95)58052-2.
(4) Cotruvo, J. A.; Stich, T. A.; Britt, R. D.; Stubbe, J. Mechanism of assembly of the dimanganese-tyrosyl radical cofactor of class Ib ribonucleotide reductase: Enzymatic generation of superoxide is required for tyrosine oxidation via a Mn(III)Mn(IV) intermediate. *J. Am. Chem. Soc.* **2013**, *135* (10), 4027-4039. DOI: 10.1021/ja312457t.
(5) Cotruvo, J. A.; Stubbe, J. An active dimanganese(III)-tyrosyl radical cofactor in *Escherichia coli* class Ib ribonucleotide reductase. *Biochemistry* **2010**, *49* (6), 1297-1309. DOI: 10.1021/bi902106n.
(6) Roca, I.; Torrents, E.; Sahlin, M.; Gibert, I.; Sjöberg, B.-M. NrdI Essentiality for Class Ib Ribonucleotide Reduction in *Streptococcus pyogenes*. *J. Bacteriol.* **2008**, *190* (14), 4849-4858. DOI: 10.1128/jb.00185-08.
(7) Blaesi, E. J.; Palowitch, G. M.; Hu, K.; Kim, A. J.; Rose, H. R.; Alapati, R.; Lougee, M. G.; Kim, H. J.; Taguchi, A. T.; Tan, K. O.; et al. Metal-free class Ie ribonucleotide reductase from pathogens initiates catalysis with a tyrosinederived dihydroxyphenylalanine radical. *Proc. Natl. Acad. Sci. U.S.A.* **2018**, *115* (40), 10022-10027. DOI: 10.1073/PNAS.1811993115.
(8) Petasis, D. T.; Hendrich, M. P. Chapter Eight - Quantitative Interpretation of Multifrequency Multimode EPR Spectra of Metal Containing Proteins, Enzymes, and Biomimetic Complexes. In *Methods in Enzymology*, Qin, P. Z., Warncke, K. Eds.; Vol. 563; Academic Press, 2015; pp 171-208.
(9) Golombek, A. P.; Hendrich, M. P. Quantitative analysis of dinuclear manganese(II) EPR spectra. *J. Magn. Reson.* **2003**, *165* (1), 33-48. DOI: 10.1016/j.jmr.2003.07.001.
(10) Sun, X.; Ou, Z.; Chen, R.; Niu, X.; Chen, D.; Kang, R.; Tang, D. Activation of the p62-Keap1-NRF2 pathway protects against ferroptosis in hepatocellular carcinoma cells. *Hepatology* **2016**, *63* (1), 173-184. DOI: 10.1002/hep.28251.
(11) Weil, J. A.; Bolton, J. R.; Wertz, J. E. *Electron Paramagnetic Resonance: Elementary Theory and Practical Applications*; Wiley, 1994.
(12) Stoll, S.; Schweiger, A. EasySpin, a comprehensive software package for spectral simulation and analysis in EPR. *J. Magn. Reson.* **2006**, *178* (1), 42-55. DOI: https://doi.org/10.1016/j.jmr.2005.08.013.
(13) Tomter, A. B.; Zoppellaro, G.; Andersen, N. H.; Hersleth, H.-P.; Hammerstad, M.; Røhr, Å. K.; Sandvik, G. K.; Strand, K. R.; Nilsson, G. E.; Bell, C. B.; et al. Ribonucleotide reductase class I with different radical generating clusters. *Coord. Chem. Rev.* **2013**, *257* (1), 3-26. DOI: 10.1016/j.ccr.2012.05.021.
(14) Liu, A.; Pötsch, S.; Davydov, A.; Barra, A.-L.; Rubin, H.; Gräslund, A. The Tyrosyl Free Radical of Recombinant Ribonucleotide Reductase from *Mycobacterium tuberculosis* Is Located

in a Rigid Hydrophobic Pocket. *Biochemistry* **1998**, *37* (46), 16369-16377. DOI: 10.1021/bi981471p.
(15) Martinie, R. J.; Livada, J.; Kothiya, N.; Bollinger, J. M., Jr.; Krebs, C.; Silakov, A. Structural Elucidation of the Reduced Mn(III)/Fe(III) Intermediate of the Radical-Initiating Metallocofactor in *Chlamydia trachomatis* Ribonucleotide Reductase. *Biochemistry* **2025**, *64* (5), 1157-1167. DOI: 10.1021/acs.biochem.4c00692.
(16) Jiang, W.; Yun, D.; Saleh, L.; Barr, E. W.; Xing, G.; Hoffart, L. M.; Maslak, M.-A.; Krebs, C.; Bollinger, J. M. A Manganese(IV)/Iron(III) Cofactor in *Chlamydia trachomatis* Ribonucleotide Reductase. *Science* **2007**, *316* (5828), 1188-1191. DOI: 10.1126/SCIENCE.1141179.
(17) Jiang, W.; Hoffart, L. M.; Krebs, C.; Bollinger, J. M. A manganese(IV)/iron(IV) intermediate in assembly of the manganese(IV)/iron(III) cofactor of *Chlamydia trachomatis* ribonucleotide reductase. *Biochemistry* **2007**, *46* (30), 8709-8716. DOI: 10.1021/BI700906G.
(18) Bou-Abdallah, F.; Chasteen, N. D. Spin concentration measurements of high-spin (g′ = 4.3) rhombic iron(III) ions in biological samples: theory and application. *J. Biol. Inorg. Chem.* **2008**, *13* (1), 15-24. DOI: 10.1007/s00775-007-0304-0.
